# Synthesis and Antiproliferative Effects of Grossheimin-Derived Aminoanalogues

**DOI:** 10.3390/biom15040578

**Published:** 2025-04-14

**Authors:** Meruyert Ashimbayeva, Zsolt Szakonyi, Sergazy M. Adekenov, Nikoletta Szemerédi, Gabriella Spengler, Tam Minh Le

**Affiliations:** 1 Institute of Pharmaceutical Chemistry, University of Szeged, Eötvös u. 6, H-6720 Szeged, Hungary; mikosha.3791@mail.ru; 2 JSC Research and Production Center “Phytochemistry”, Karaganda 100009, Kazakhstan; arglabin@phyto.kz; 3 Department of Medical Microbiology, Albert Szent-Györgyi Health Center and Albert Szent-Györgyi Medical School, University of Szeged, Semmelweis utca 6, H-6725 Szeged, Hungary; szemeredi.nikoletta@med.u-szeged.hu (N.S.); spengler.gabriella@med.u-szeged.hu (G.S.); 4 HUN-REN–SZTE Stereochemistry Research Group, University of Szeged, Eötvös u. 6, H-6720 Szeged, Hungary

**Keywords:** grossheimin, aminoanalogues, cytotoxic, Michael addition, amino lactone, colon adenocarcinoma

## Abstract

Grossheimin, a guaiane-type sesquiterpene lactone, displayed a diverse range of biological activities, including anticancer, anti-inflammatory and antimicrobial effects. Various amino analogues of grossheimin were prepared through a Michael addition at its highly active α-methylene-γ-lactone motif. On the other hand, grossheimin was reduced to diol, which was then subjected to nucleophilic addition or acetylation to introduce heteroatoms associated with oxygen, sulfur or nitrogen functionalities. All of the synthesised Michael and acetylated adducts were evaluated for their in vitro cytotoxic action on human colon adenocarcinoma lines, including Colo205 and Colo320. The bioassay results indicated that the acetylated adducts displayed a potent cytotoxic effect compared to grossheimin, the parent molecule. A docking study was also performed to exploit the observed results.

## 1. Introduction

Colorectal cancer (CRC) is the third most common malignancy globally and one of the leading causes of mortality among cancer patients [1]. The global incidence of colorectal cancer in 2020 was approximately 1.9 million cases, resulting in roughly one million fatalities [2]. The current clinical treatment for CRC mainly involves surgery and chemotherapy [3]. Although 5-fluorouracil (5-FU) and oxaliplatin are used clinically for the treatment of CRC, the effects of cytotoxicity, drug resistance and adverse reactions are the main problems associated with chemotherapy [4]. Therefore, novel therapeutic strategies are constantly needed to improve the safety and effectiveness of CRC therapy.

Guaianolides, as secondary metabolites, constitute one of the largest groups of naturally occurring sesquiterpene lactones with structural complexity and a wide range of biological activities [5]. Grossheimin (**1**), an abundant and active guaianolide, was first isolated in *Grossheimia macrocephala* (Muss.-Puschk. ex Willd.) and *G. ossica* (C. Koch) Sosn. et Takht and also presented as a minor constituent in other asteraceous plants, such as *Amberboa lipii* DC., *Chartolepis biebersteinii* Jaub. Et Spach., *Ch. glastifolia* (L.) Cass., *Ch. intermedia* Boiss., *Ch. pterocaula* Trautv., *Centaurea behen* Linn., *C. helenioides* Boiss., *C. lippii* L., *C. ornata* Willd., *C. ruthenica* Lam., *C. scabiosa* L., *Cynara cardunculus* L., *C. scolymus* L., *Crepis virens* Vill., *Venidium decurrens* Less., and *Youngia japonica* (L.) DC [6]. Conversely, grossheimin, a major constituent from *Chartolepis intermedia* Boiss. (*C. intermedia* Boiss.) in Kazakhstan (ca. 0.1%) [7], is widely reported as a phytotoxic compound [8,9,10,11,12]. Furthermore, this compound is also receiving attention as a result of its reputed pharmacological properties beyond its function as a hypolipidemic [13] and anti-inflammatory agent [14,15]. Grossheimin has also been proven to possess anti-opisthorchiasis [16], antiobesity [17,18], antiallergic [19], antihyperlipidemic [20] and cytotoxic effects [21]. In addition, the high antitumor, antiparasitic, antioxidant and antimicrobial potential of grossheimin has been previously reported in the literature [22].

The structure–activity relationship (SAR) has indicated that the *α*-methylene-*γ*-butyrolactone moiety plays a significant role in determining the biological properties [23,24]. However, *α*-methylene-*γ*-lactone can exhibit non-selective binding as a Michael acceptor with undesired targets [25]. Therefore, a strategy employing reactions such as a Michael addition [26,27,28,29,30,31,32], Heck reaction [6,33] and others has been developed in which the reactive *α, β*-unsaturated enone is masked. These semi-synthetic modifications not only improve the pharmacokinetic profile but also maintain or enhance the biological activity of the parent molecule [34]. In addition to the electrophilic site, the secondary hydroxy group may serve as a nucleophilic site for halogenation [6,35] or acylation. Furthermore, Mo and co-workers recently reported that grossheimin displayed potential anticancer activity against MDA-MB-231 and colorectal cancer HCT-116 cell lines with an IC_50_ value of 16.86 and 30.94 μmol/L, respectively [36]. Stimulated by this result, and with the aim of finding novel biologically active grossheimin-based adducts that exert anti-cancer effects against a variety of CRC, a library of amino analogues has been prepared and studied for their cytotoxic activity on human colon adenocarcinoma lines. A docking model has also been developed for the most potent analogues.

## 2. Materials and Methods

### 2.1. Plant Materials

The air-dry aerial part (flower heads, buds and leaves) of *Chartolepis intermedia* Boiss. was collected in July 2023 in the vicinity of the village of Akbastau, Abay district, Karaganda region, Republic of Kazakhstan. A voucher specimen has been archived in the herbarium fund of the JSC Research and Production Center “Phytochemistry” (KG) (Karaganda, Republic of Kazakhstan).

#### Isolation of Grossheimin

The powdered raw material (0.2 kg) was pre-soaked for 10 min utilising a 1:1 mixture of EtOH:H_2_O (400 mL) followed by extraction with a laboratory ultrasonic system (HO-230.00 at an ultrasonic frequency of 22 kHz at 20–22 °C) for 90 min and a sequential extraction with CHCl_3_, resulting in an odorous dark green oil (3.1 g, 1.55%). The crude extract was subjected to gravity column chromatography on silica gel and eluted with a petroleum ether (PE)–EtOAc solvent system (*v*/*v*, 0:100, 100:0). Grossheimin **1** (2.4 g, 1.2%) as colourless crystals was obtained in the fraction that was purified by PE-EtOAc = 4:1. All of the physical and spectroscopic properties of grossheimin were similar to those described in the literature [6].

### 2.2. Synthetic Part

All of the solvents from the suppliers (Molar Chemicals Ltd., Halásztelek, Hungary; Merck Ltd., Budapest, Hungary and VWR International Ltd., Debrecen, Hungary) were dried following the standard procedures before use, while all of the reagents were obtained from commercial suppliers and used without further purification. The optical rotations were measured in MeOH at 20 °C with a Perkin-Elmer 341 polarimeter (PerkinElmer Inc., Shelton, CT, USA). The reaction progress was monitored by using analytical thin layer chromatography (TLC) on precoated silica gel GF_254_ plates (Macherey-Nagel GmbH & Co. KG, Düren, Germany). Column chromatography was performed using Merck silica gel 60 (0.015–0.040 mm). An HRMS flow injection analysis was performed with the Thermo Scientific Orbitrap Exploris 240 hybrid quadrupole-Orbitrap (Thermo Fischer Scientific, Waltham, MA, USA) mass spectrometer coupled to a Waters Acquity I-Class UPLC TM (Waters, Manchester, UK). The melting points were determined using a Kofler melting point apparatus (Nagema, Dresden, Germany) and are uncorrected. ^1^H NMR spectra were recorded at 500 MHz, while ^13^C NMR spectra were measured at 125 MHz at ambient temperature with a Bruker Avance NEO Ascend 500 spectrometer (Bruker Biospin, Karlsruhe, Germany). The chemical shifts are given, relative to tetramethylsilane (TMS) as an internal standard, in δ (ppm). The *J* values are given by Hz.

The detailed experimental process, the physical and chemical characterisation and all of the spectroscopic data (^1^H-, ^13^C-, COSY-, NOESY-, HMBC- and HSQC-NMR together with the HRMS data) of the new compounds can be found in the ESI.

### 2.3. Antiproliferative Assay

#### 2.3.1. Cell Lines and Their Maintenance

The doxorubicin-sensitive Colo205 (ATCC-CCL-222) and the doxorubicin-resistant ABCB1- and LRP-expressing Colo320/MDR-LRP (ATCC-CCL-220.1) human colon adenocarcinoma cell lines were obtained from LGC Promochem in Teddington, UK. For culturing, the RPMI 1640 medium was applied and was supplemented with a 10% heat-inactivated foetal bovine serum (FBS), 2 mM L-glutamine, 1 mM Na-pyruvate and 10 mM HEPES along with nystatin and gentamicin.

#### 2.3.2. MTT Assay

The anticancer activity of the compounds was tested in 96-well flat-bottomed microtiter plates. In this assay, the two-fold serial dilutions of the compounds (concentration range: 100–0.19 μM) were made in a 100 μL RPMI 1640 medium. Next, 1 × 10^4^ of human colonic adenocarcinoma cells (Colo205 or Colo320) in a 100 μL RPMI 1640 medium were added to each well except for the medium control wells. The starting concentration of the solvent DMSO in the plate was 2% *v*/*v*. The samples were incubated at 37 °C for 24 h. At the end of the incubation, 20 μL of an MTT (thiazolyl blue tetrazolium bromide) solution (from a 5 mg/mL stock solution) was pipetted to each well. After incubation at 37 °C for 4 h, 100 μL of a sodium dodecyl sulfate (SDS) solution (10% SDS in 0.01 M HCl) was pipetted to each well, and the plates were kept at 37 °C overnight. The growth of the cells was determined by measuring the optical density (OD) at 540 nm (refering to 630 nm) with a Multiscan EX ELISA reader (Thermo Labsystems, Cheshire, WA, USA). The inhibition of cell growth was calculated as IC_50_ values, defined as the inhibitory dose that reduces the growth of the cells exposed to the tested compounds by 50%. The IC_50_ values and the SD of the triplicate experiments were determined using GraphPad Prism software version 5.00 for Windows with a non-linear regression curve fit (GraphPad Software, San Diego, CA, USA; www.graphpad.com). Doxorubicin (from a 2 mg/mL stock solution, Teva Pharmaceuticals, Singapore) served as a positive control. The solvent (DMSO) did not interfere with the cell growth in the tested concentrations.

### 2.4. Molecular Docking Method

The crystallographic structure of the protein was retrieved from the Protein Data Bank (PDB). The 2D structures of the synthesised compounds were sketched using ChemBioDraw Ultra 11.0. The docking analysis was performed by the Accelrys Discovery Studio 3.5 software.

#### 2.4.1. Preparation of the Crystal Structures

Hydrogen atoms, added by applying several force fields (CHARMm), could lead to a steric hindrance and subsequently to a high-energy and unstable molecule. Energy minimisation—finding the most stable and lowest-energy structure and reducing H–H interactions without affecting the basic protein skeleton atoms—was performed using adopted basis minimisation. In the next step, the active site, surrounded in a 10 Å radius sphere, was determined [37].

#### 2.4.2. Docking Study (CDocker)

In the docking study utilising the CDocker method, various conformations of the compound within the protein’s active site can be generated. The outcomes can subsequently be assessed based on both the CDocker energy and the interactions between the ligand and the active site. This approach necessitates the preparation of the crystal structure (as previously mentioned) and the design of the compounds using the Accelrys Discovery Studio protocol along with the application of a force field.

Prior to commencing this study, it is crucial to underscore the validity of the employed methodology. This is achieved by comparing the conformation of the reference compound to those generated through the applied docking technique, ensuring that the Root Mean Square Deviation (RMSD) does not exceed 3 Å.

The ADMET properties of compounds **2, 12** and **13** were assessed using ADMET descriptors in Accelrys Discovery Studio 3.5. A total of six quantitative models were employed to predict the key properties of these compounds, including aqueous solubility (solubility in water at 25 °C), blood–brain barrier (BBB) permeability, inhibition of cytochrome P450 (CYP450) 2D6, human intestinal absorption (HIA) and plasma protein binding (PPB). Additionally, a dedicated ADMET model was developed to evaluate the HIA and BBB penetration for the tested compounds. This model incorporates confidence ellipses at 95% and 99% levels within the ADMET_PSA_2D and ADMET_ALogP98 frameworks. The detailed in silico ADMET properties are provided in Table S2 and are available in the Supplementary Materials.

## 3. Results and Discussion

### 3.1. Chemical Protocols

**Scheme 1:**
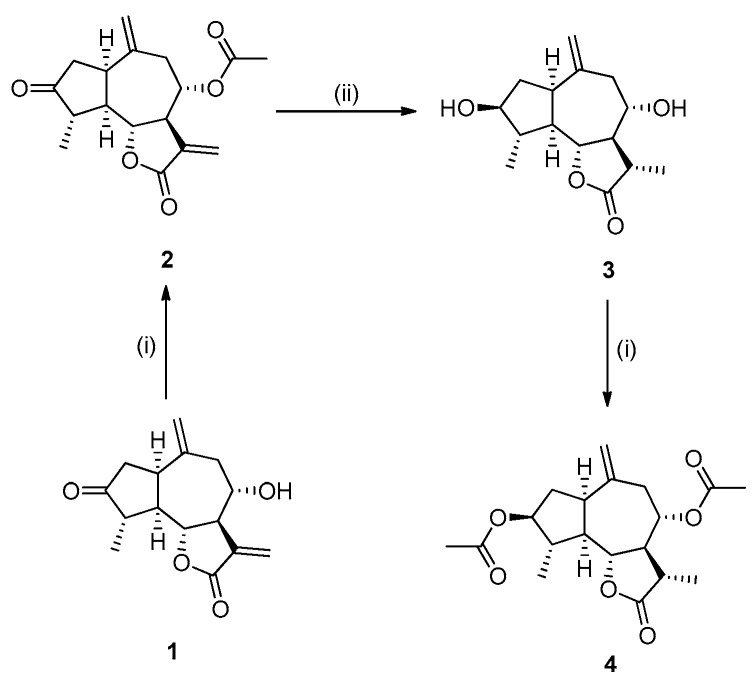
Grossheimin-based acetylated derivatives. Conditions: (i) Ac_2_O (20 eq.), dry pyridine, 25 °C, 24 h, 76% (**2**), 38% (**4**); (ii) NaBH_4_ (3 eq.), DCM:MeOH (1:1), 0 °C, 1 h, 56%.

Sesquiterpene lactone grossheimin **1** was isolated from *C. intermedia* on a gram scale. The presence of a hydroxy group in the structure allowed the preparation of its acetylated derivative, and this modification led to an improved activity of the obtained derivatives [38]. In this regard, acetylated derivative **2** was prepared under standard conditions [39]. The reduction of **2** with either NaBH_4_ or Zn (BH_4_)_2_ delivered **3** as a major product (*d.r*. = 3:1, based on NMR determination) [40]. The acetylation of **3** with the Ac_2_O/pyridine system resulted in diacetylated derivative **4** (Scheme 1).

**Scheme 2:**
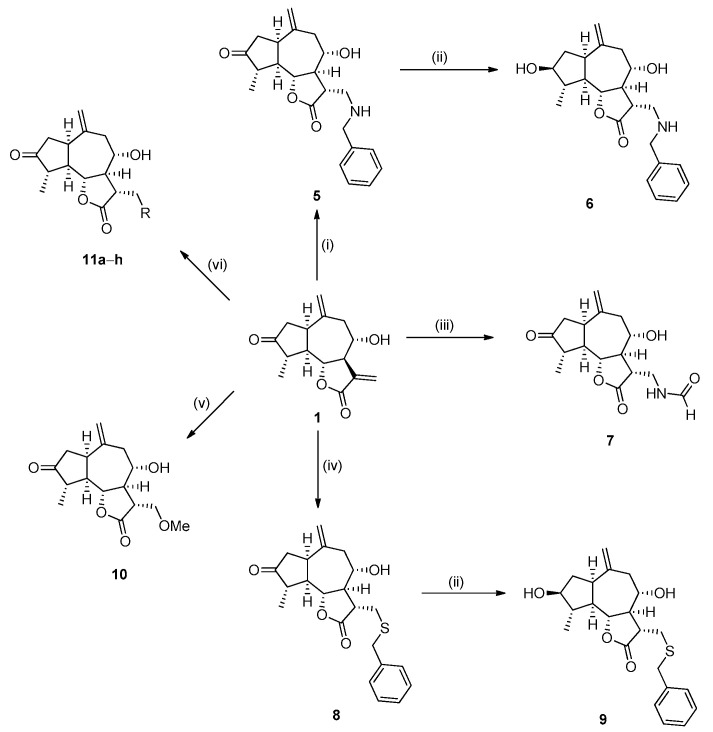
Grosshemin-based amino/thio/carbamoyl/alkoxy adducts. Conditions: (i) BnNH_2_ (2 eq.), dry EtOH, 25 °C, 24 h, 68%; (ii) NaBH_4_ (3 eq.), DCM:MeOH (1:1), 0 °C, 1 h, 43% (**6**), 47% (**9**); (iii) formamide (46 eq.), *n*-Bu_4_NBr (0.4 eq.), THF, 2 M NaOH, 70 °C, 24 h, 28%; (iv) BnSH (2 eq.), Et_3_N (1 eq.), dry EtOH, 25 °C, 24 h, 66%; (v) MeONa (3 eq.), dry MeOH, 25 °C, 48 h, 53%; (vi) azoles (2 eq.), DBU (1 eq.), dry ACN, 25 °C, 24 h, 34–67%.

In addition to the interest of the hydroxy group in the grossheimin structure, the *α*-methylene-*γ*-butyrolactone moiety provided opportunities for further functionalisation. The synthesis started with the introduction of the amino groups. The aza-Michael addition of **1** with benzylamine led to the formation of *β*-aminolactone **5** [34], which was reduced with NaBH_4_ to provide **6** with high stereoselectivity. Furthermore, the reaction of **1** with formamide in the presence of *n*-Bu_4_NBr and aqueous 2 M NaOH as the base in THF at 70 °C selectively gave compound **7** [41]. In order to obtain the thiol group at the β-position of the lactone ring of grossheimin, benzylmercaptan was used in the synthesis of the corresponding thiol **8**. As an applied amine, the amount of base like Et_3_N was a critical factor for the completion of the reaction owing to its lower reactivity [31]. When grossheimin **1** was subjected to synthetic modification using benzylmercaptan catalysed by Et_3_N, the reaction was a completely and cleanly furnished thiol adduct **8** with a satisfactory yield. The stereoselective reduction of thiol **8** gave product **9**. A simple method to obtain methoxy derivative **10** was previously described in the literature using MeONa in MeOH [41]. When this procedure was applied to **1**, compound **10** was isolated as a single product with a moderate yield (Scheme 2).

**Scheme 3:**
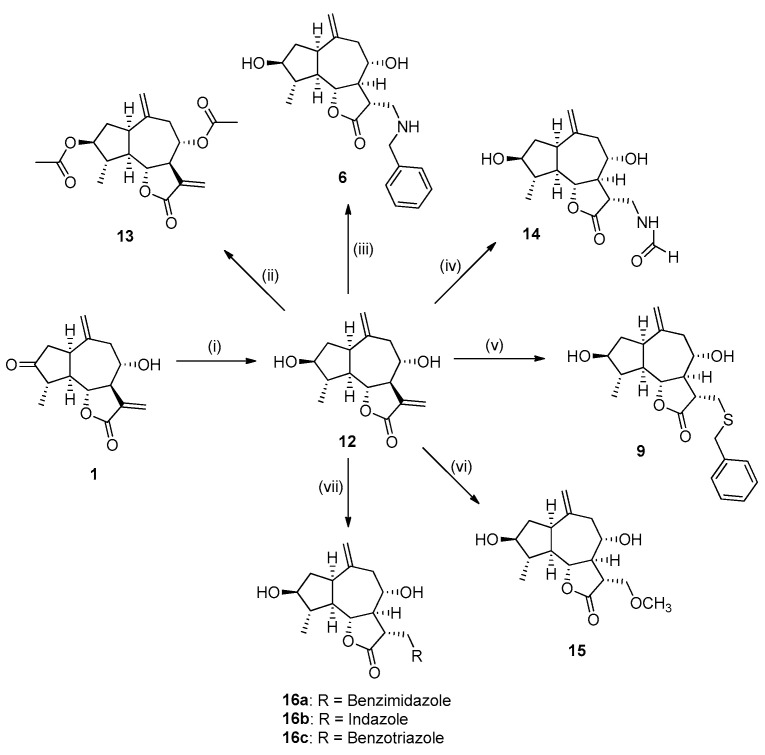
Grossheimin-derived amino/thiol/carbamoyl/alkoxy analogues. Conditions: (i) Zn(BH_4_)_2_ (2 eq.), dry ACN, 25 °C, 6 h, 81%; (ii) Ac_2_O (20 eq.), dry pyridine, 25 °C, 24 h, 49%; (iii) BnNH_2_ (2 eq.), dry EtOH, 25 °C, 24 h, 68%; (iv) formamide (46 eq.), *n*-Bu_4_NBr (0.4 eq.), THF, 2 M NaOH, 70 °C, 24 h, 31%; (v) BnSH (2 eq.), Et_3_N (1 eq.), dry EtOH, 25 °C, 24 h, 66%; (vi) MeONa (3 eq.), dry MeOH, 25 °C, 48 h, 33%; (vii) azoles (2 eq.), DBU (1 eq.), dry ACN, 25 °C, 24 h, 32–62%.

In addition to amines, azoles like imidazole, benzimidazole and others were employed as Michael donors. When the conditions used to obtain **8** were applied, product **11** was not obtained. This was probably caused by the lower nucleophilicity of *N*-heterocycles. Our attempts ultimately led to the use of DBU as a base for successfully completing the reaction [42]. When the above-mentioned synthetic modification was applied using diverse azoles, the reactions successfully furnished the expected products with acceptable yields (Scheme 3, Table 1).

In order to investigate the effect of the stereochemistry of hydroxy groups on the Michael addition at the exocyclic double bond of *α*-methylene-*γ*-butyrolactone, the reduction of ketone function in **1** was attempted to have alcohol **12**. However, the reduction of **1** with NaBH_4_ only led to the formation of **3** as a single product. Fortunately, the desired transformation was achieved by reacting **1** with Zn (BH_4_)_2_ [43,44]. The acetylation of **12** with acetic anhydride in pyridine provided diacetylated derivative **13** with a satisfactory yield. New functional groups including amino (**6**, **16**), thiol (**9**), carbamoyl (**14**) and alkoxy (**15**) were introduced at the exomethylene lactone through the Michael addition according to the same synthetic methods as described above (Scheme 3).

### 3.2. Determination of Relative Configuration

**Figure 1:**
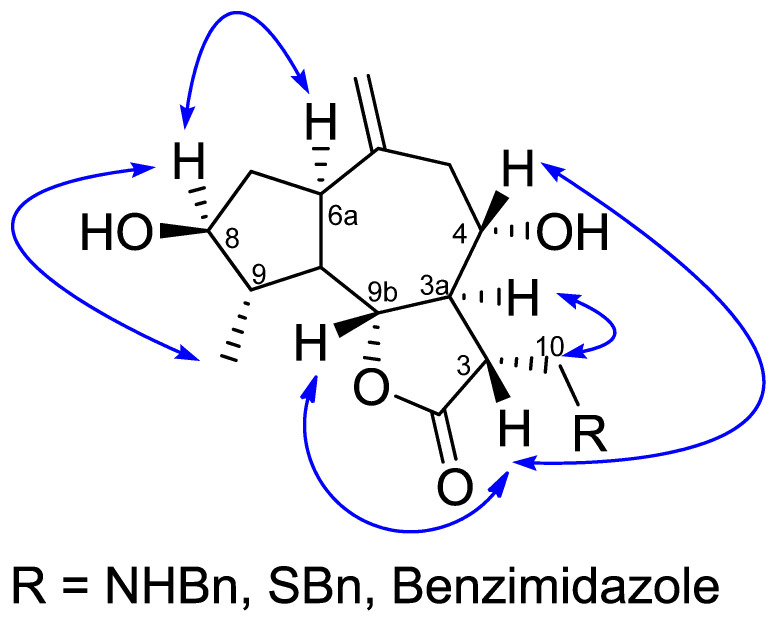
Determination of relative configuration of grossheimin-based new aminoanalogues.

Through the aza-Michael addition as well as reduction, new stereogenic centres were formed. The relative configuration of the new stereogenic centres was determined by NOESY experiments. As shown in Figure 1, clear NOE signals were observed between H-3 and H-4 together with H-9b as well as between H-10 and H-3a. Significant NOE signals were found between H-8 and H-6a together with CH_3_-9 protons. Therefore, the structure of grossheimin-derived amino analogues derived from grossheimin was determined as outlined in Figure 1.

### 3.3. Determination of Cytotoxic Effect

Since several sesquiterpene lactone-based aminoadducts exerted a significant cytotoxic action on different human cancer cell lines [34], the in vitro cytotoxic activities of the prepared grossheimin-based amino analogues were also tested on human colon adenocarcinoma lines, including Colo205 and Colo320 by MTT assay. Doxorubicin, a clinically applied anticancer agent, was used as a reference compound, and the results are summarised in Table 2.

The results indicated that grossheimin derivatives **2**, **12** and **13** exhibit considerable inhibitory properties on cancer cells. Among them, the *O*-acetyl-substituted grossheimin derivatives showed the most pronounced cytotoxic activities comparable to those of the reference agent, doxorubicin. Compound **2** with the *O*-acetyl substituent showed potent inhibitory activity against colon adenocarcinoma cancers (IC_50_ value around 9 μM) with no cytotoxicity against fibroblasts (>100 μM), while di-O-acetyl adduct **13** exhibited modest inhibitory activities with a higher IC_50_ value on the CDD-19Lu fibroblast cells (29.07 μM) than on the malignant cell lines (around 8 μM). On the other hand, none of the prepared Michael-type amino analogues exerted relevant activity (IC_50_ > 100 μM). According to the literature, α-methylene-γ-butyrolactones are excellent conjugate acceptors and act as warheads, resulting in a covalent engagement with the target protein (e.g., the reaction with L-cysteinyl residues in proteins/enzymes) [45,46]. A modification in the α-methylene-γ-lactone motif led to the loss of function as Michael acceptors in reactions with thiol groups of bionucleophiles, whereas acetylation improved lipophilicity, enhancing the biological activity of the parent molecule.

### 3.4. Molecular Docking

**Figure 2:**
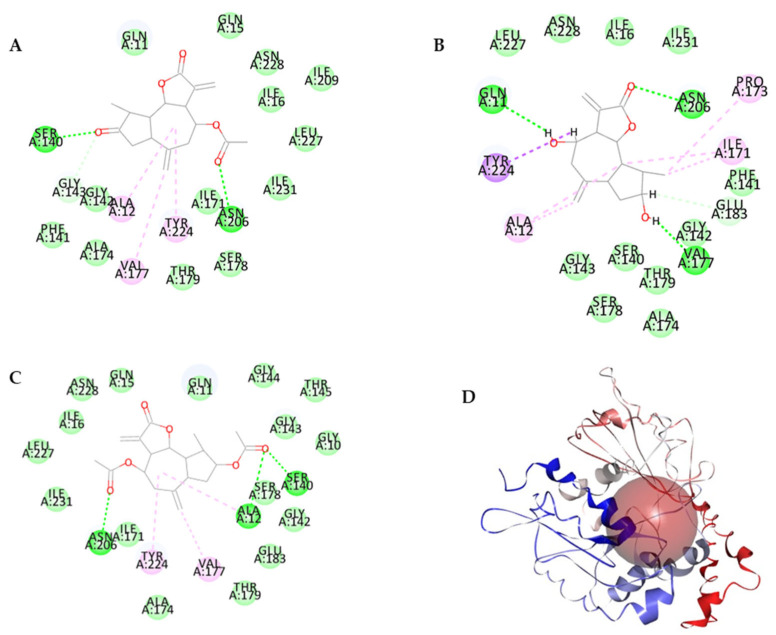
A 2D diagram of the interactions between compound **2** (**A**), compound **12** (**B**) and compound **13** (**C**) with the tubulin binding site and the crystal structure of tubulin (**D**).

A docking study was conducted to predict the potential target of the most biologically active compounds based on the antiproliferative result (compounds **2**, **12** and **13**). To achieve this purpose, a group of protein targets, such as Aurora A (PDB code: 4DEE, resolution: 2.30 Å), ALK (PDB code: 4ZJI, resolution: 1.99 Å), Pim-1 (PDB code: 4ALW, resolution: 1.92 Å) and tubulin (PDB code: 1SAO, resolution: 3.58 Å), were used as templates for the docking study using the CDocker method [47]. The results indicated that all of the tested compounds are likely to exhibit an affinity toward the active site of tubulin by forming strong bonds with several amino acids (Figure 2) [48]. The CDocker interaction energy of the tested compounds is shown in Table S1 in the Supplementary Materials. The docking results of the studied compounds show the key role of the hydroxy and acetyl groups in forming hydrogen bonding and ionic interactions with the key amino acids (Figure 2). The in silico ADMET study showed that all of the tested compounds might have good absorption properties with a low ability to penetrate the BBB (Table S2 and Figure S1 in the Supplementary Materials).

Tubulin plays a crucial role in cell division, intracellular transport and cytoskeletal stability. Cancer cells exploit tubulin dynamics to maintain uncontrolled proliferation and resistance to apoptosis. Tubulin inhibitors arrest mitosis, leading to tumor cell death [49]. Our results show that compounds **2** and **13** bind to the colchicine site of tubulin, suggesting a mechanism of microtubule destabilisation, which may offer a novel approach for colorectal cancer therapy. This study highlights compounds **2** and **13** as promising tubulin inhibitors, with interactions involving Thr179, Glu183 and Lys352. Their strong binding suggests the potential for further development in colorectal cancer therapy.

## 4. Conclusions

In conclusion, starting from isolated grossheimin, a new family of grossheimin-derived chiral amino analogues was prepared via stereoselective transformations. All of the compounds were screened for their cytotoxic effects on human colon adenocarcinoma lines. Only the acetylated compounds exhibited significant cytotoxicity within the series of derivatives as compared to the precursor grossheimin. Among these *O*-acetyl derivatives, compound **2** exerted outstanding activities against the malignant cells with no action on fibroblasts, indicating considerable cancer selectivity. The structure–activity relationship clearly indicated that functionalisation at the hydroxy group led to an enhanced effect against all of the studied cell lines, whereas the modification at the *α*-methylene-*γ*-butyrolactone moiety resulted in a total loss of activity.

The molecular docking studies proved that derivatives **2**, **12** and **13** could foster a potent affinity by forming significant hydrogen bonding and ionic interactions with the key amino acids with tubulin (PDB code: 1SAO, resolution: 3.58 Å). Thus, these novel leads from grossheimin can be further developed into potential chemotherapeutic agents in colorectal cancer therapy.

In the next stage of our project, esterfication with heterocyclic carboxylic or halogenated acids as well as halogenation could be achieved and their cytotoxic activities on various human cancer cell lines investigated. Additionally, for the optimised compounds, a tubulin inhibitory activity and molecular dynamics study will also be performed to obtain insight into the dynamics of ligand interaction.

## Figures and Tables

**Table 1:** Grossheimin-based azoles **11a–h**.

Entry	Compound	R	Yield (%)
**1**	**11a**	Imidazole	53
**2**	**11b**	Pyrazole	42
**3**	**11c**	1,2,4-Triazole	67
**4**	**11d**	1,2,3-Triazole	66
**5**	**11e**	Indole	35
**6**	**11f**	Benzimidazole	50
**7**	**11g**	Indazole	64
**8**	**11h**	Benzotriazole	34

**Table 2:** The cytotoxic effects of grossheimin-based aminoanalogues on colon adenocarcinoma cell lines and CDD-19Lu fibroblasts. Doxorubicin (DOX) was applied as a positive control.

Compound	IC_50_ (μM)		
	Colo205	Colo320	CDD-19Lu
**1**	23.33 ± 2.05	27.89 ± 2.00	>100
**2**	8.21 ± 0.51	9.44 ± 0.41	>100
**12**	63.79 ± 1.32	53.65 ± 1.60	>100
**13**	8.21 ± 0.51	6.98 ± 0.29	29.07 ± 0.46
**DOX**	3.21 ± 0.03	4.36 ± 0.08	>8.62

## Data Availability

Data are contained within the article.

## Supplementary Materials

The following supporting information can be downloaded at: https://www.mdpi.com/article/10.3390/biom15040578/s1, Figure S1: Plot of Polar Surface Area (PSA) vs. LogP for a standard and test set; Table S1: Cytotoxic effects of grossheimin-based aminoanalogues on colon adenocarcinoma cell lines and CDD-19Lu fibroblasts.; Table S2: CDocker energy values for compounds **2**, **12** and **13**; Table S3: In-silico ADMET study.
