# Supplementary material for: Synthesis and Antiproliferative Effects of Grossheimin-Derived Aminoanalogues
Source: Biomolecules. 2025 Apr 14;15(4):578. doi: 10.3390/biom15040578 (PMC12024577; doi:10.3390/biom15040578)

Supporting information for

Synthesis and Antiproliferative Effects of Grossheimin-Derived Aminoanalogues

Meruyert Ashimbayeva [1], Zsolt Szakonyi [1,*], Sergazy M. Adekenov [2], Nikoletta Szemerédi [3], Gabriella Spengler [3] and Tam Minh Le [1,4,*]

[1] Institute of Pharmaceutical Chemistry, University of Szeged, Eötvös u. 6, H-6720 Szeged, Hungary; mikosha.3791@mail.ru
[2] JSC Research and Production Center "Phytochemistry", Karaganda 100009, Kazakhstan; arglabin@phyto.kz
[3] Department of Medical Microbiology, Albert Szent-Györgyi Health Center and Albert Szent-Györgyi Medical School, University of Szeged, Semmelweis utca 6, H-6725 Szeged, Hungary; szemeredi.nikoletta@med.u-szeged.hu (N.S.); spengler.gabriella@med.u-szeged.hu (G.S.)
[4] HUN-REN–SZTE Stereochemistry Research Group, University of Szeged, Eötvös u. 6, H-6720 Szeged, Hungary
* Correspondence: szakonyi.zsolt@szte.hu (Z.S.); leminhtam1411@gmail.com (T.M.L.)

Contents

**1.**	**General methods**	3
**2.**	**Experimental section and compound characterisation**	4 – 9
**3.**	**Cytotoxic effects of grossheimin-based aminoanalogues**	10
**3.**	**^{1}H-, ^{13}C- JMOD NMR spectra of new compounds**	11 – 70
**4.**	**Docking study**	71

1. General methods

Commercially available solvents were used as obtained from suppliers (Molar Chemicals Ltd, Halásztelek, Hungary; Merck Ltd., Budapest, Hungary and VWR International Ltd., Debrecen, Hungary), while applied solvents were dried according to standard procedures. Optical rotations were measured in MeOH at 20 °C with a Perkin-Elmer 341 polarimeter (PerkinElmer Inc., Shelton, CT, USA). Chromatographic separations and monitoring of reactions were carried out on Merck Kieselgel 60 (Merck Ltd., Budapest, Hungary). HRMS flow injection analysis was performed with Thermo Scintific Thermo Scientific Orbitrap Exploris 240 hybrid quadrupole-Orbitrap (Thermo Fischer Scientific, Waltham, MA, USA) mass spectrometer coupled to a Waters Acquity I-Class UPLC TM (Waters, Manchester, UK). Melting points were determined on a Kofler apparatus (Nagema, Dresden, Germany) and are uncorrected. ^{1}H- and ^{13}C JMOD NMR were recorded on Brucker Avance DRX 500 spectrometer [500 MHz (^{1}H) and 125 MHz (^{13}C), $\delta = 0$ (TMS)]. Chemical shifts are expressed in ppm (δ) relative to TMS as the internal reference. *J* values are given by Hz.

Grossheimin was isolated from aerial parts of *C. intermedia* according to the literature methods. All physical and spectroscopic properties of grossheimin were similar to those described in the literature.[1]

1. Adekenov, S.M.; Shaimerdenova, Z.R.; Adekenova, K.S.; Kishkentayeva, A.S. Synthesis and Biological Evaluation of New Derivatives of Grossheimin. *Fitoterapia* **2022**, *158*, 105154, doi:10.1016/j.fitote.2022.105154

2. Experimental section and compound characterisation

(3aR,4S,6aR,8R,9S,9aR,9bR)-4,8-dihydroxy-9-methyl-3,6-dimethylenedecahydroazuleno[4,5-b]furan-2(9bH)-one (**12**): In a round-bottomed flask equipped with a magnetic stirrer bar, a solution of grossheimin **1** (0.11 mmol) was prepared in CH_3CN (2.0 mL). To this solution, $Zn(BH_4)_2/2NaCl$ (0.22 mmol) was added and the mixture was stirred at room temperature for 6 hours. The reaction was monitored by TLC (eluent; *n*-hexane:EtOAc = 1:4). After completion of the reaction, brine (5 mL) was added to the reaction mixture and stirred for 5 min. The mixture was extracted with EtOAc (3 x 10.0 mL) and dried over anhydrous Na_2SO_4. Evaporation of the solvent, followed by column chromatography of the resulting crude material over silica gel (eluent; *n*-hexane:EtOAc = 1:4) afforded the expected product. Yield: 81%; oily compound; $[\alpha]_D^{20}$ = +53.5 (c 0.12, MeOH): ^{1}H NMR (500 MHz, $CDCl_3$): δ = 6.25 (d, *J* = 3.3 Hz, 1H), 6.17 (d, *J* = 2.9 Hz, 1H), 5.06 (s, 1H), 4.99 (s, 1H), 4.07 (t, *J* = 9.6 Hz, 1H), 3.86 (s, 1H), 3.78 (q, *J* = 7.5 Hz 1H), 2.93-2.86 (m, 1H), 2.78-2.69 (m, 2H), 2.22-2.10 (m, 2H), 2.01-1.92 (m, 2H), 1.80-1.62 (m, 2H), 1.19 (d, *J* = 6.3 Hz, 3H); ^{13}C NMR (125 MHz, $CDCl_3$): δ = 143.0, 138.3, 123.4, 115.9, 81.1, 78.7, 73.2, 53.4, 52.1, 47.1, 45.2, 43.5, 38.9, 18.5; HR-MS (ESI): *m/z* calcd for $C_{15}H_{21}O_4$ $[M+H]^+$: 265.1439; found: 265.1431.

2.1. General procedure for reduction process

Compound **2**, **5** or **8** (0.11 mmol) was dissolved in 2.0 mL CH_2Cl_2:MeOH (1:1). $NaBH_4$ (0.33 mmol) was lately added to the solution. After stirred for 1 hour at 0 °C, brine (10.0 mL) was added and the solution was extracted with CH_2Cl_2 (3 x 10.0 mL). The organic phase was dried over Na_2SO_4, filtered then evaporated. The crude product was purified by column chromatography on silica gel using the appropriate solvent as eluent.

(3S,3aR,4S,6aR,8R,9S,9aR,9bR)-4,8-Dihydroxy-3,9-dimethyl-6-methylenedecahydroazuleno[4,5-b]furan-2(9bH)-one (**3**): Eluted with *n*-hexane:EtOAc = 1:4. Yield: 56%; white crystals; m.p.: 164.2-166.3 °C; $[\alpha]_D^{20}$ = +32.2 (c 0.13, MeOH); ^{1}H NMR (500 MHz, $CDCl_3$): δ = 5.01 (d, *J* = 15.2 Hz, 2H), 3.94 (t, *J* = 9.93 Hz, 1H), 3.75-3.63 (m, 2H), 2.81 (q, *J* = 9.94 Hz, 1H), 2.73 (dd, *J* = 4.2, 12.4 Hz, 1H), 2.56-2.47 (m, 1H), 2.17-2.06 (m, 2H), 1.95-1.83 (m, 3H), 1.76-1.62 (m, 3H), 1.4 (d, *J* = 6.4 Hz, 3H); ^{13}C NMR (125 MHz, $CDCl_3$): δ = 178.7, 143.4, 115.2, 81.2, 78.3, 76.0, 58.3, 51.3, 47.3, 47.2, 42.5, 42.2, 38.4, 18.2, 16.0; HR-MS (ESI): *m/z* calcd for $C_{15}H_{23}O_4$ $[M+H]^+$: 267.1596; found: 267.1587.

3R,3aR,4S,6aR,8R,9S,9aR,9bR)-3-((Benzylamino)methyl)-4,8-dihydroxy-9-methyl-6-methylenedecahydroazuleno[4,5-b]furan-2(9bH)-one (**6**): Eluted with $CHCl_3$:MeOH = 19:1. Yield: 43%; white crystals; m.p.: 189.6-190.2 °C; $[\alpha]_D^{20}$ = +45.3 (c 0.18, MeOH); ^{1}H NMR (500 MHz, $CDCl_3$): δ = 7.38-7.28 (m, 5H), 5.00 (d, *J* = 14.2 Hz, 2H), 4.00 (t, *J* = 10.1 Hz, 1H), 3.82 (q, *J* = 12.7 Hz, 2H), 3.76-3.68 (m, 1H), 3.57-3.46 (m, 2H), 2.86-2.78 (m, 2H), 2.64 (t, *J* = 11.2 Hz, 1H), 2.47 (td, *J* = 2.0, 11.3 Hz,1H), 2.15-1.92 (m, 5H), 1.87 (q, *J* = 7.4 Hz, 1H), 1.71 (q, *J* = 11.7 Hz, 1H), 1.18 (d, *J* = 6.6 Hz, 3H); ^{13}C NMR (125 MHz, $CDCl_3$): δ = 175.2, 143.8, 137.7, 129.0, 128.6, 128.0, 114.7, 81.43, 78.29, 73.83, 60.5, 53.8, 50.4, 49.7, 48.2, 47.3, 44.9, 42.4, 38.3, 18.6; HR-MS (ESI): *m/z* calcd for $C_{22}H_{30}NO_4$ $[M+H]^+$: 372.2174; found: 372.2162.

(3S,3aR,4S,6aR,8R,9S,9aR,9bR)-3-((Benzylthio)methyl)-4,8-dihydroxy-9-methyl-6-methylenedecahydroazuleno[4,5-b]furan-2(9bH)-one (**9**): Eluted with $CHCl_3$:MeOH = 19:1. Yield: 47%; oily compound; $[\alpha]_D^{20}$ = +17.7 (c 0.15, MeOH); ^{1}H NMR (500 MHz, $CDCl_3$): δ = 7.35-7.30 (m, 3H), 7.26-7.24 (m, 2H), 5.00 (d, *J* = 13.8 Hz, 2H), 3.94 (t, *J* = 4.0 Hz, 1H), 3.79 (s, 2H), 3.73 (q, *J* = 8.4 Hz, 1H), 3.60-3.52 (m, 1H), 3.10 (dd, *J* = 3.9, 13.6 Hz, 1H), 2.92 (dd, *J* = 5.0, 13.6 Hz 1H), 2.80 (q, *J* = 9.8 Hz, 1H), 2.73-2.64 (m, 2H), 2.14 (quin, *J* = 6.5 Hz, 1H), 2.03 (dd, *J* = 9.8, 12.3 Hz, 3H), 1.96-1.89 (m, 1H), 1.88-1.81 (m, 1H) 1.74-1.60 (m, 2H), 1.18 (d, *J* = 6.5 Hz, 3H); ^{13}C NMR (125 MHz, $CDCl_3$): δ = 176.2, 143.3, 138.2, 129.2, 128.7, 127.4, 115.3, 81.4, 78.4, 75.2, 54.7, 51.1, 47.8, 47.2, 46.3, 42.6, 38.4, 37.6, 31.3, 18.2; HR-MS (ESI): *m/z* calcd for $C_{22}H_{29}O_4S$ $[M+H]^+$: 389.1786; found: 389.1774.

2.2. General procedure for acetylation process

To the solution of **1**, **3** or **12** (0.114 mmol) in dry pyridine (1.0 mL), acetic anhydride (2.28 mmol) was added. After stirring 24 hours at room temperature (monitored by TLC), the reaction was quenched by pouring into a saturated copper sulfate solution, followed by extraction with EtOAc (3 x 25.0 mL). The organic phase was washed with water (3 x 10.0 mL), dried over anhydrous Na_2SO_4, and evaporated to dryness. The crude product was purified to provide the expected product.

(3aR,4S,6aR,9S,9aR,9bR)-9-Methyl-3,6-dimethylene-2,8-dioxododecahydroazuleno[4,5-b]furan-4-yl acetate **(2)**: Recrystallized in Et_2O. Yield: 76%; white crystals; m.p.: 165.5-166.8 °C; $[\alpha]_D^{20}$ = +104.3 (c 0.18, MeOH): ^{1}H NMR (500 MHz, $CDCl_3$): δ = 6.34 (d, *J* = 3.2 Hz, 1H), 5.82 (d, *J* = 2.7 Hz, 1H), 5.14 (s, 1H), 4.99-4.91 (m, 1H), 4.87 (s, 1H), 4.06 (t, *J* = 9.1 Hz, 1H), 3.34-3.27 (m, 1H), 3.19-3.13 (m, 1H), 2.98 (dd, *J* = 5.7, 13.0 Hz 1H), 2.59-2.46 (m, 2H), 2.39-2.27 (m, 2H), 2.23 (dd, *J* = 8.8, 13.3 Hz, 1H), 2.15 (s, 3H), 1.27 (d, *J* = 6.7 Hz, 3H); ^{13}C NMR (125 MHz, $CDCl_3$): δ = 218.5, 169.8, 169.2, 142.4, 135.9, 125.0, 116.9, 82.4, 74.4, 51.2, 47.0, 46.5, 43.5, 43.2, 40.2, 21.2, 14.9; HR-MS (ESI): *m/z* calcd for $C_{17}H_{21}O_5$ $[M+H]^+$: 305.1389; found: 305.1382.

(3S,3aR,4S,6aR,8S,9S,9aR,9bR)-3,9-dimethyl-6-methylene-2-oxododecahydroazuleno[4,5-b]furan-4,8-diyl diacetate **(4)**: Recrystallized in Et_2O. Yield: 38%; oily compound; $[\alpha]_D^{20}$ = +39.0 (c 0.19, MeOH); ^{1}H NMR (500 MHz, $CDCl_3$): δ = 5.11 (s, 1H), 5.04 (s, 1H), 4.82 (td, *J* = 4.4, 10.1 Hz, 1H), 4.68 (td, *J* = 6.5, 8.7 Hz, 1H), 3.95 (t, *J* = 10.2 Hz, 1H), 2.87 (q, *J* = 9.7 Hz, 1H), 2.79 (dd, *J* = 4.3, 12.4 Hz, 1H), 2.50-2.42 (m, 1H), 2.33 (q, *J* = 6.8 Hz, 1H), 2.09 (s, 3H), 2.07 (s, 3H), 2.05-2.17 (m, 3H), 1.98-1.92 (m, 1H), 1.73-1.65 (m, 1H), 1.28 (d, *J* = 7.0 Hz, 3H), 1.25 (s, 1H), 1.15 (d, *J* = 6.7 Hz, 3H); ^{13}C NMR (125 MHz, $CDCl_3$): δ = 177.8, 171.2, 170.1, 142.1, 116.4, 80.9, 79.5, 76.5, 55.6, 50.6, 43.9, 42.9, 42.8. 41.5, 35.3, 21.3, 18.2, 15.6; HR-MS (ESI): *m/z* calcd for $C_{19}H_{27}O_6$ $[M+H]^+$: 351.1807; found: 351.1798.

(3aR,4S,6aR,8S,9S,9aR,9bR)-9-Methyl-3,6-dimethylene-2-oxododecahydroazuleno[4,5-b]furan-4,8-diyl diacetate (**13**): Purified on silica gel using *n*-hexane:EtOAc = 2:1 as eluent. Yield: 49%; oily compound; $[\alpha]_D^{20}$ = +56.0 (c 0.17, MeOH); ^{1}H NMR (500 MHz, $CDCl_3$): δ = 6.23 (d, *J* = 3.3 Hz, 1H), 5.63 (d, *J* = 2.9 Hz, 1H), 5.07 (d, *J* = 2.5 Hz, 2H), 4.94-4.88 (m, 1H), 4.73 (q, *J* = 7.9 Hz, 1H), 4.05 (dd, *J* = 9.3, 10.7 Hz 1H), 3.05-2.98 (m, 1H), 2.95 (q, *J* = 9.7 Hz, 1H), 2.81 (dd, *J* = 4.7, 13.2 Hz 1H), 2.31 (quin, *J* = 6.9 Hz 1H), 2.23 (q, *J* = 6.9 Hz, 1H), 2.17 (t, *J* = 7.8, 1H), 2.14 (s, 3H), 2.06 (s, 3H), 2.03-1.96 (m, 1H), 1.73-1.65 (m, 1H), 1.17 (d, *J* = 6.8, 3H); ^{13}C NMR (125 MHz, $CDCl_3$): δ = 171.1, 170.1, 169.3, 141.5, 137.4, 122.7, 117.3, 80.6, 79.8, 74.5, 51.6, 49.9, 43.7, 43.6, 49.7, 35.7, 21.3, 18.4; HR-MS (ESI): *m/z* calcd for $C_{19}H_{25}O_6$ $[M+H]^+$: 349.1651; found: 349.1643.

2.3. General procedure for preparation of carbamoyl derivatives

Compound **1** or **12** (0.189 mmol) was dissolved in THF (1.0 mL). Formamide (8.7 mmol), *n*-Bu_4NBr (0.075mmol) and 2 M NaOH (1.0 mL) were added. The reaction mixture was stirred at 70 °C for 24 hours. After completion, the reaction was quenched with 10.0 mL of Sorenson buffer solution (pH = 7). Subsequently, 20 mL of brine was added, and the product was extracted using ethyl acetate (8 x 20.0 mL). The combined organic extracts were dried over anhydrous Na_2SO_4, and the solvent was removed under reduced pressure. The crude products were purified by column chromatography on silica gel, eluted by $CHCl_3$: MeOH = 19:1 to give compound **7** or **14**, respectively.

(N-(((3R,3aR,4S,6aR,9S,9aR,9bR)-4-hydroxy-9-methyl-6-methylene-2,8-dioxododecahydroazuleno[4,5-b]furan-3-yl)methyl)formamide **(7)**: Yield: 28%; oily compound; $[\alpha]_D^{20}$ = +49.6 (c 0.10, MeOH); ^{1}H NMR (500 MHz, $CDCl_3$): δ = 8.23 (s, 1H), 6.50 (s, 1H), 5.07 (s, 1H), 4.74 (s, 1H), 4.10-3.95 (m, 2H), 3.70-3.62 (m, 2H), 3.09 (td, *J* = 3.9, 7.7 Hz, 1H), 2.91 (dd, *J* = 12.6, 5.4 Hz, 1H), 2.80 (dd, *J* = 11.4, 2.3 Hz, 1H), 2.54-2.45 (m, 2H), 2.37 (q, *J* = 10.1 Hz, 3H), 2.29-2.13 (m, 3H), 1.21 (d, *J* = 6.7 Hz, 3H); ^{13}C NMR (125 MHz, $CDCl_3$): δ = 218.6, 176.5, 163.4, 144.3, 114.9, 84.4, 75.7, 51.4, 49.7, 49.0, 47.7, 47.4, 43.8, 39.6, 38.0, 29.8, 14.3; HR-MS (ESI): *m/z* calcd for $C_{16}H_{22}NO_5$ $[M+H]^+$: 308.1497; found: 308.1492.

N-(((3R,3aR,4S,6aR,8S,9S,9aR,9bR)-4,8-dihydroxy-9-methyl-6-methylene-2-oxododecahydroazuleno[4,5-b]furan-3-yl)methyl)formamide **(14)**: Yield: 31%; white crystals; m.p.:

108.8-109.2 °C;; $[\alpha]_D^{20}$ = +26.7 (c 0.14, MeOH); ^{1}H NMR (500 MHz, $CDCl_3$): δ = 8.21 (s, 1H), 6.28 (s, 1H), 5.02 (d, *J* = 11.3 Hz, 2H), 4.33 (d, *J* = 9.4 Hz, 1H), 4.18-4.09 (m, 1H), 3.98 (t, *J* = 9.8 Hz, 1H), 3.73-3.66 (m, 1H), 3.63-3.53 (m, 2H), 2.87-2.70 (m, 3H), 2.14 (quin, *J* = 6.4 Hz, 1H), 2.05-1.96 (m, 2H), 1.93-1.78 (m, 2H), 1.76-1.66 (m, 2H), 1.18 (d, *J* = 6.4 Hz, 3H); ^{13}C NMR (125 MHz, $CDCl_3$): δ = 163.29, 143.67, 115.03, 82.52, 78.20, 75.96, 53.23, 51.01, 48.58, 48.07, 47.24, 42.16, 38.34, 37,74, 18.23; HR-MS (ESI): *m/z* calcd for $C_{16}H_{24}NO_5$[M+H]$^+$: 310.1654; found: 310.1643.

2.4. General procedure for the preparation of methoxy derivatives

Compound **1** or **12** (0.12 mmol) was dissolved in dry MeOH (5.0 mL), followed by the addition of sodium methoxide (MeONa) (0.36 mmol). The mixture was stirred at room temperature for 48 hours. After this period, the MeOH was evaporated under vacuum nearly to dryness. The residue was then diluted with EtOAc (20 mL) and brine (20 mL) was added. The product was extracted with EtOAc (3 x 20 mL), and the combined organic layers were dried over anhydrous Na_2SO_4. The solvent was evaporated under vacuum. The resulting residue was purified *via* column chromatography using a 1:4 mixture of *n*-hexane:EtOAc as the eluent, yielding compound **10** or **15** as a product.

(3R,3aR,4S,6aR,9S,9aR,9bR)-4-hydroxy-3-(methoxymethyl)-9-methyl-6-methyleneoctahydroazuleno[4,5-b]furan-2,8(3H,9bH)-dione **(10)**: Yield: 53%; oily compound; $[\alpha]_D^{20}$ = +84.6 (c 0.10, MeOH); ^{1}H NMR (500 MHz, $CDCl_3$): δ = 5.08 (s, 1H), 4.79 (s, 1H), 4.27 (s, 1H), 4.02 (quin, *J* = 3.6 Hz, 2H), 3.67 (q, *J* = 9.4 Hz, 1H), 3.53 (t, *J* = 9.2 Hz, 1H), 3.47 (t, *J* = 11.0 Hz, 3H), 3.14 (q, *J* = 7.9 Hz, 1H), 2.90-2.79 (m, 2H), 2.57-2.44 (m, 3H), 2.34-2.19 (m, 3H), 1.23 (d, *J* = 6.8 Hz, 3H); ^{13}C NMR (125 MHz, $CDCl_3$): δ = 213.3, 144.1, 115.1, 83.1, 74.3, 72.1, 59.4, 53.8, 51.3, 47.7, 47.4, 47.2, 43.4, 40.0, 14.8; HR-MS (ESI): *m/z* calcd for $C_{16}H_{23}O_5$ [M+H]$^+$: 295.1545; found: 295.1538.

(3R,3aR,4S,6aR,8S,9S,9aR,9bR)-4,8-dihydroxy-3-(methoxymethyl)-9-methyl-6-methylenedecahydroazuleno[4,5-b]furan-2(9bH)-one **(15)**: Yield: 33%; white crystals; m.p.: 97.6-98.7 °C; $[\alpha]_D^{20}$ = +23.4 (c 0.10, MeOH); ^{1}H NMR (500 MHz, $CDCl_3$): δ = 5.02 (d, *J* = 6.2 Hz, 2H), 4.56 (s, 1H), 4.06-3.98 (m, 2H), 3.76-3.69 (m, 1H), 3.57 (td, *J* = 4.2, 9.3 Hz, 1H), 3.48 (t, *J* = 9.6 Hz, 1H), 3.45 (s, 3H), 2.85-2.72 (m, 3H), 2.16-2.02 (m, 3H), 1.96 (q, *J* = 10.1 Hz, 1H), 1.88 (sex, *J* = 6.7 Hz, 1H), 1.72 (q, *J* = 11.3 Hz, 1H), 1.19 (d, *J* = 6.6 Hz, 3H); ^{13}C NMR (125 MHz, $CDCl_3$): δ = 174.2, 143.5, 115.0, 81.5, 78.3, 74.1, 72.3, 59.4, 58.7, 50.7, 48.3, 47.3, 44.6, 42.6, 38.4, 18.5; HR-MS (ESI): *m/z* calcd for $C_{16}H_{25}O_5$ [M+H]$^+$: 297.1702; found: 297.1696.

2.5. General procedure for grossheimin-based azoles

Compound **1** or **12** (0.11 mmol) was dissolved in dry CH_3CN (2.0 mL), followed by the addition of the appropriate azoles (0.22 mmol) and DBU (0.11 mmol). The mixture was stirred for 24 hours at room temperature. After the reaction was complete, the mixture was allowed to cool, and the solvent was evaporated under vacuum using a rotary evaporator. The resulting residue was purified by column chromatography on silica gel, employing a $CHCl_3$:MeOH = 19:1 as the eluent, to obtain the pure product.

(3R,3aR,4S,6aR,9S,9aR,9bR)-3-((1H-imidazol-1-yl)methyl)-4-hydroxy-9-methyl-6-methyleneoctahydroazuleno[4,5-b]furan-2,8(3H,9bH)-dione **(11a)**: Prepared with **1** and imidazole. Yield: 53%; white crystals; m.p.: 200.3-201.6 °C; $[\alpha]_D^{20}$ = +130.4 (c 0.18, MeOH); ^{1}H NMR (500 MHz, $CDCl_3$): δ = 7.58 (s, 1H), 7.05 (d, *J* = 10.1 Hz, 2H), 5.05 (s, 1H), 4.73 (s, 1H), 4.67-4.61 (m, 1H), 4.54 (dd, *J* = 5.0, 14.1 Hz, 1H), 3.96 (d, *J* = 9.3 Hz, 1H), 3.82 (td, *J* = 5.6, 10.0 Hz, 1H), 2.95 (td, *J* = 2.7, 8.4 Hz, 1H), 2.89-2.79 (m, 2H), 2.53-2.39 (m, 2H), 2.29-2.15 (m, 2H), 2.05-1.95 (m, 2H), 1.18 (d, *J* = 7.1 Hz, 3H); ^{13}C NMR (125 MHz, $CDCl_3$): δ = 218.6, 175.8, 143.7, 129.8, 120.2, 115.2, 83.7, 75.8, 51.1, 49.5, 48.6, 47.4, 47.3, 45.4, 43.7, 39.5, 14.3; HR-MS (ESI): *m/z* calcd for $C_{18}H_{23}N_2O_4$ [M+H]$^+$: 331.1657; found: 331.1644.

(3R,3aR,4S,6aR,9S,9aR,9bR)-3-((1H-pyrazol-1-yl)methyl)-4-hydroxy-9-methyl-6-methyleneoctahydroazuleno[4,5-b]furan-2,8(3H,9bH)-dione **(11b)**: Prepared with **1** and pyrazole.

Yield: 42%; white crystals; m.p.: 75.9-77.1 °C; $[\alpha]_D^{20}$ = +101.3 (c 0.19, MeOH); ^{1}H NMR (500 MHz, $CDCl_3$): δ = 7.55 (s, 1H), 7.47 (d, *J* = 1.8 Hz, 1H), 6.29 (s, 1H), 5.06 (s, 1H), 4.97 (d, *J* = 8.7 Hz, 1H), 4.80 (dd, *J* = 4.9, 14.2 Hz, 1H), 4.77-4.71 (m, 2H), 3.96 (t, *J* = 9.4 Hz, 1H), 3.77-3.67 (m, 1H), 2.97-2.88 (m, 3H), 2.51-2.40 (m, 2H), 2.24 (t, *J* = 8.8 Hz, 1H), 2.14 (t, *J* = 11.1 Hz, 1H), 2.05-1.94 (m, 2H), 1.68 (s, 1H), 1.17 (d, *J* = 7.0 Hz, 3H); ^{13}C NMR (125 MHz, $CDCl_3$): δ = 218.6, 175.5, 144.2, 139.8, 131.3, 114.9, 106.6, 83.6, 75.4, 51.2, 49.8, 49.7, 49.4, 48.3, 47.3, 43.6, 39.7, 14.3; HR-MS (ESI): *m/z* calcd for $C_{18}H_{23}N_2O_4$ $[M+H]^+$: 331.1657; found: 331.1646.

(3R,3aR,4S,6aR,9S,9aR,9bR)-3-((1H-1,2,4-triazol-1-yl)methyl)-4-hydroxy-9-methyl-6-methyleneoctahydroazuleno[4,5-b]furan-2,8(3H,9bH)-dione **(11c)**: Prepared with **1** and 1,2,4-triazole Yield: 67%; white crystals; m.p.: 170.2-172.5 °C; $[\alpha]_D^{20}$ = +86.5 (c 0.17, MeOH); ^{1}H NMR (500 MHz, $CDCl_3$): δ = 8.17 (s, 1H), 8.00 (s, 1H), 5.08 (s, 1H), 4.86-4.77 (m, 2H), 4.76 (s, 1H), 3.98 (t, *J* = 9.3 Hz, 1H), 3.81-3.73 (m, 1H), 3.32 (d, *J* = 7.4 Hz, 1H), 3.02-2.96 (m, 1H), 2.90 (dd, *J* = 5.4, 12.5 Hz, 1H), 2.54-2.42 (m, 2H), 2.31-2.16 (m, 3H), 2.07 (q, *J* = 9.0 Hz, 1H), 1.67 (s, 1H), 1.18 (d, *J* = 7.1 Hz, 3H); ^{13}C NMR (125 MHz, $CDCl_3$): δ = 218.4, 174.8, 152.5, 143.7, 115.3, 83.6, 75.7, 51.0, 49.3, 48.8, 47.6, 47.5, 47.3, 43.6, 39.6, 14.3; HR-MS (ESI): *m/z* calcd for $C_{17}H_{22}N_3O_4$ $[M+H]^+$: 332.1610; found: 332.1598.

(3R,3aR,4S,6aR,9S,9aR,9bR)-3-((1H-1,2,3-triazol-1-yl)methyl)-4-hydroxy-9-methyl-6-methyleneoctahydroazuleno[4,5-b]furan-2,8(3H,9bH)-dione **(11d)**: Prepared with **1** and 1,2,3-triazole. Yield: 66%; white crystals; m.p.: 86.5-87.2 °C; $[\alpha]_D^{20}$ = +90.1 (c 0.19, MeOH); ^{1}H NMR (500 MHz, $CDCl_3$): δ = 7.67 (s, 2H), 5.10-5.04 (m, 3H), 4.74 (s, 1H), 3.98 (t, *J* = 9.4 Hz, 1H), 3.75 (tt, *J* = 6.9, 9.4 Hz, 1H), 3.09 (dt, *J* = 3.8, 11.0 Hz, 1H), 2.96-2.90 (m, 2H), 2.86 (dd, *J* = 5.6, 12.5 Hz, 1H), 2.54-2.41 (m, 2H), 2.23 (quin, *J* = 7.3 Hz, 1H), 2.15-2.00 (m, 3H), 1.21 (d, *J* = 7.1 Hz, 3H); ^{13}C NMR (125 MHz, $CDCl_3$): δ = 174.7, 134.9, 115.3, 83.4, 75.6, 53.3, 51.2, 49.2, 49.1, 47.4, 46.9, 43.7, 39.6, 14.4; HR-MS (ESI): *m/z* calcd for $C_{17}H_{22}N_3O_4$ $[M+H]^+$: 332.1610; found: 332.1599.

(3R,3aR,4S,6aR,9S,9aR,9bR)-3-((1H-indol-1-yl)methyl)-4-hydroxy-9-methyl-6-methyleneoctahydroazuleno[4,5-b]furan-2,8(3H,9bH)-dione (**11e**): Prepared with **1** and indole. Yield: 35%; white crystals; m.p.: 163.4-164.6 °C; $[\alpha]_D^{20}$ = +141.20 (c 0.10, MeOH); ^{1}H NMR (500 MHz, $CDCl_3$): δ = 7.66 (d, *J* = 8.3 Hz, 1H), 7.62 (d, *J* = 7.8 Hz, 1H), 7.23 (dd, *J* = 3.1, 13.5 Hz, 2H), 7.11 (t, *J* = 7.5 Hz,1H), 6.52 (d, *J* = 3.0 Hz, 1H), 5.01 (s, 1H), 4.80-4.68 (m, 2H), 4.66 (s, 1H), 3.87 (t, *J* = 9.3 Hz, 1H), 3.85-3.77 (m, 1H), 3.04-2.98 (m, 1H), 2.77 (dd, *J* = 5.5, 12.3 Hz, 1H), 2.66 (q, *J* = 7.5 Hz, 1H), 2.41-2.30 (m, 2H), 2.22-2.06 (m, 3H), 1.95 (d, *J* = 3.9 Hz, 1H), 1.79 (q, *J* = 8.9 Hz, 1H), 1.13 (d, *J* = 7.1 Hz, 3H); ^{13}C NMR (125 MHz, $CDCl_3$): δ = 176.6, 143.7, 129.3, 128.5, 121.9, 121.0, 119.7, 115.2, 110.4, 102.6, 83.9, 75.9, 51.1, 49.7, 47.9, 47.3, 46.7, 45.0, 43.7, 39.3, 14.1; HR-MS (ESI): *m/z* calcd for $C_{23}H_{26}NO_4$ $[M+H]^+$: 380.1861; found: 380.1849.

(3R,3aR,4S,6aR,9S,9aR,9bR)-3-((1H-benzo[d]imidazol-1-yl)methyl)-4-hydroxy-9-methyl-6-methyleneoctahydroazuleno[4,5-b]furan-2,8(3H,9bH)-dione **(11f)**: Prepared with **1** and benzimidazole Yield: 50%; white crystals; m.p.: 262.6-263.7 °C; $[\alpha]_D^{20}$ = +115.73 (c 0.17, MeOH); ^{1}H NMR (500 MHz, CD_3OD): δ = 8.22 (s, 1H), 7.92 (d, *J* = 8.0 Hz, 1H), 7.70 (d, *J* = 8.0 Hz, 1H), 7.34 (dt, *J* = 7.3, 26.3 Hz, 2H), 5.09 (s, 1H), 4.91 (dd, *J* = 2.5, 14.7 Hz, 1H), 4.77 (dd, *J* = 6.6, 14.8 Hz, 1H), 4.72 (s, 1H), 4.11 (t, *J* = 9.4 Hz, 1H), 3.87 (q, *J* = 4.3 Hz, 1H), 3.28-3.21 (m, 1H), 3.02 (t, *J* = 7.5 Hz, 1H), 2.88 (dd, *J* = 5.5, 12.4 Hz, 1H), 2.47-2.35 (m, 2H), 2.30-2.18 (m, 2H), 2.09(q, *J* = 9.0 Hz, 1H), 1.14 (d, *J* = 7.0 Hz, 3H); ^{13}C NMR (125 MHz, CD_3OD): δ = 178.3, 146.3, 143.7, 124.3, 123.6, 120.0, 114.5, 112.4, 85.4, 76.2, 52.1, 50.2, 49.7, 49.6, 48.3, 47.9, 45.3, 44.5, 40.68, 40.60, 14.36, 14.34; HR-MS (ESI): *m/z* calcd for $C_{22}H_{25}N_2O_4$ $[M+H]^+$: 381.1814; found: 381.1802.

(3R,3aR,4S,6aR,9S,9aR,9bR)-3-((1H-indazol-1-yl)methyl)-4-hydroxy-9-methyl-6-methyleneoctahydroazuleno[4,5-b]furan-2,8(3H,9bH)-dione **(11g)**: Prepared with **1** and indazole. Yield: 64%; white crystals; m.p.: 172.4-173.5 °C; $[\alpha]_D^{20}$ = +160.40 (c 0.10, MeOH); ^{1}H NMR (500 MHz, $CDCl_3$): δ = 8,05 (s, 1H), 7.73 (d, *J* = 8.2 Hz, 1H), 7.67 (d, *J* = 8.6 Hz, 1H), 7.45 (t, *J* = 7.5 Hz, 1H), 7.19 (t, *J* = 7.5 Hz, 1H), 5.10-4.96 (m, 3H), 4.91 (d, *J* = 5.9 Hz, 1H), 4.72 (s, 1H), 3.97 (t, *J* = 9.4 Hz, 1H), 3.75 (s, 1H), 3.02 (dd, *J* = 4.9, 11.6 Hz, 1H), 2.94 (dd, *J* = 5.4, 12.6 Hz, 1H), 2.87 (q, *J* = 6.4 Hz,

1H), 2.41 (d, *J* = 6.1 Hz, 2H), 2.26-2.10 (m, 3H), 1.96 (q, *J* = 9.0 Hz, 1H), 1.13 (d, *J* = 7.1 Hz, 3H); ^{13}C NMR (125 MHz, $CDCl_3$): δ = 175.9, 143.8, 140.5, 134.0, 127.7, 123.6, 121.6, 121.1, 114.8, 110.3, 82.1, 78.3, 75.6, 54.5, 50.7, 48.7, 47.4, 47.3, 46.5, 42.4, 38.3, 18.2; HR-MS (ESI): *m/z* calcd for $C_{22}H_{25}N_2O_4$ $[M+H]^+$: 381.1814; found: 381.1801.

(3R,3aR,4S,6aR,9S,9aR,9bR)-3-((1H-benzo[d][1,2,3]triazol-1-yl)methyl)-4-hydroxy-9-methyl-6-methyleneoctahydroazuleno[4,5-b]furan-2,8(3H,9bH)-dione **(11h)**: Prepared with **1** and benzotriazole. Yield: 34%; white crystals; m.p.: 166.4-168.2 °C; $[\alpha]_D^{20}$ = +88.0 (c 0.14, MeOH); ^{1}H NMR (500 MHz, $CDCl_3$): δ = 8.05 (d, *J* = 8.3 Hz, 1H), 7.78 (d, *J* = 8.4 Hz, 1H), 7.54 (t, *J* = 7.4 Hz, 1H), 7.40 (t, *J* = 7.6 Hz, 1H), 5.33 (dd, *J* = 5.1, 14.7 Hz, 1H), 5.23 (dd, *J* = 2.0, 14.6 Hz, 1H), 5.07 (s, 1H), 4.72(s, 1H), 3.99 (t, *J* = 9.3 Hz, 1H), 3.84-3.75 (m, 1H), 3.23-3.17 (m, 1H), 2.97-2.88 (m, 3H), 2.45-2.33 (m, 3H), 2.25-2.14 (m, 2H), 1.94 (q, *J* = 8.9 Hz, 1H), 1.11 (d, *J* = 7.1 Hz, 3H); ^{13}C NMR (125 MHz, $CDCl_3$): δ =218.5, 175.2, 145.8, 143.8, 133.8, 128.5, 124.7, 119.9, 115.2, 110.7, 84.1, 76.1, 50.9, 49.7, 48.6, 47.3, 46.2, 43.7, 39.5, 14.2; HR-MS (ESI): *m/z* calcd for $C_{21}H_{24}N_3O_4$ $[M+H]^+$: 382.1766; found: 382.1754.

(3R,3aR,4S,6aR,8S,9S,9aR,9bR)-3-((1H-benzo[d]imidazol-1-yl)methyl)-4,8-dihydroxy-9-methyl-6-methylenedecahydroazuleno[4,5-b]furan-2(9bH)-one **(16a)**: Prepared with **12** and benzimidazole. Yield: 62%; white crystals; m.p.: 187.3-188.2 °C; $[\alpha]_D^{20}$ = +35.1 (c 0.16, MeOH); ^{1}H NMR (500 MHz, CD_3OD): δ = 8.22 (s, 1H), 7.86 (d, *J* = 8.0 Hz, 1H), 7.68 (d, *J* = 7.9 Hz, 1H), 7.31 (dt, *J* = 7.2, 21.0 Hz, 2H), 5.04 (d, *J* = 4.8 Hz, 2H), 4.89 (dd, *J* = 2.2, 14.6 Hz, 1H), 4.71 (dd, *J* = 6.6, 14.7 Hz, 1H), 4.52 (s, 2H), 4.02 (t, *J* = 9.7 Hz, 1H), 3.77 (td, *J* = 4.3, 10.0 Hz, 1H), 3.55 (q, *J* = 9.1 Hz, 1H), 3.18-3.11 (m, 1H), 2.80 (dd, *J* = 4.3, 12.3 Hz, 1H), 2.74 (q, *J* = 9.2 Hz, 1H), 2.15-1.99 (m, 3H), 1.77-1.60 (m, 3H), 1.08 (d, *J* = 5.8 Hz, 3H); ^{13}C NMR (125 MHz, CD_3OD): δ = 178.6, 145.7, 135.4, 124.3, 123.6, 120.0, 114.9, 112.4, 83.5, 78.6, 76.6, 54.0, 52.0, 47.7, 45.2, 43.1, 39.2, 18.1; HR-MS (ESI): *m/z* calcd for $C_{22}H_{27}N_2O_4$ $[M+H]^+$: 383.1970; found: 383.1958.

(3R,3aR,4S,6aR,8S,9S,9aR,9bR)-3-((1H-indazol-1-yl)methyl)-4,8-dihydroxy-9-methyl-6-methylenedecahydroazuleno[4,5-b]furan-2(9bH)-one **(16b)**: Prepared with **12** and indazole. Yield: 32%; white crystals; m.p.: 168.7-169.9 °C; $[\alpha]_D^{20}$ = +66.2 (c 0.10, MeOH); ^{1}H NMR (500 MHz, $CDCl_3$): δ = 8,03 (s, 1H), 7.71 (dd, *J* = 8.2, 13.2 Hz, 2H), 7.44 (t, *J* = 7.5 Hz, 1H), 7.18 (t, *J* = 7.4 Hz, 1H), 5.50 (d, *J* = 7.6 Hz, 1H), 5.03 (s, 1H), 4.98 (d, *J* = 4.2 Hz, 3H), 3.96 (t, *J* = 10.1 Hz, 1H), 3.70 (d, *J* = 3.2 Hz, 1H), 3.63 (q, *J* = 8.6 Hz, 1H), 2.88 (dd, *J* = 3.7, 12.4 Hz, 2H), 2.71 (q, *J* = 9.9 Hz, 1H), 2.12-1.97 (m, 3H), 1.79 (q, *J* = 7.6 Hz, 1H), 1.73-1.60 (m, 3H), 1.07 (d, *J* = 6.5 Hz, 3H); ^{13}C NMR (125 MHz, $CDCl_3$): δ = 175.9, 143.8, 140.5, 134.0, 127.7, 123.6, 121.6, 121.1, 114.8, 110.3, 82.1, 78.3, 75.6, 54.5, 50.7, 48.7, 47.4, 47.3, 46.5, 42.4, 38.3, 18.2; HR-MS (ESI): *m/z* calcd for $C_{22}H_{27}N_2O_4$ $[M+H]^+$: 383.1970; found: 383.1961.

(3R,3aR,4S,6aR,8S,9S,9aR,9bR)-3-((1H-benzo[d][1,2,3]triazol-1-yl)methyl)-4,8-dihydroxy-9-methyl-6-methylenedecahydroazuleno[4,5-b]furan-2(9bH)-one **(16c)**: Prepared with **12** and benzotriazole. Yield: 37%; white crystals; m.p.: 172.5-173.4 °C; $[\alpha]_D^{20}$ = +30.0 (c 0.10, MeOH); ^{1}H NMR (500 MHz, $CDCl_3$): δ = 8,04 (d, *J* = 8.4 Hz, 1H), 7.79 (d, *J* = 8.4 Hz, 1H), 7.53 (t, *J* = 7.3 Hz, 1H), 7.40 (t, *J* = 7.5 Hz, 1H), 5.29 (dd, *J* = 4.9, 14.6 Hz, 1H), 5.19 (d, *J* = 14.6 Hz, 1H), 5.04 (d, *J* = 10.6 Hz, 2H), 4.56 (s, 4H), 3.99 (t, *J* = 10.1 Hz, 1H), 3.73 (q, *J* = 6.2 Hz, 1H), 3.62 (q, *J* = 8.7 Hz, 1H), 3.31 (s, 1H), 3.12 (dt, *J* = 2.2, 12.0 Hz, 3H), 2.88 (dd, *J* = 12.4, 4.2 Hz, 1H), 2.70 (q, *J* = 9.6 Hz, 1H), 2.14-2.03 (m, 3H), 1.80-1.57 (m, 3H), 1.02 (d, *J* = 6.5 Hz, 3H); ^{13}C NMR (125 MHz, $CDCl_3$): δ =175.3, 145.7, 143.4, 133.9, 128.4, 124.7, 119.8, 115.4, 110.9, 82.2, 78.2, 76.2, 53.1, 50.8, 48.1, 47.7, 47.1, 46.1, 42.2, 38.3, 17.9; HR-MS (ESI): *m/z* calcd for $C_{21}H_{26}N_3O_4$ $[M+H]^+$: 384.1923; found: 384.1913.

2.6. General procedure for grossheimin-based amino- and thioadducts

Compound **1** or **12** (0.11 mmol) was dissolved in dry EtOH (2.0 mL), benzylamine or benzylmercaptane (0.22 mmol) was added. The mixture was stirred at room temperature either in presence of Et_3N (0.11 mmol) or without the base. After the reaction was complete, the mixture was evaporated under vacuum. The residue was purified by column chromatography with $CHCl_3$:MeOH = 19:1 to give the desired products.

(3R,3aR,4S,6aR,9S,9aR,9bR)-3-((benzylamino)methyl)-4-hydroxy-9-methyl-6-methyleneoctahydroazuleno[4,5-b]furan-2,8(3H,9bH)-dione **(5)**: Prepared with **1** and benzylamine**.** Yield: 68%; white crystals; m.p.: 110.9-111.8 °C; $[\alpha]_D^{20}$ = +73.6 (c 0.16, MeOH); ^{1}H NMR (500 MHz, $CDCl_3$): δ = 7.39-7.27 (m, 5H), 5.07 (s, 1H), 4.77 (s, 1H), 4.02 (t, *J* = 9.6 Hz, 1H), 3.84 (q, *J* = 12.8 Hz, 2H), 3.68-3.60 (m, 1H), 3.55-3.47 (m, 1H), 3.12 (q, *J* = 8.0 Hz, 1H), 2.90 (dd, *J* = 5.7, 12.9 Hz, 1H), 2.68 (t, *J* = 10.9 Hz, 1H), 2.58-2.46 (m, 3H), 2.37-2.17 (m, 4H), 1.22 (d, *J* = 7.1 Hz, 3H); ^{13}C NMR (125 MHz, $CDCl_3$): δ =218.8, 174.9, 144.3, 135.9, 129.0, 128.6, 128.0, 114.8, 82.7, 73.8, 56.3, 53.8, 51.2, 49.7, 47.9, 47.4, 47.2, 43.3, 39.9, 14.9; HR-MS (ESI): *m/z* calcd for $C_{22}H_{28}NO_4$ $[M+H]^+$: 370.2018; found: 370.2006.

(3S,3aR,4S,6aR,9S,9aR,9bR)-3-((benzylthio)methyl)-4-hydroxy-9-methyl-6-methyleneoctahydroazuleno[4,5-b]furan-2,8(3H,9bH)-dione **(8)**: Prepared with **1** and benzylmercaptan. Yield: 66%; white crystals; m.p.: 141.9-142.4 °C; $[\alpha]_D^{20}$ = +56.6 (c 0.16, MeOH); ^{1}H NMR (500 MHz, $CDCl_3$): δ = 7.39-7.32 (m, 4H), 7.29-7.24 (m, 2H), 5.04 (s, 1H), 4.74 (s, 1H), 3.90 (t, *J* = 8.9 Hz, 1H), 3.79 (s, 2H), 3.63 (tt, *J* = 5.7, 9.9 Hz, 1H), 3.10-3.01 (m, 3H), 2.82 (q, *J* = 4.3 Hz, 1H), 2.77 (dd, *J* = 5.6, 12.4 Hz, 1H), 2.62-2.44 (m, 3H), 2.22 (d, *J* = 5.8 Hz, 2H), 2.09 (t, *J* = 10.4 Hz, 1H), 1.94 (d, *J* = 6.0 Hz, 1H), 1.23 (d, *J* = 6.4 Hz, 3H); ^{13}C NMR (125 MHz, $CDCl_3$): δ = 219.0, 176.2, 143.9, 138.6, 129.3, 128.8, 127.4, 115.1, 83.5, 75.4, 51.2, 49.8, 48.8, 47.4, 47.0, 43.7, 39.7, 37.7, 31.7, 14.5; HR-MS (ESI): *m/z* calcd for $C_{22}H_{27}O_4S$ $[M+H]^+$: 387.1630; found: 387.1610.

3. Cytotoxic effects of grossheimin-based aminoanalogues

Table S1. Cytotoxic effects of grossheimin-based aminoanalogues on colon adenocarcinoma cell lines and CDD-19Lu fibroblasts. Doxorubicin (DOX) was applied as a positive control . n.d: not determined. Starting concentration of grossheimin-based aminoanalogs and DOX in the assay with CDD-19Lu fibroblasts is 100 and 8.62 μM, respectively.

Compound	IC_{50} (μM)		
	**Colo205**	**Colo320**	**CCD-19Lu**
**1**	23.33 ±2.05	27.89±2.00	>100
**2**	8.21±0.51	9.44±0.41	>100
**3**	>100	>100	>100
**4**	>100	>100	>100
**5**	>100	>100	>100
**6**	>100	>100	>100
**7**	>100	>100	n.d
**8**	>100	>100	>100
**9**	>100	>100	>100
**10**	>100	>100	n.d
**11a–e**	>100	>100	n.d
**11f**	>100	>100	>100
**11g–h**	>100	>100	n.d
**12**	63.79±1.32	53.65±1.60	>100
**13**	8.21±0.51	6.98±0.29	29.07±0.46
**14**	>100	>100	n.d
**15**	>100	>100	n.d
**16a**	>100	>100	>100
**16b–c**	>100	>100	n.d
**DOX**	3.21±0.03	4.36±0.08	>8.62

4. ^{1}H-, ^{13}C- JMOD NMR spectra of new compounds

^{1}H-NMR of *(3aR,4S,6aR,9S,9aR,9bR)-9-Methyl-3,6-dimethylene-2,8-dioxododecahydroazuleno[4,5-b]furan-4-yl acetate* **2**

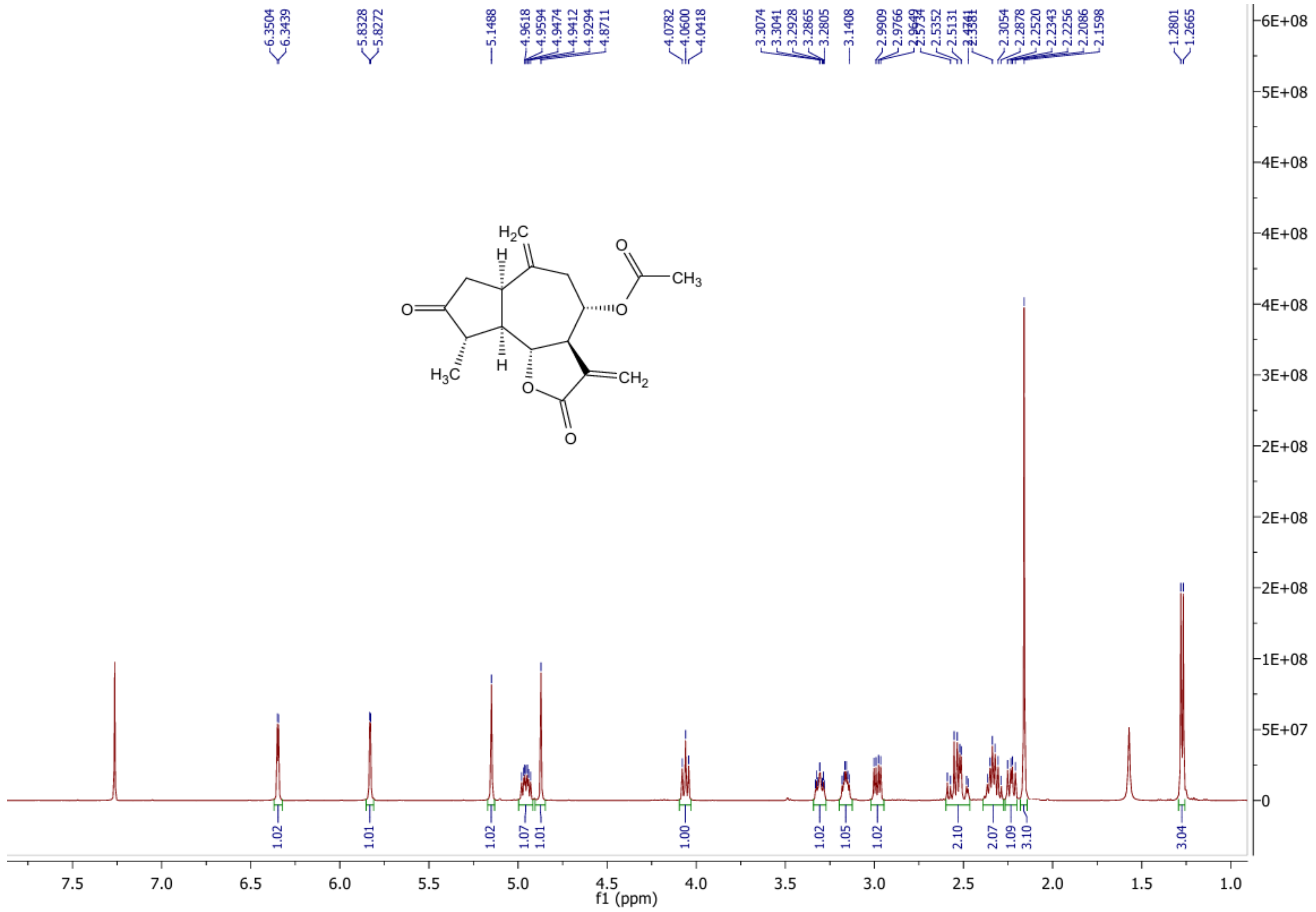

^{13}C-NMR of *(3aR,4S,6aR,9S,9aR,9bR)-9-Methyl-3,6-dimethylene-2,8-dioxododecahydroazuleno[4,5-b]furan-4-yl acetate* **2**

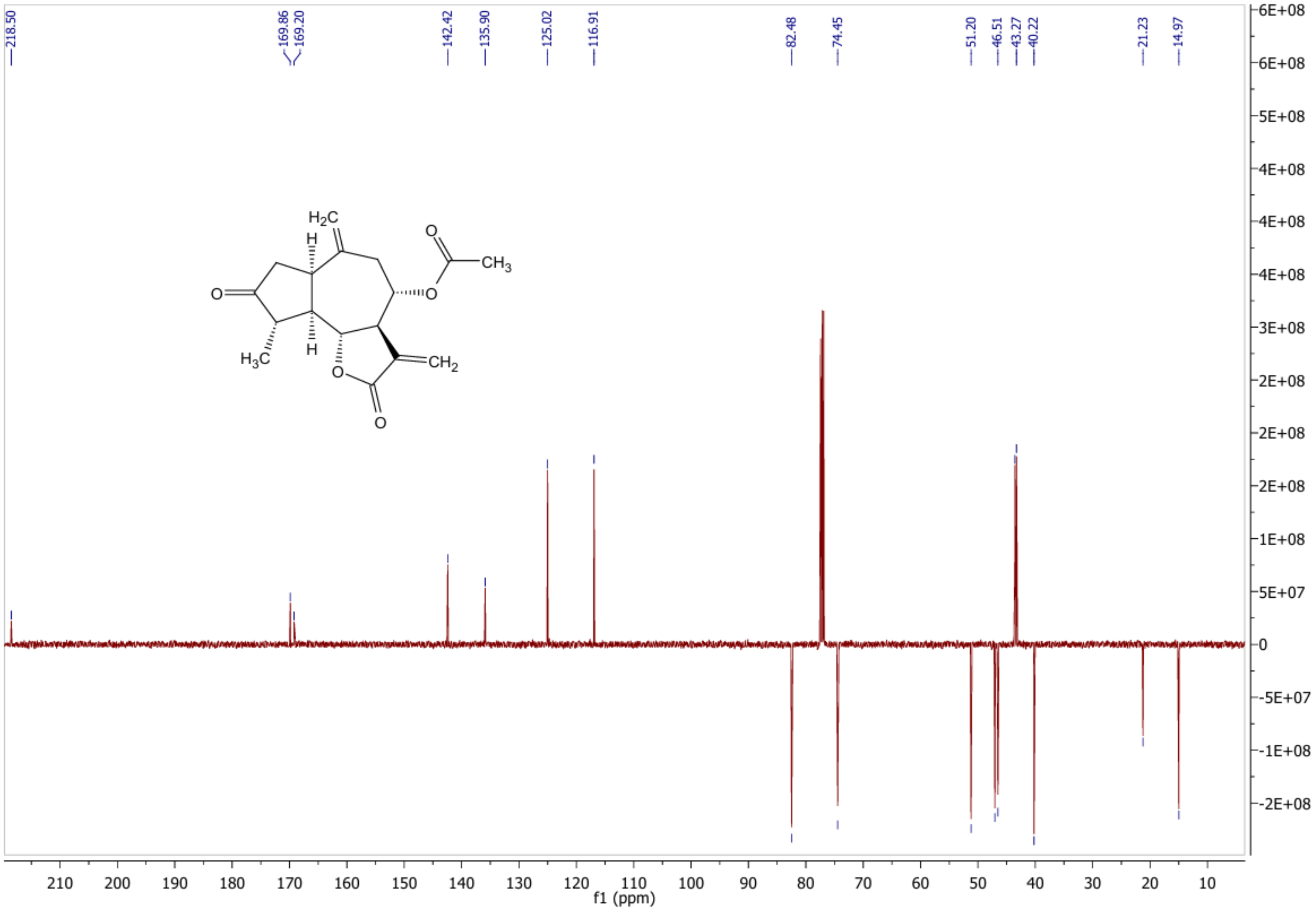

^{1}H-NMR of *(3S,3aR,4S,6aR,8R,9S,9aR,9bR)-4,8-Dihydroxy-3,9-dimethyl-6-methylenedecahydroazuleno[4,5-b]furan-2(9bH)-one* **3**

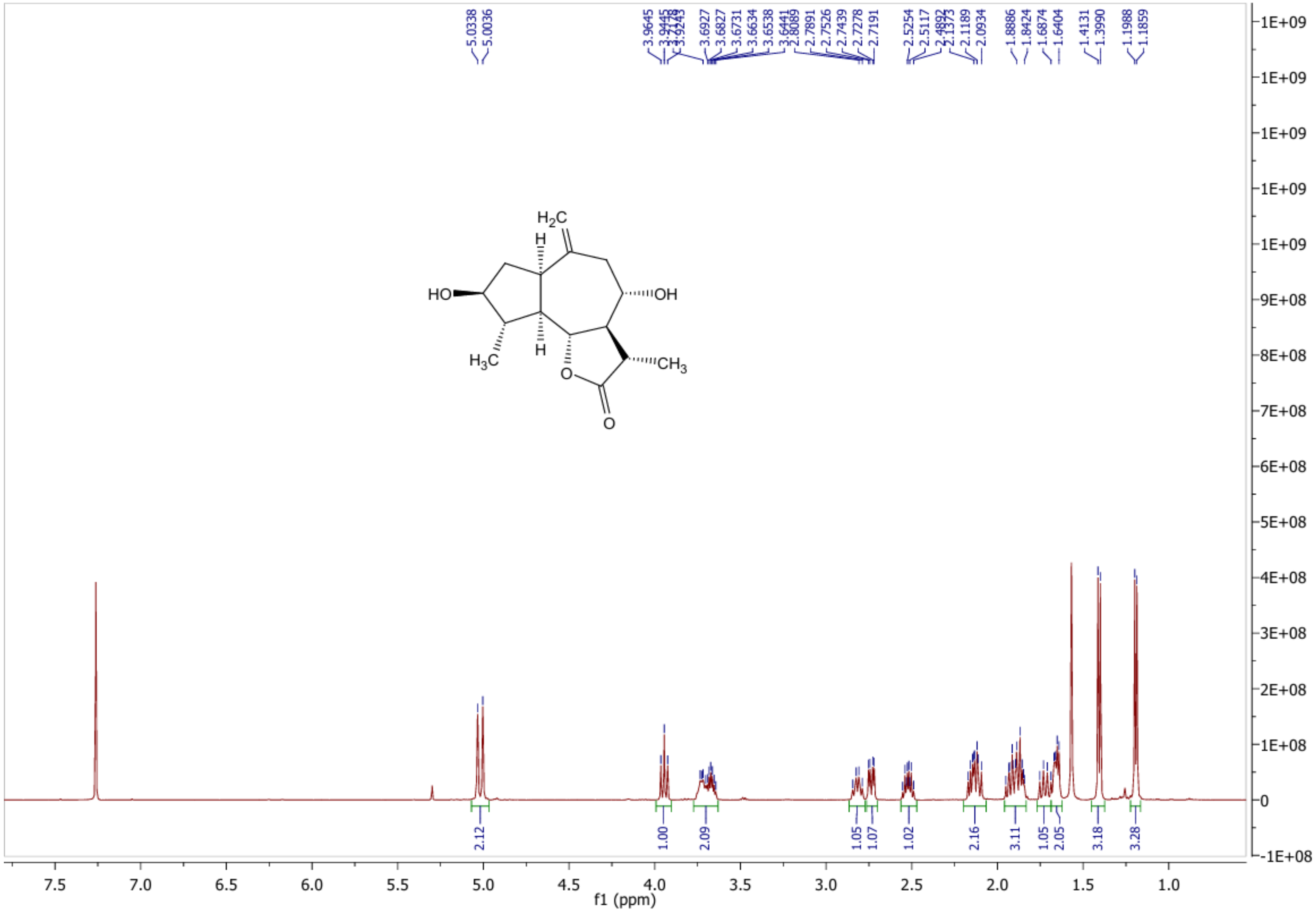

^{13}C-NMR of *(3S,3aR,4S,6aR,8R,9S,9aR,9bR)-4,8-Dihydroxy-3,9-dimethyl-6-methylenedecahydroazuleno[4,5-b]furan-2(9bH)-one* **3**

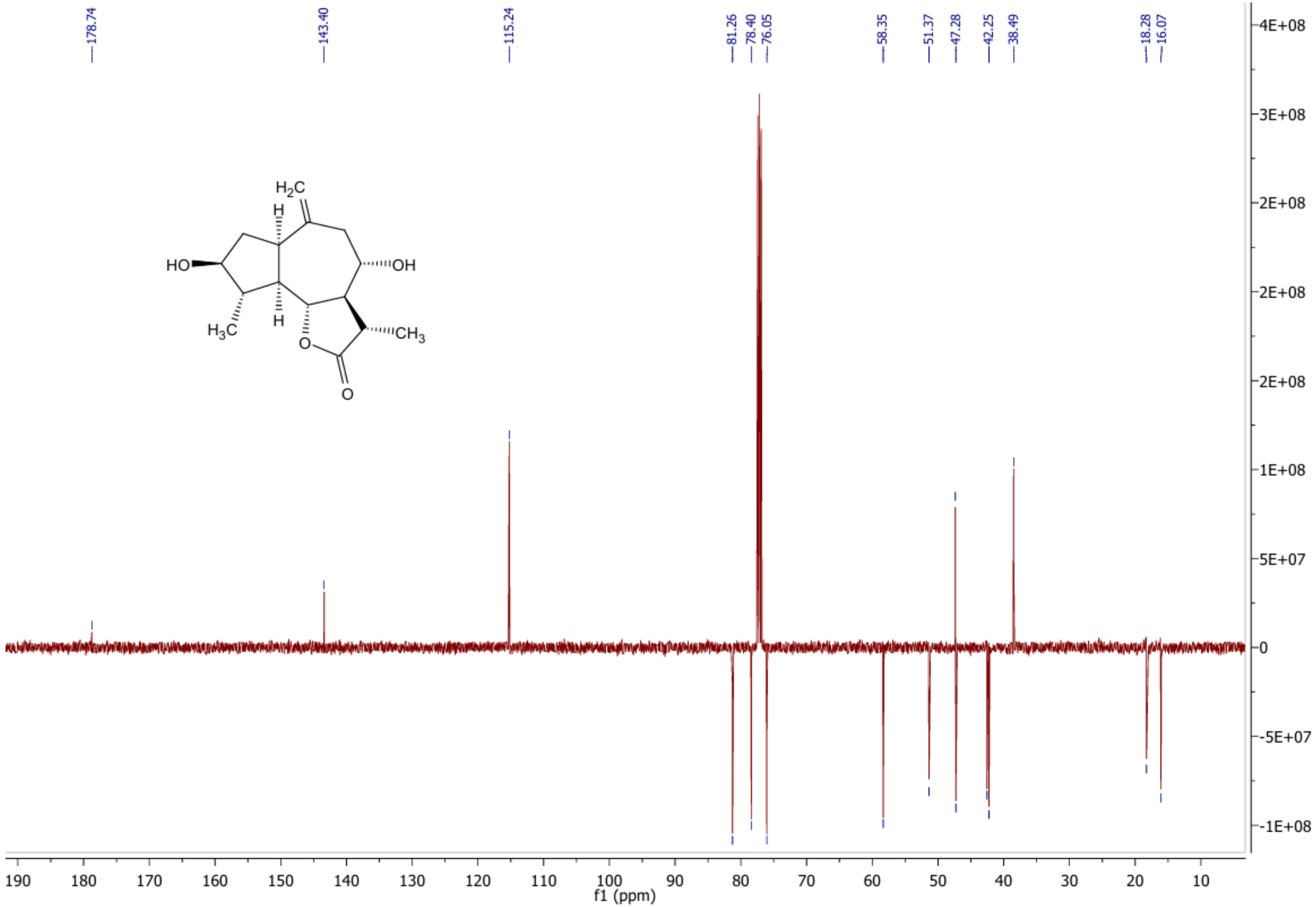

^{1}H-NMR of *(3S,3aR,4S,6aR,8S,9S,9aR,9bR)-3,9-dimethyl-6-methylene-2-oxododecahydroazuleno[4,5-b]furan-4,8-diyl diacetate* **4**

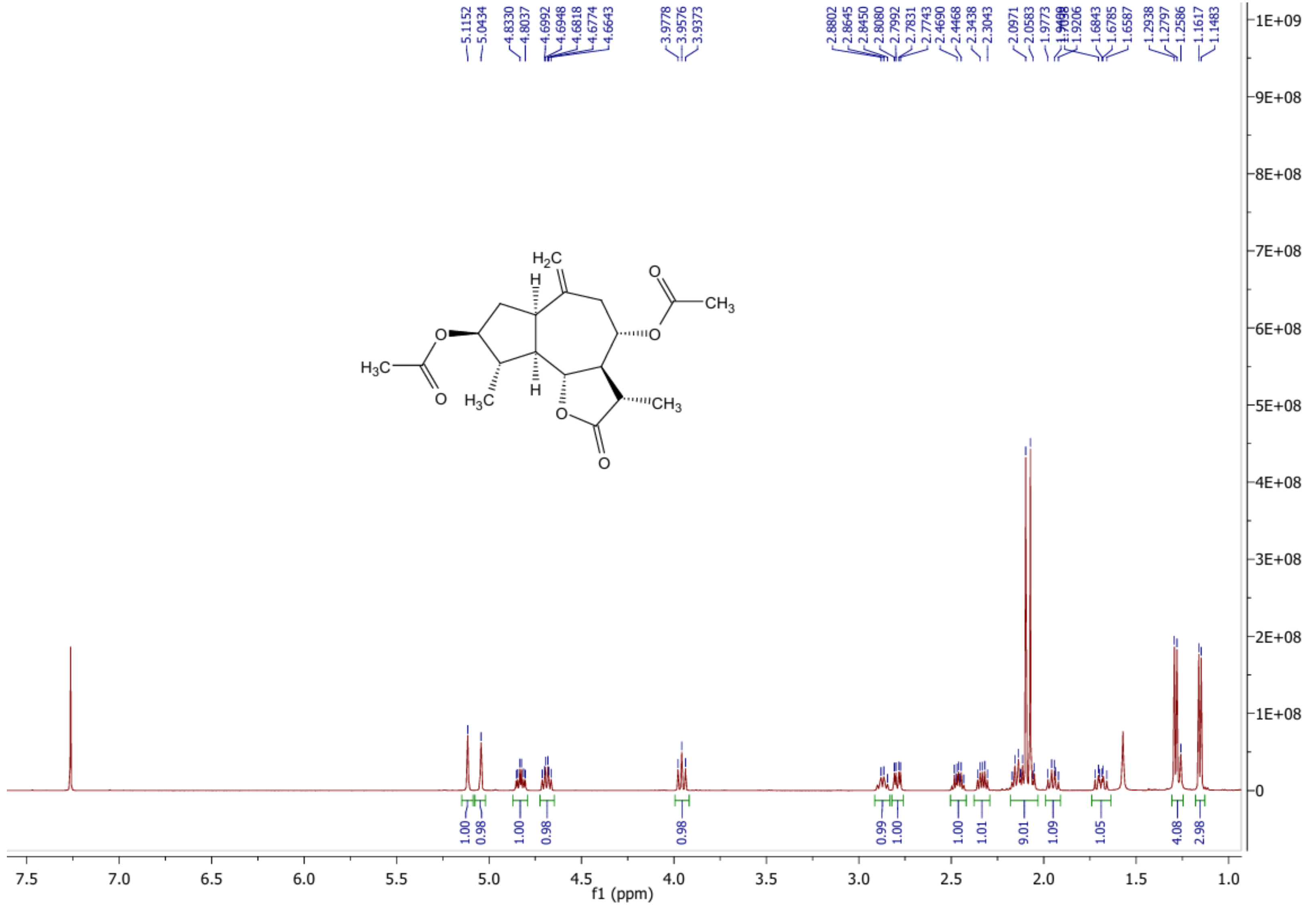

^{13}C-NMR of *(3S,3aR,4S,6aR,8S,9S,9aR,9bR)-3,9-dimethyl-6-methylene-2-oxododecahydroazuleno[4,5-b]furan-4,8-diyl diacetate* **4**

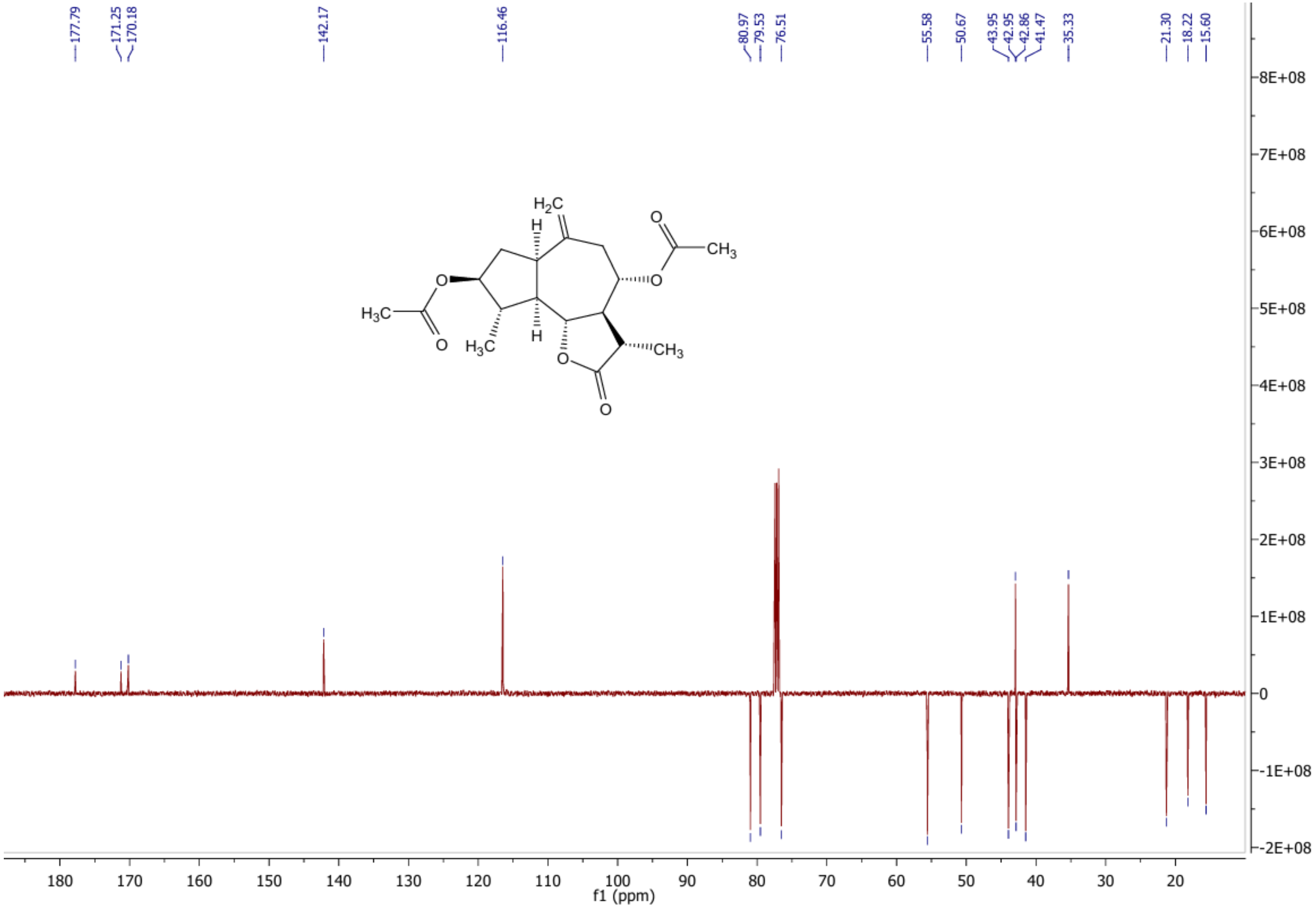

^{1}H-NMR of *(3R,3aR,4S,6aR,9S,9aR,9bR)-3-((benzylamino)methyl)-4-hydroxy-9-methyl-6-methyleneoctahydroazuleno[4,5-b]furan-2,8(3H,9bH)-dione* **5**

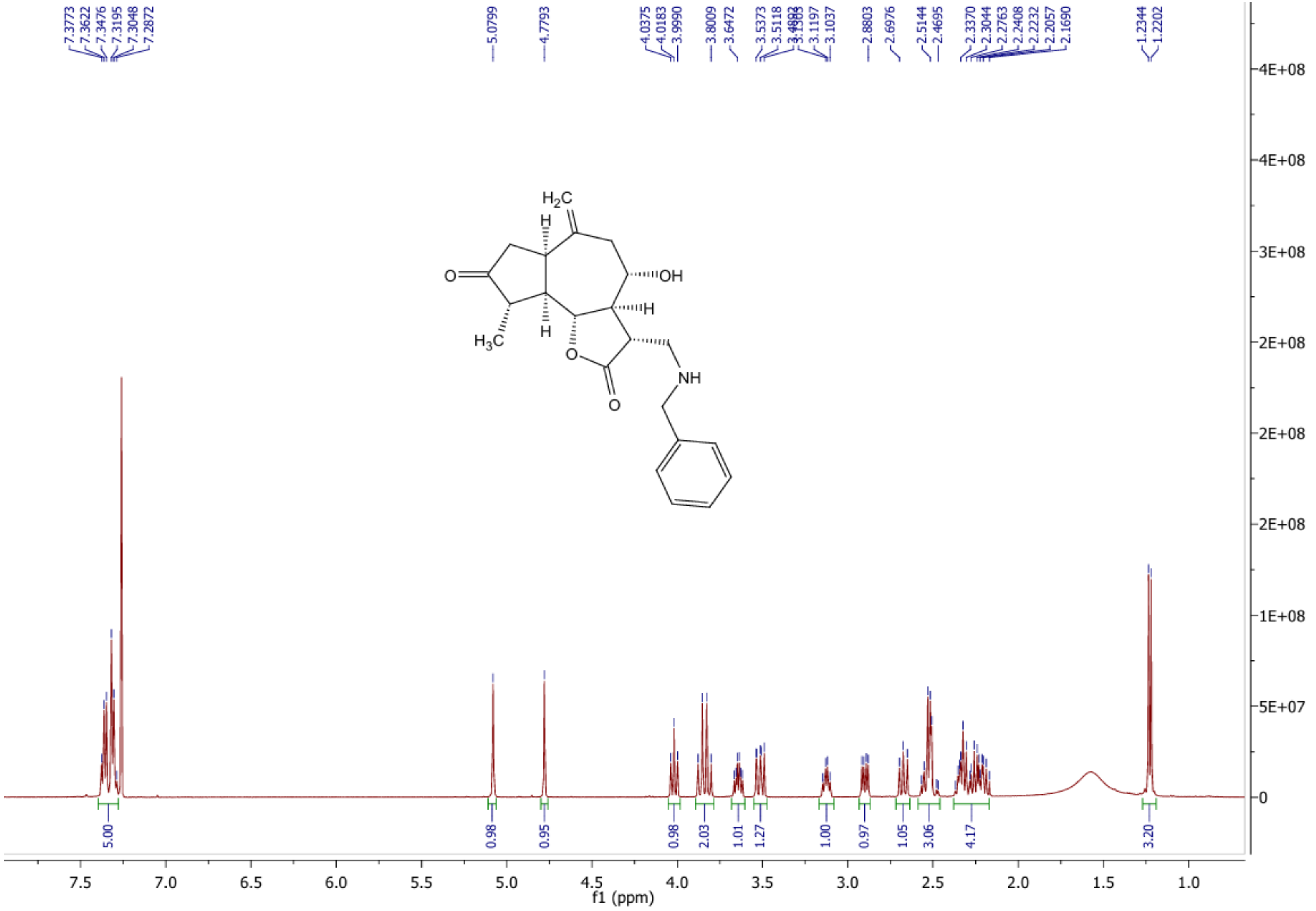

^{13}C-NMR of *(3R,3aR,4S,6aR,9S,9aR,9bR)-3-((benzylamino)methyl)-4-hydroxy-9-methyl-6-methyleneoctahydroazuleno[4,5-b]furan-2,8(3H,9bH)-dione* **5**

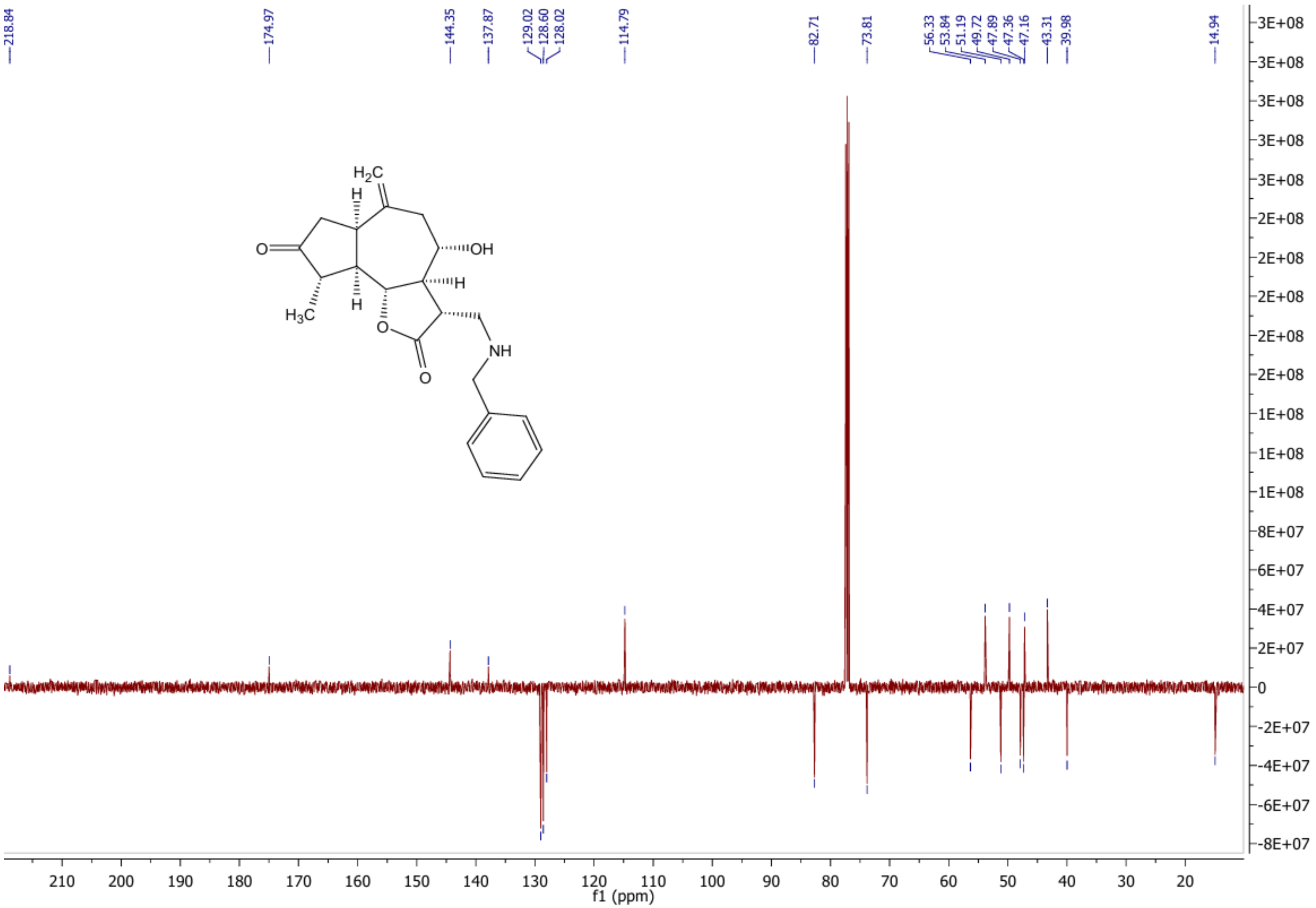

^{1}H-NMR of *3R,3aR,4S,6aR,8R,9S,9aR,9bR)-3-((Benzylamino)methyl)-4,8-dihydroxy-9-methyl-6-methylenedecahydroazuleno[4,5-b]furan-2(9bH)-one* **6**

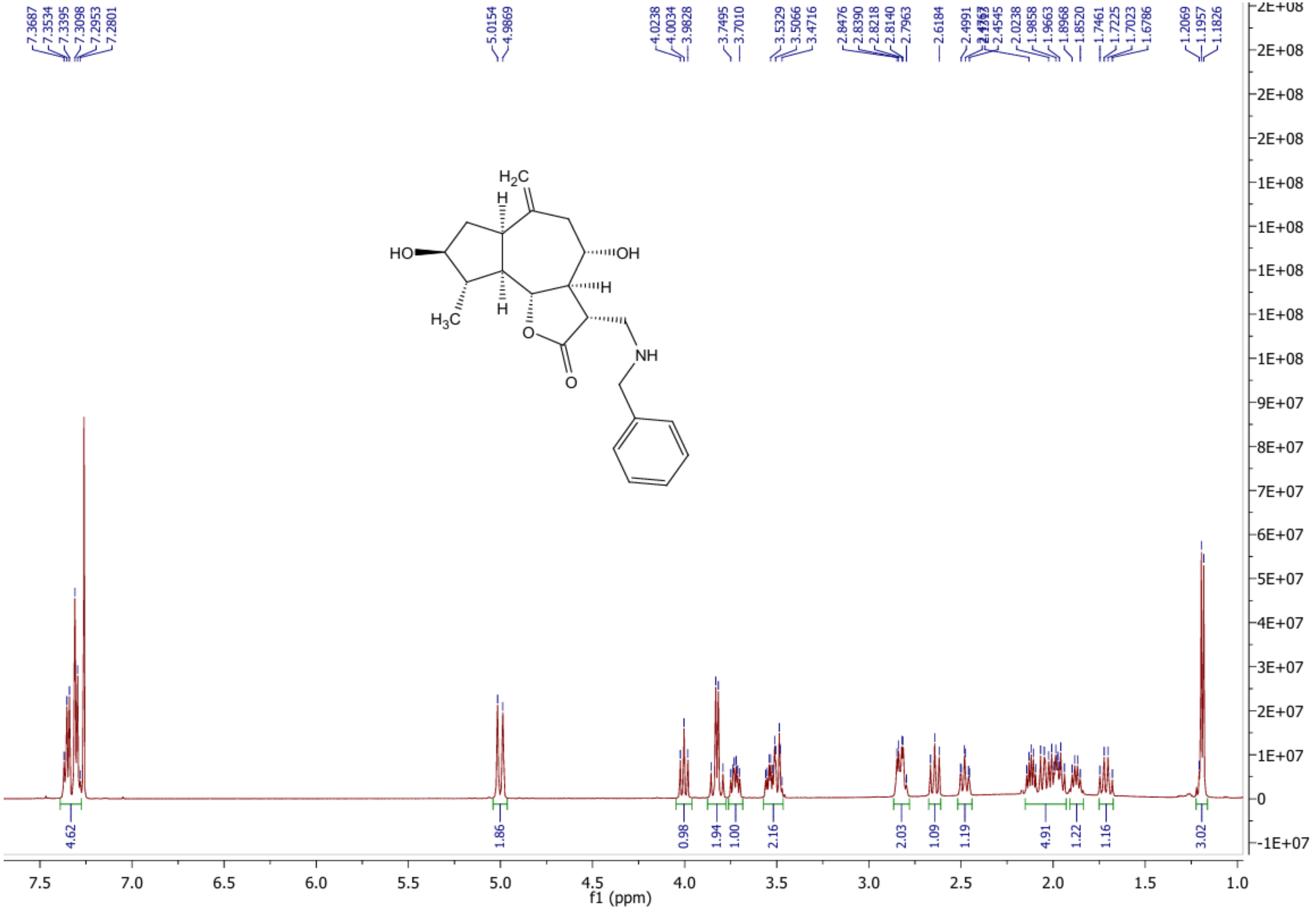

^{13}C-NMR of *3R,3aR,4S,6aR,8R,9S,9aR,9bR)-3-((Benzylamino)methyl)-4,8-dihydroxy-9-methyl-6-methylenedecahydroazuleno[4,5-b]furan-2(9bH)-one* **6**

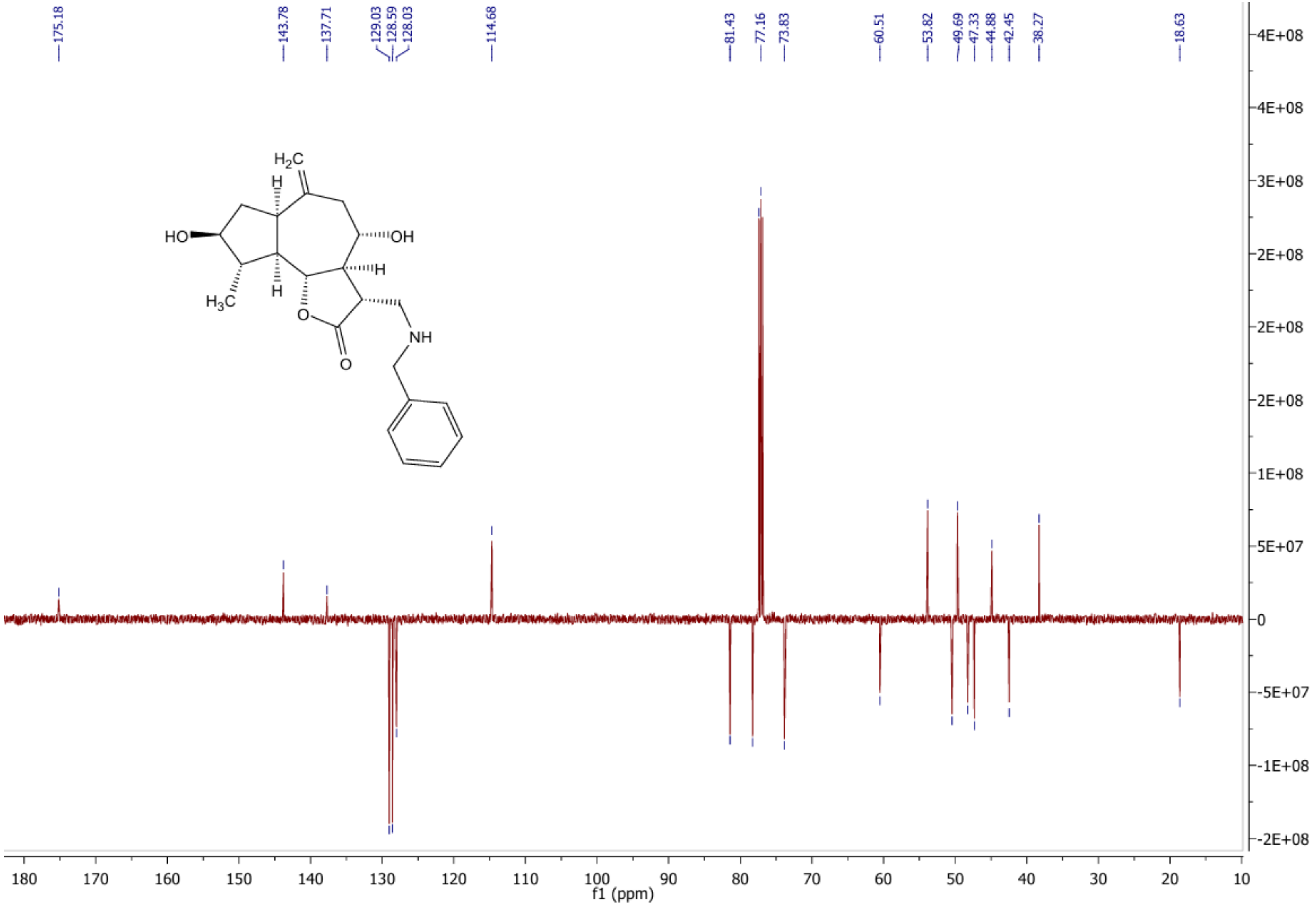

COSY spectrum of **6**

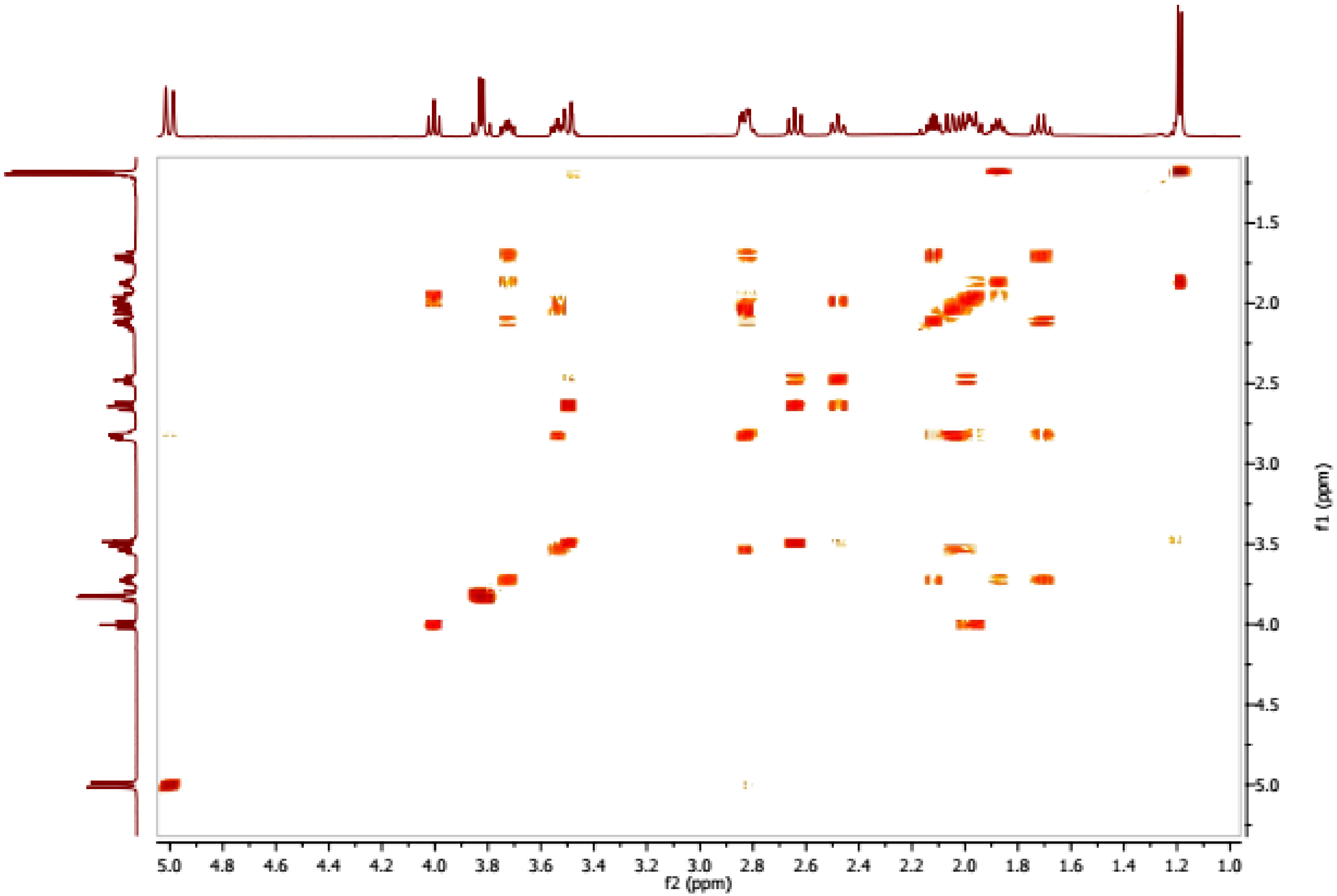

NOESY spectrum of **6**

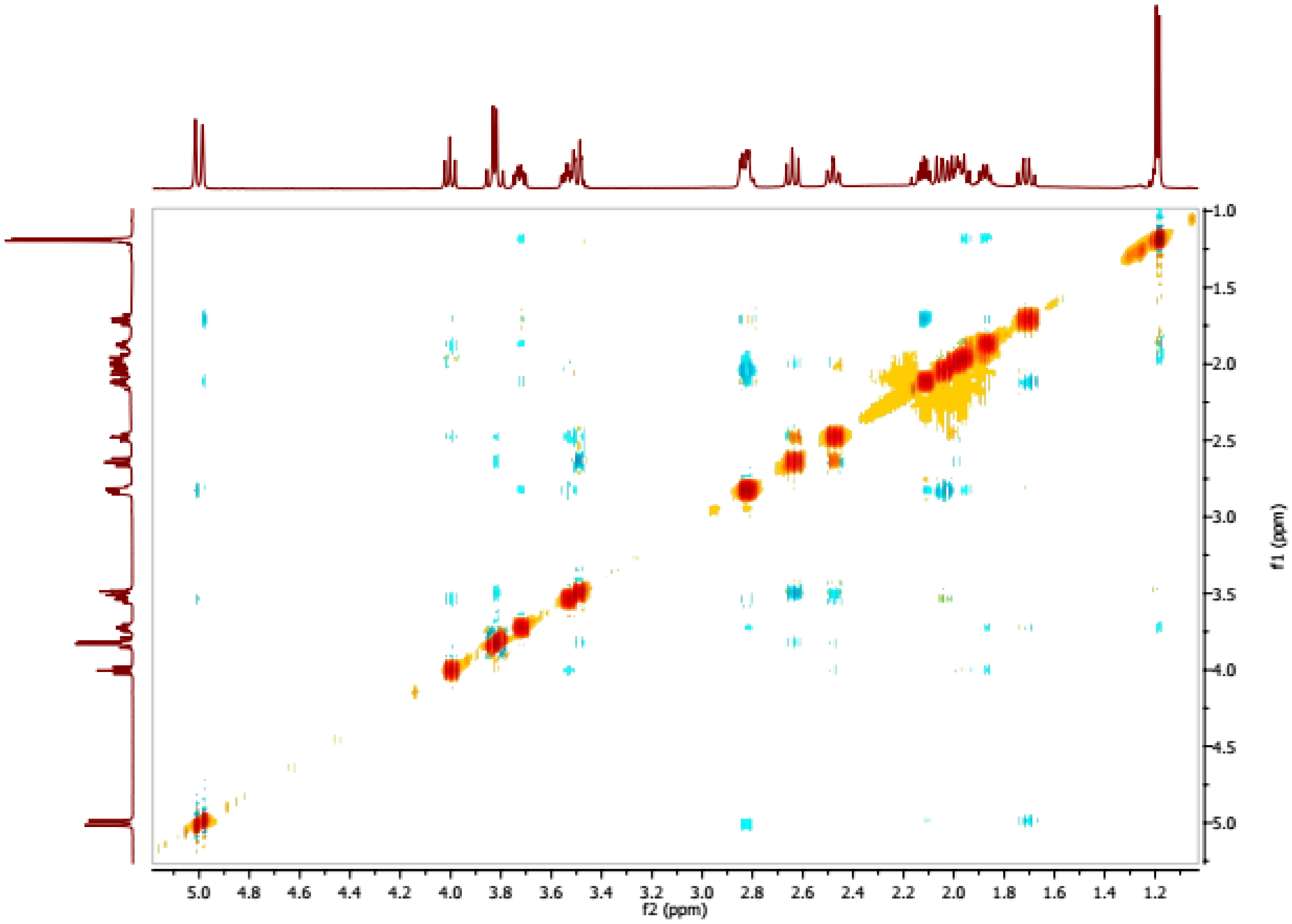

HSQC spectrum of **6**

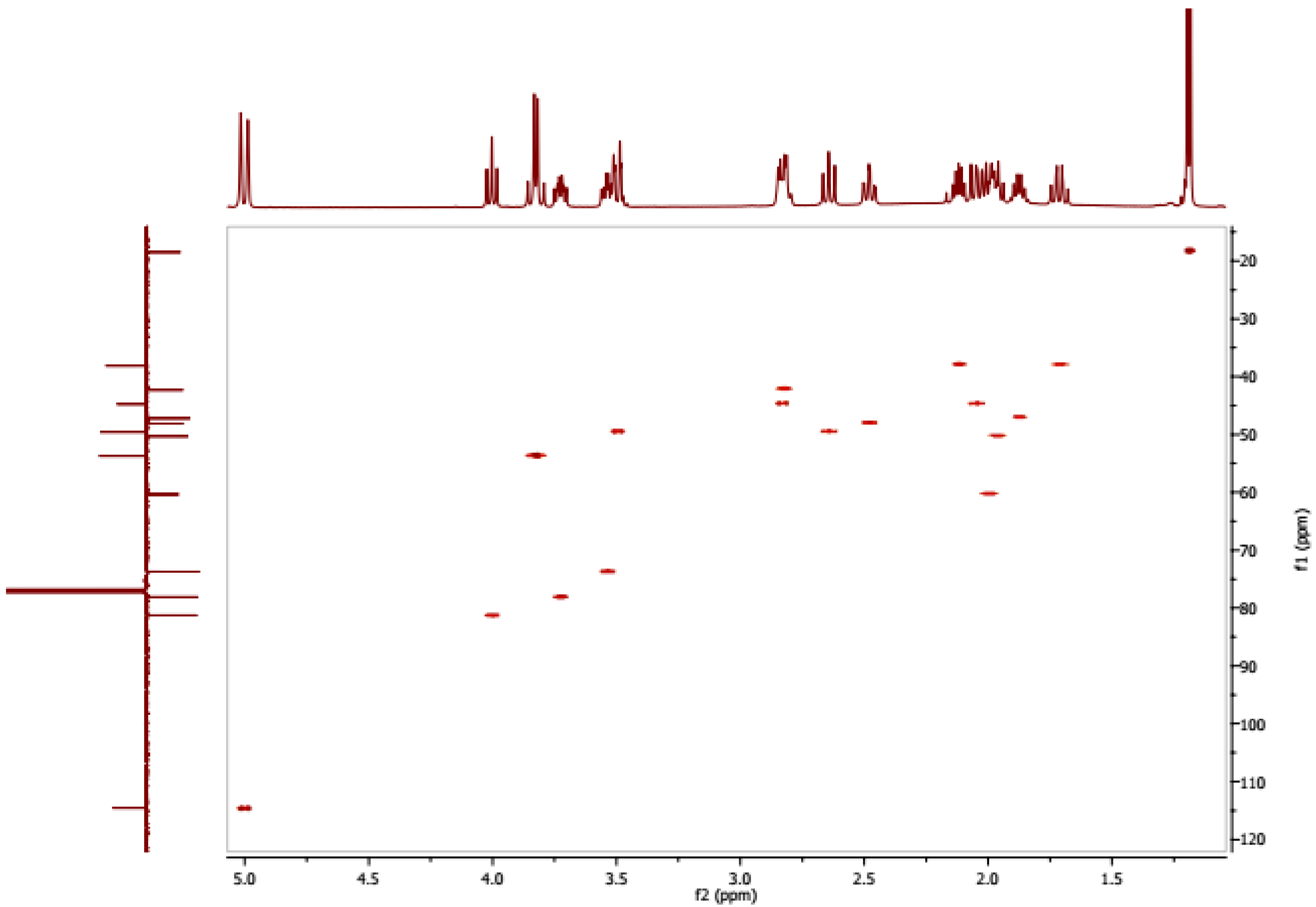

HMBC of compound **6**

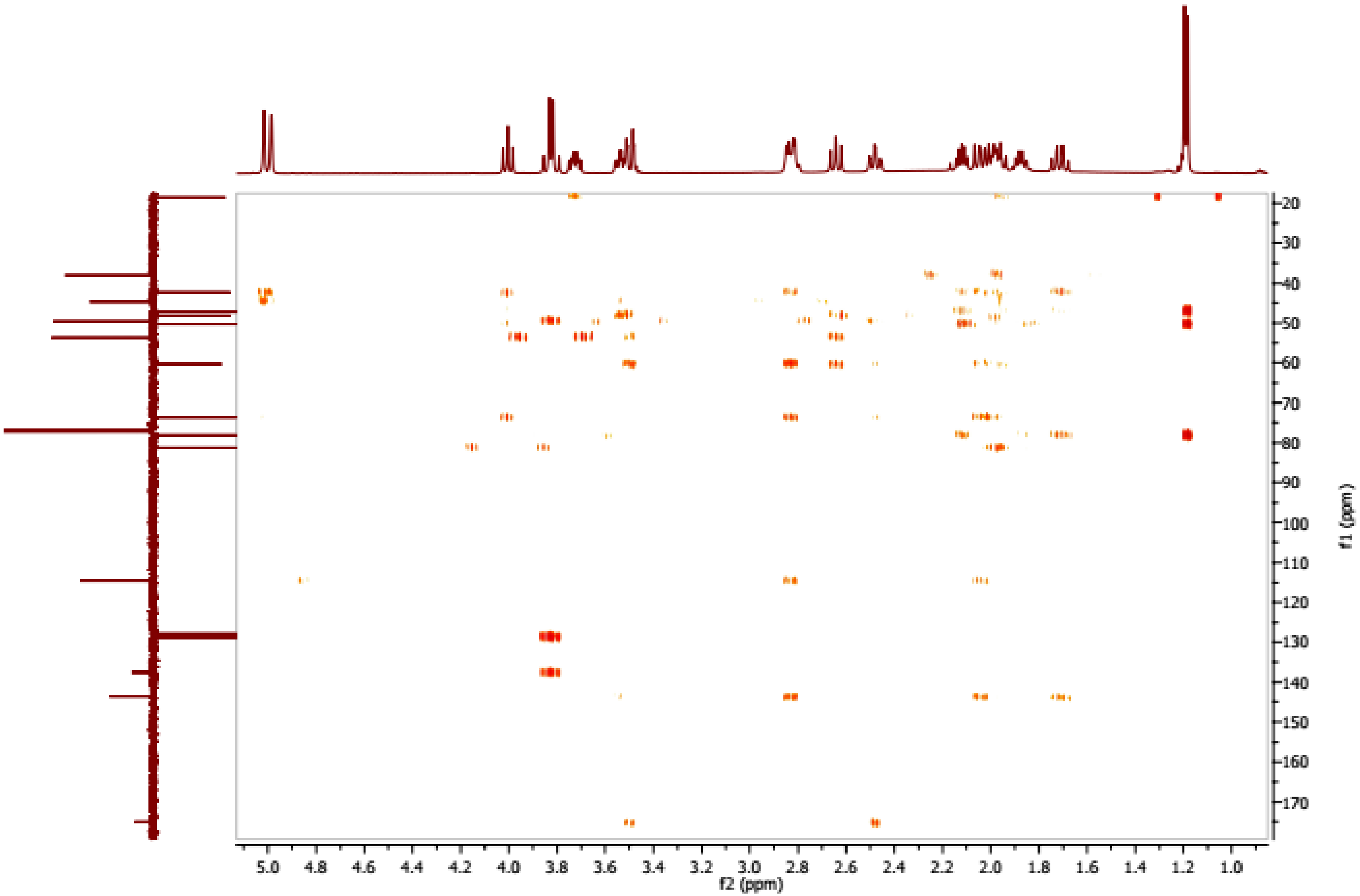

^{1}H-NMR of *(N-(((3R,3aR,4S,6aR,9S,9aR,9bR)-4-hydroxy-9-methyl-6-methylene-2,8-dioxododecahydroazuleno[4,5-b]furan-3-yl)methyl)formamide* **7**

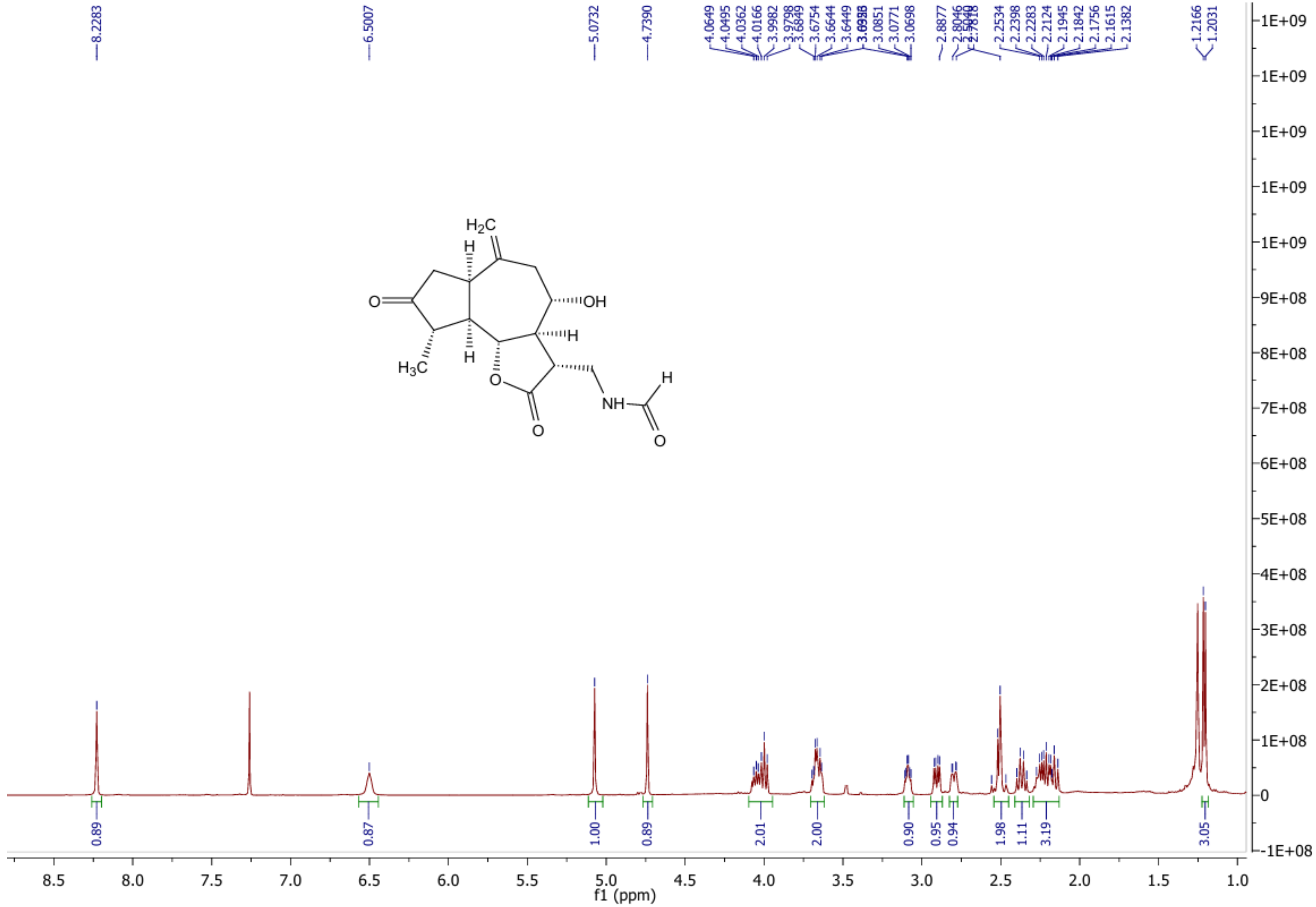

^{13}C-NMR of *(N-(((3R,3aR,4S,6aR,9S,9aR,9bR)-4-hydroxy-9-methyl-6-methylene-2,8-dioxododecahydroazuleno[4,5-b]furan-3-yl)methyl)formamide* **7**

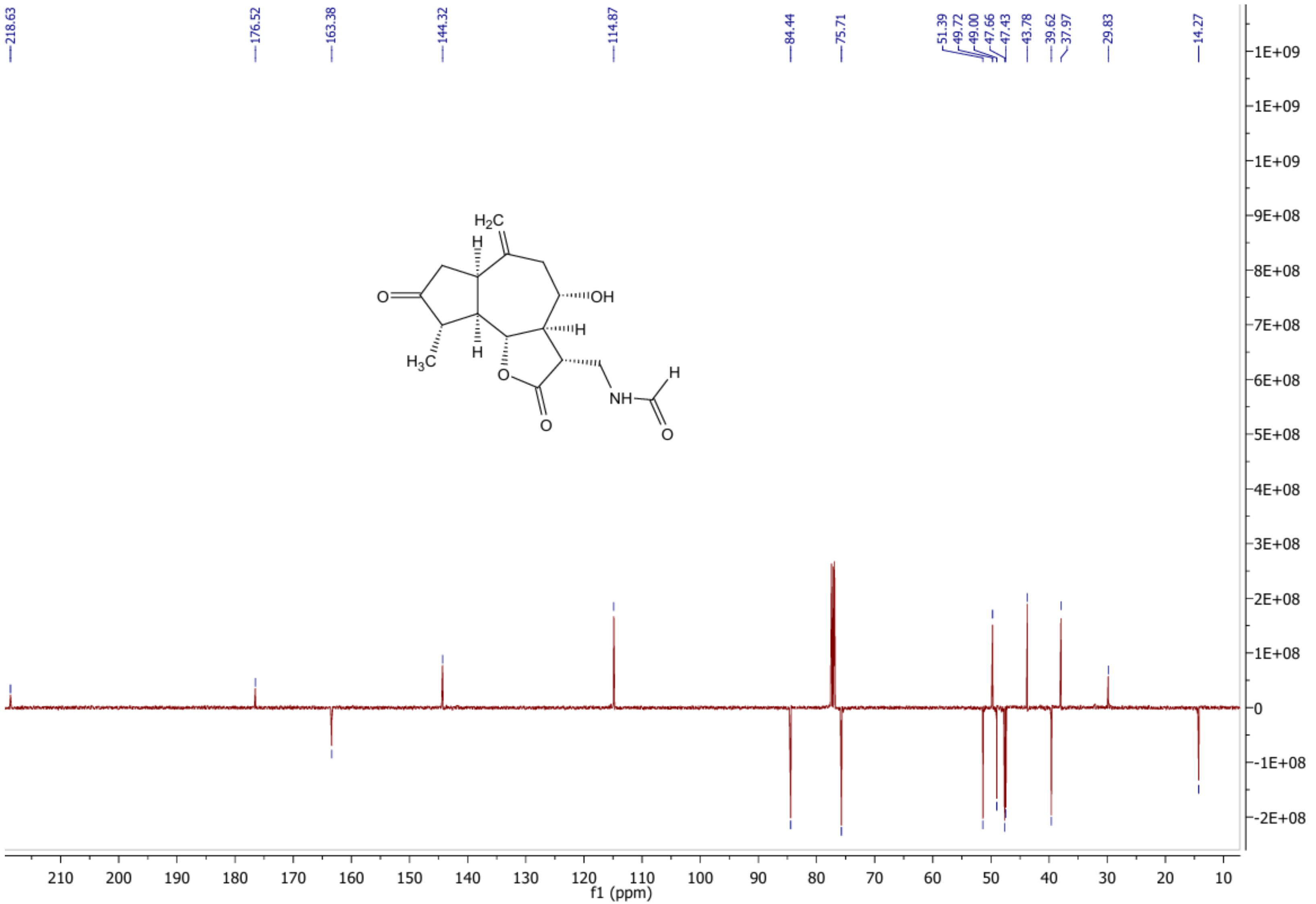

^{1}H-NMR of *(3S,3aR,4S,6aR,9S,9aR,9bR)-3-((benzylthio)methyl)-4-hydroxy-9-methyl-6-methyleneoctahydroazuleno[4,5-b]furan-2,8(3H,9bH)-dione* **8**

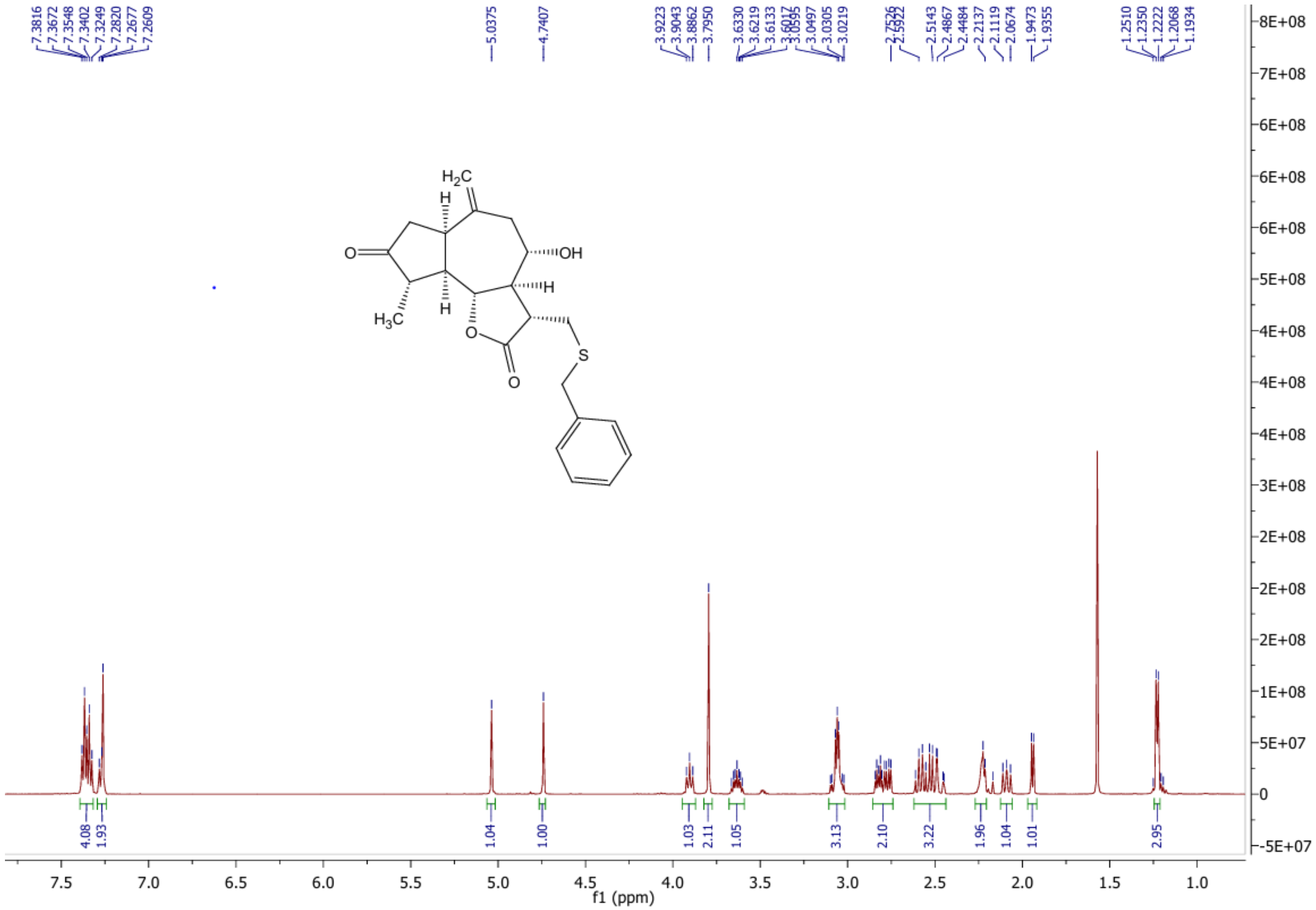

^{13}C-NMR of *(3S,3aR,4S,6aR,9S,9aR,9bR)-3-((benzylthio)methyl)-4-hydroxy-9-methyl-6-methyleneoctahydroazuleno[4,5-b]furan-2,8(3H,9bH)-dione* **8**

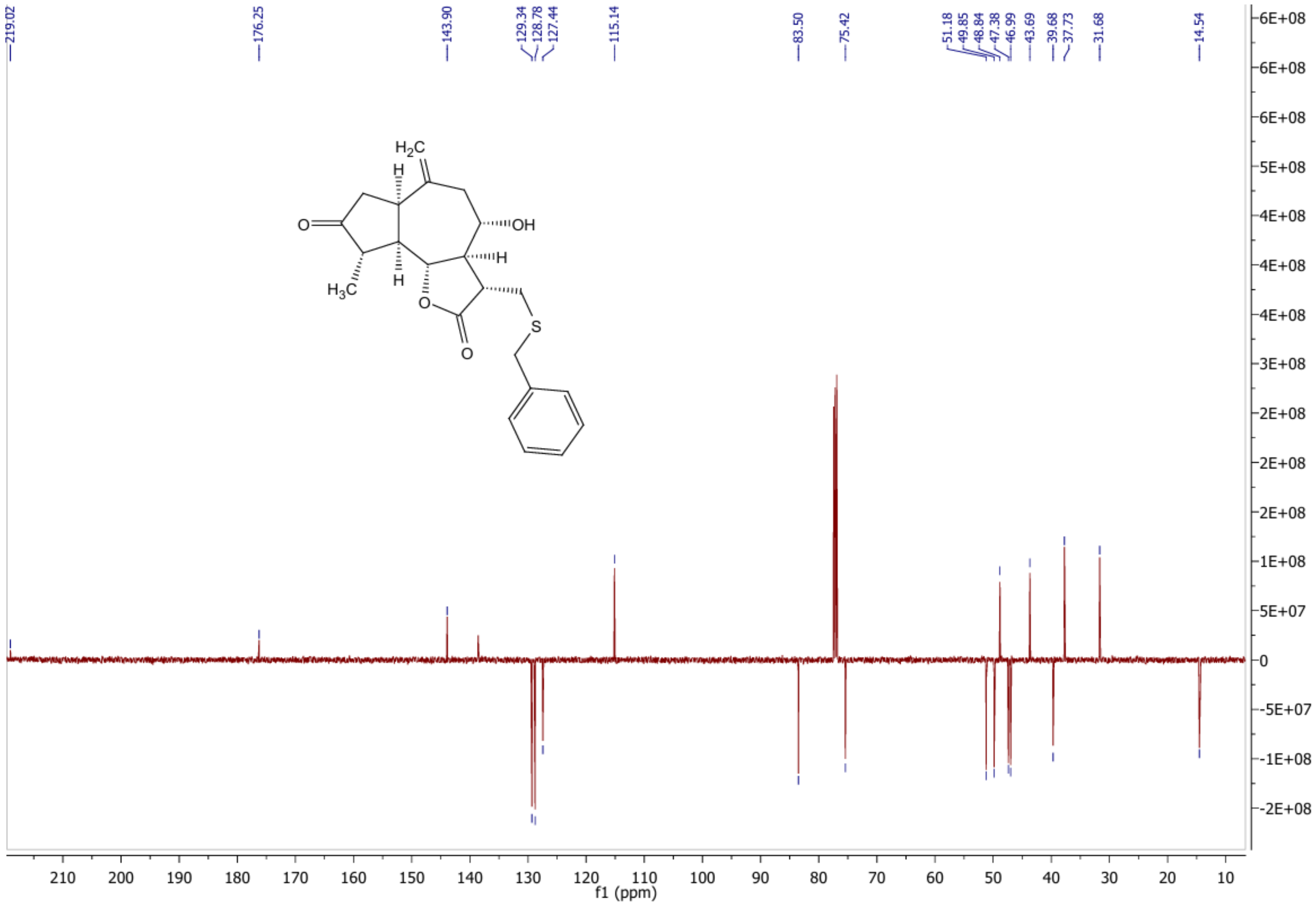

^{1}H-NMR of *(3S,3aR,4S,6aR,8R,9S,9aR,9bR)-3-((Benzylthio)methyl)-4,8-dihydroxy-9-methyl-6-methylenedecahydroazuleno[4,5-b]furan-2(9bH)-one* **9**

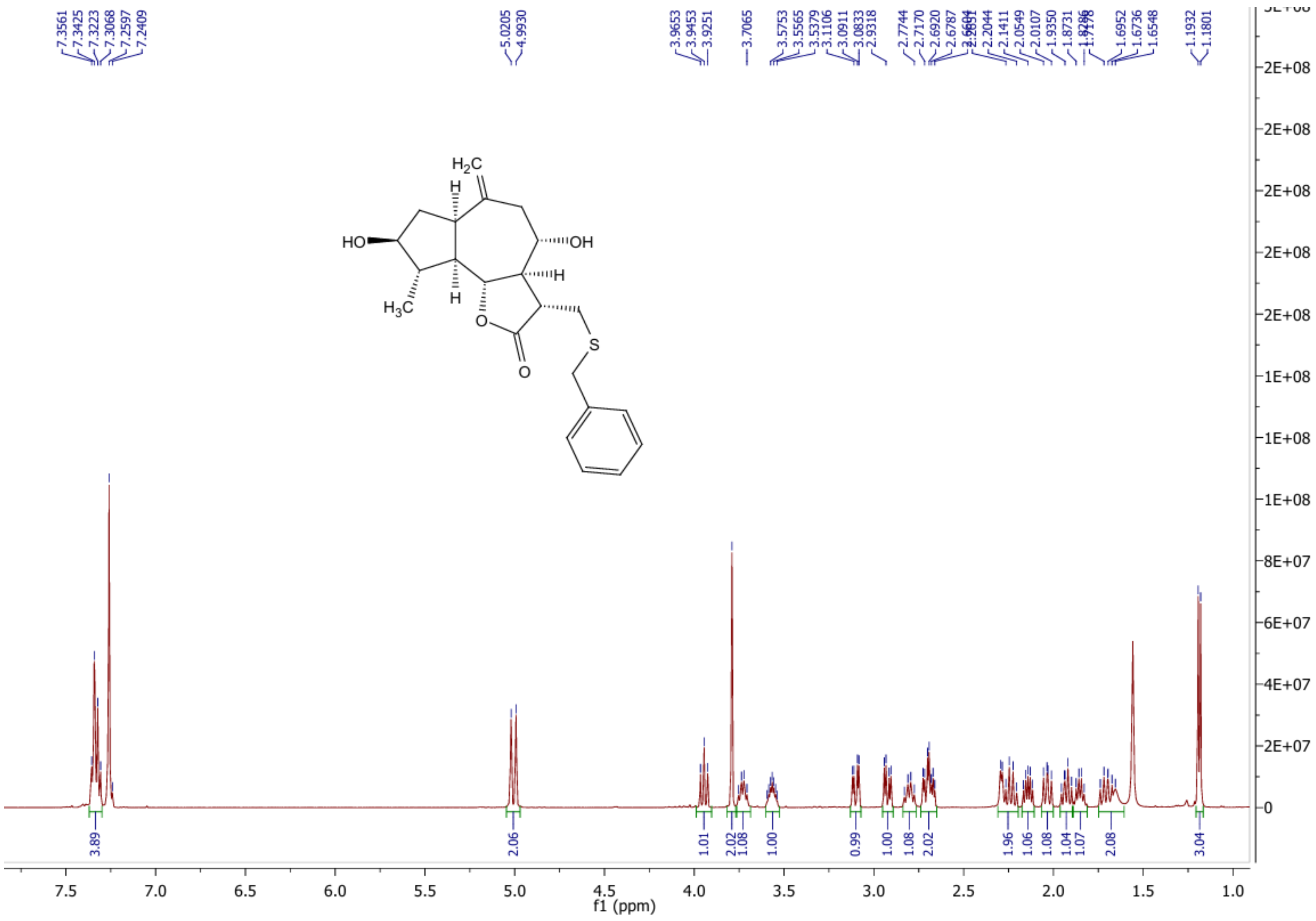

^{13}C-NMR of *(3S,3aR,4S,6aR,8R,9S,9aR,9bR)-3-((Benzylthio)methyl)-4,8-dihydroxy-9-methyl-6-methylenedecahydroazuleno[4,5-b]furan-2(9bH)-one* **9**

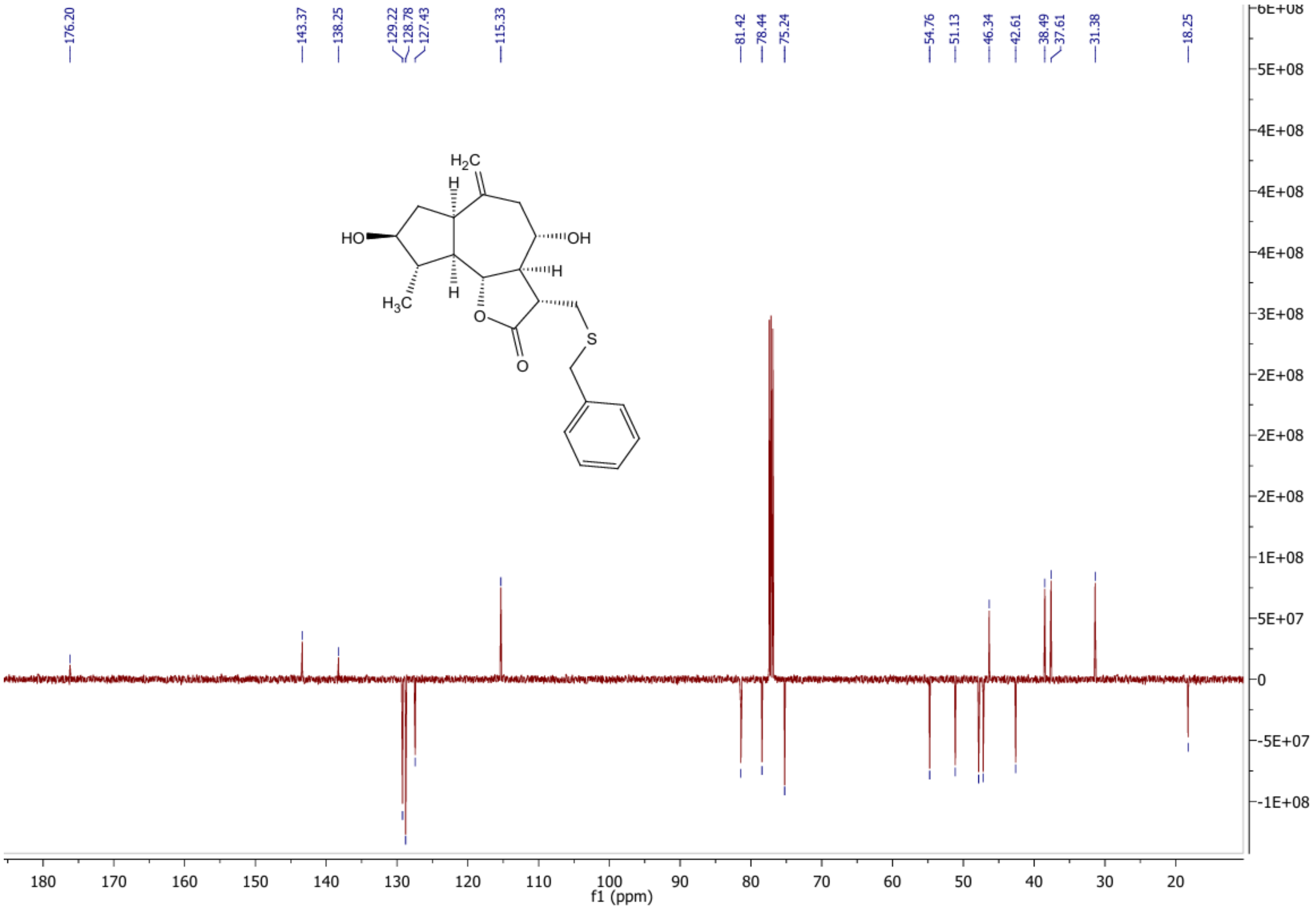

COSY spectrum of **9**

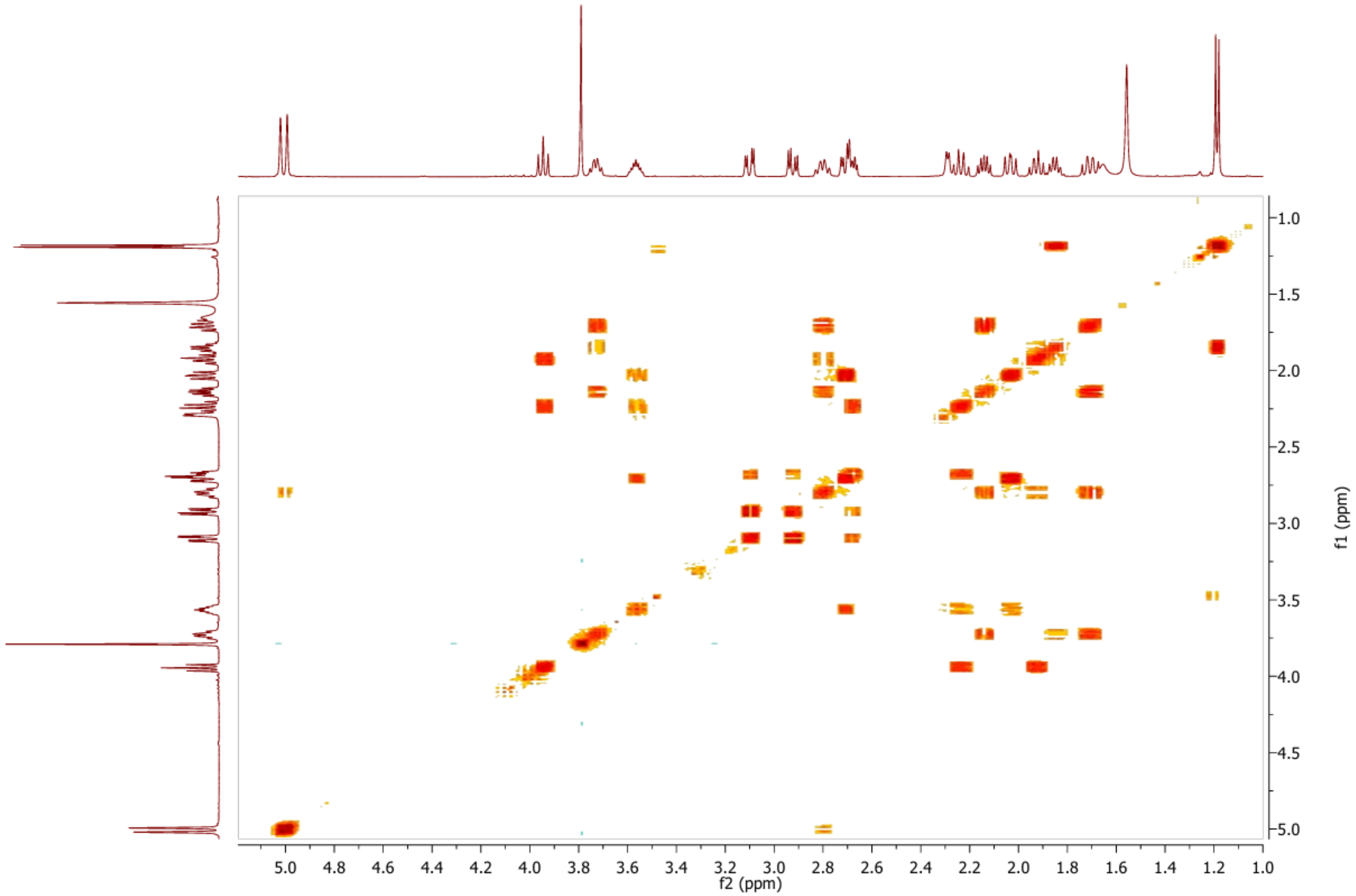

NOESY spectrum of **9**

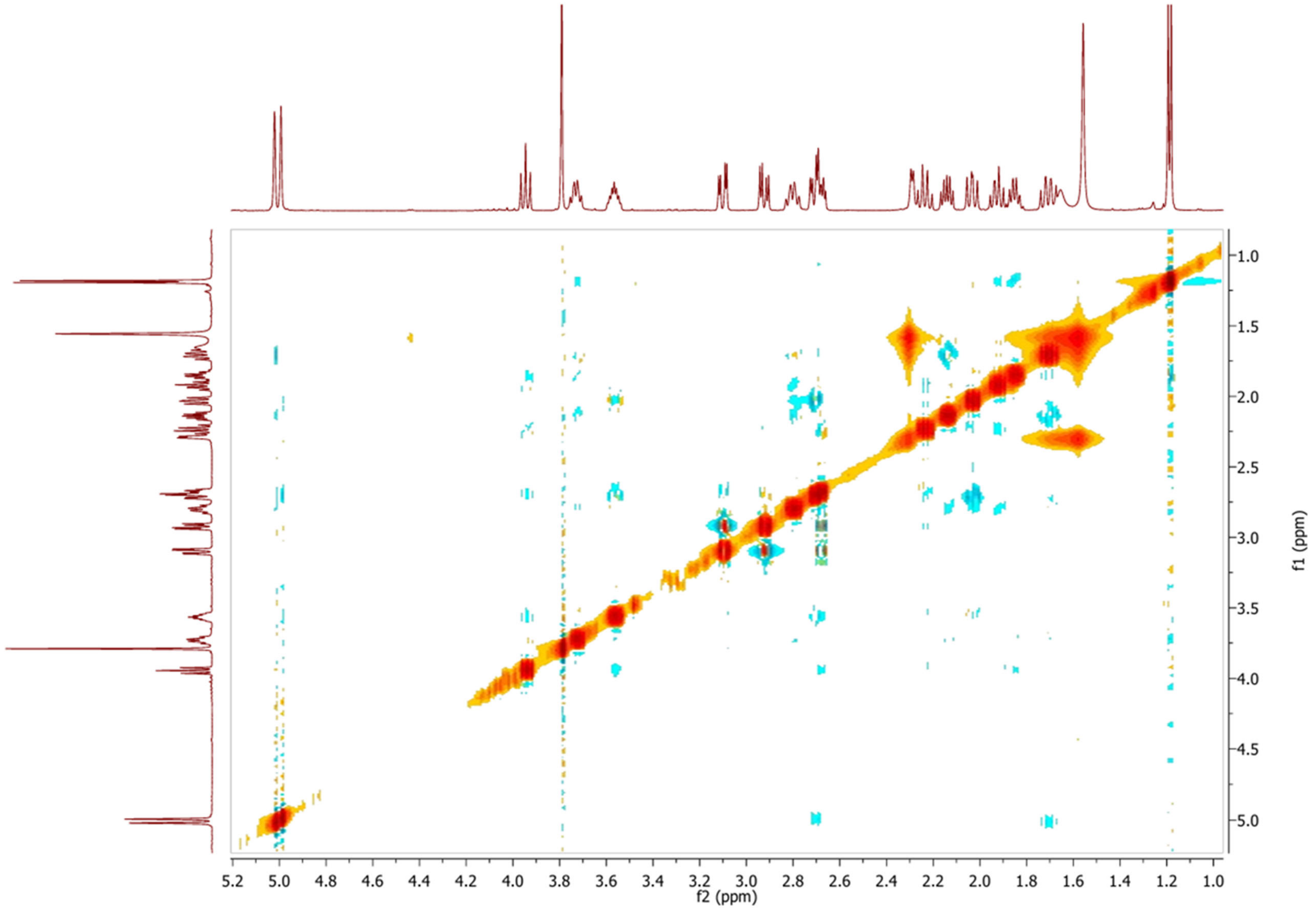

HSQC spectrum of **9**

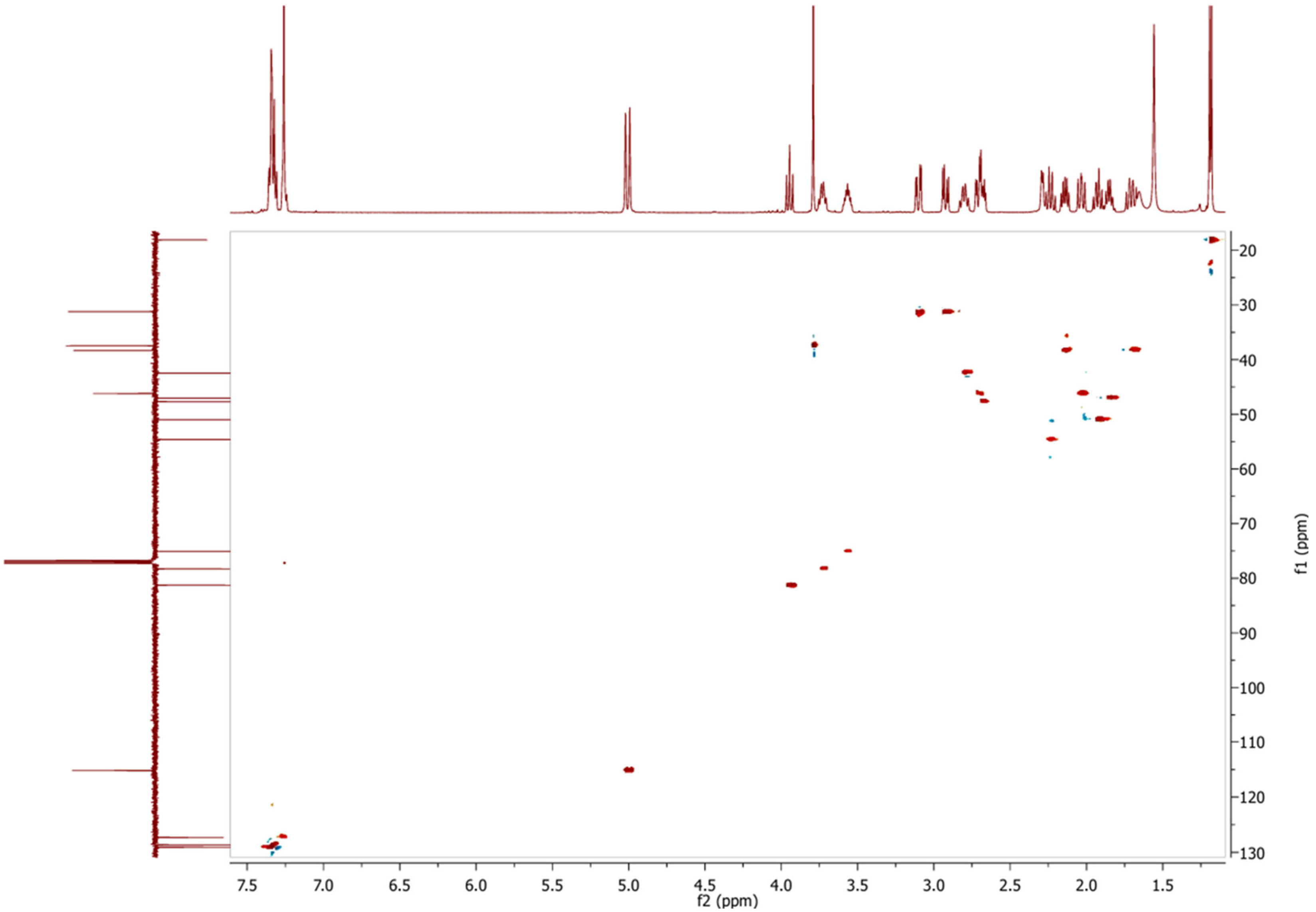

HMBC of compound **9**

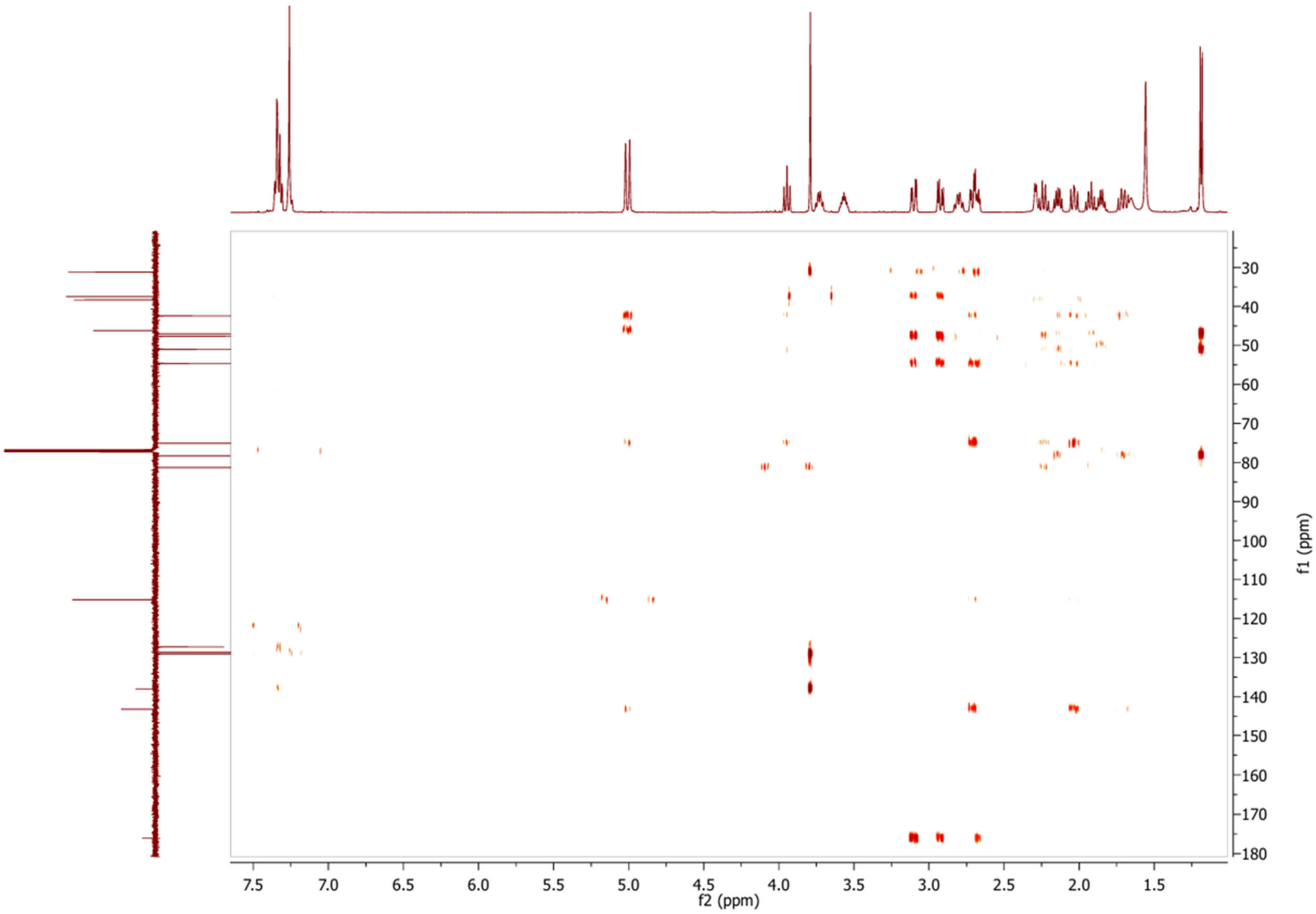

^{1}H-NMR of *(3R,3aR,4S,6aR,9S,9aR,9bR)-4-hydroxy-3-(methoxymethyl)-9-methyl-6-methyleneoctahydroazuleno[4,5-b]furan-2,8(3H,9bH)-dione* **10**

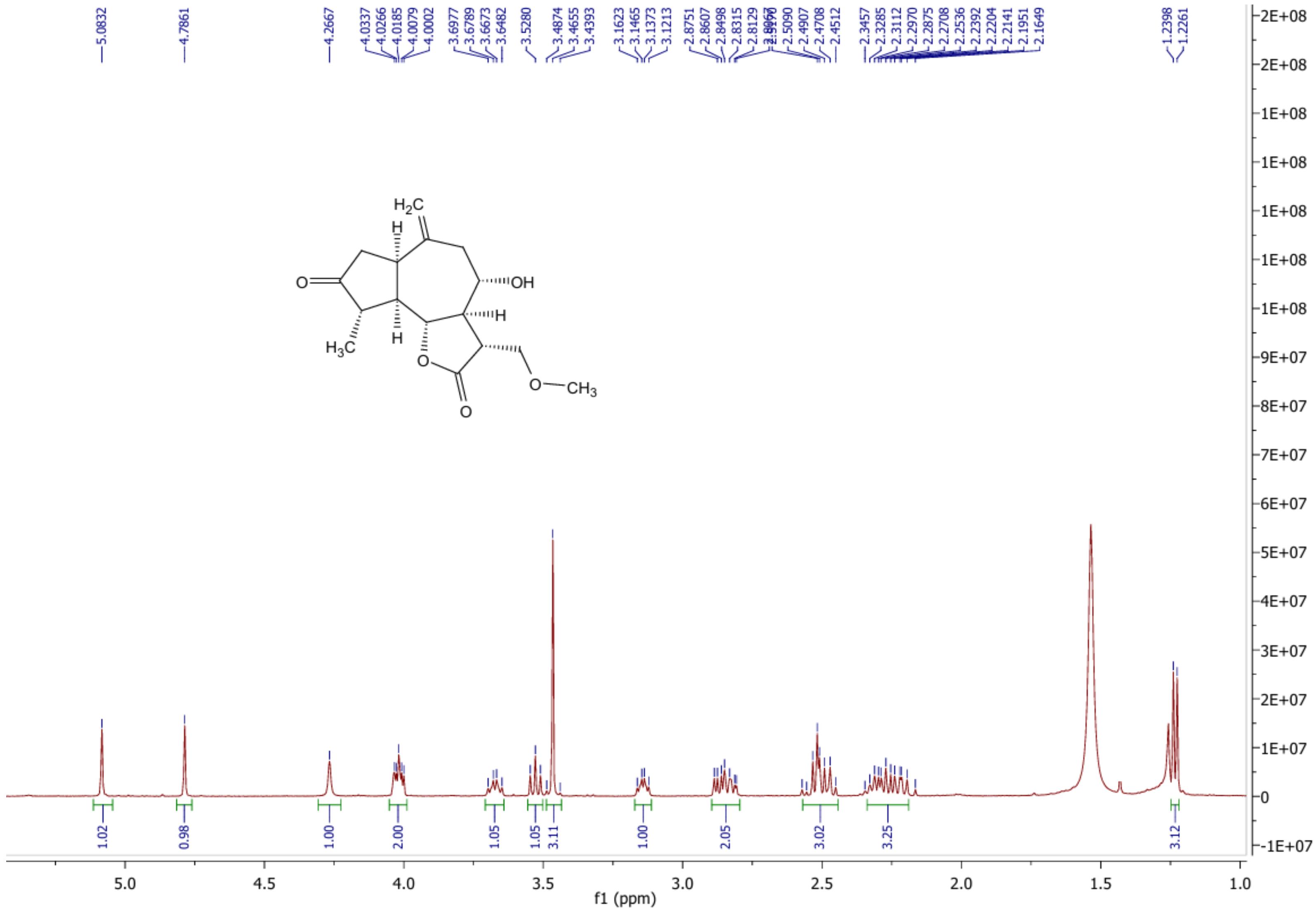

^{13}C-NMR of *(3R,3aR,4S,6aR,9S,9aR,9bR)-4-hydroxy-3-(methoxymethyl)-9-methyl-6-methyleneoctahydroazuleno[4,5-b]furan-2,8(3H,9bH)-dione* **10**

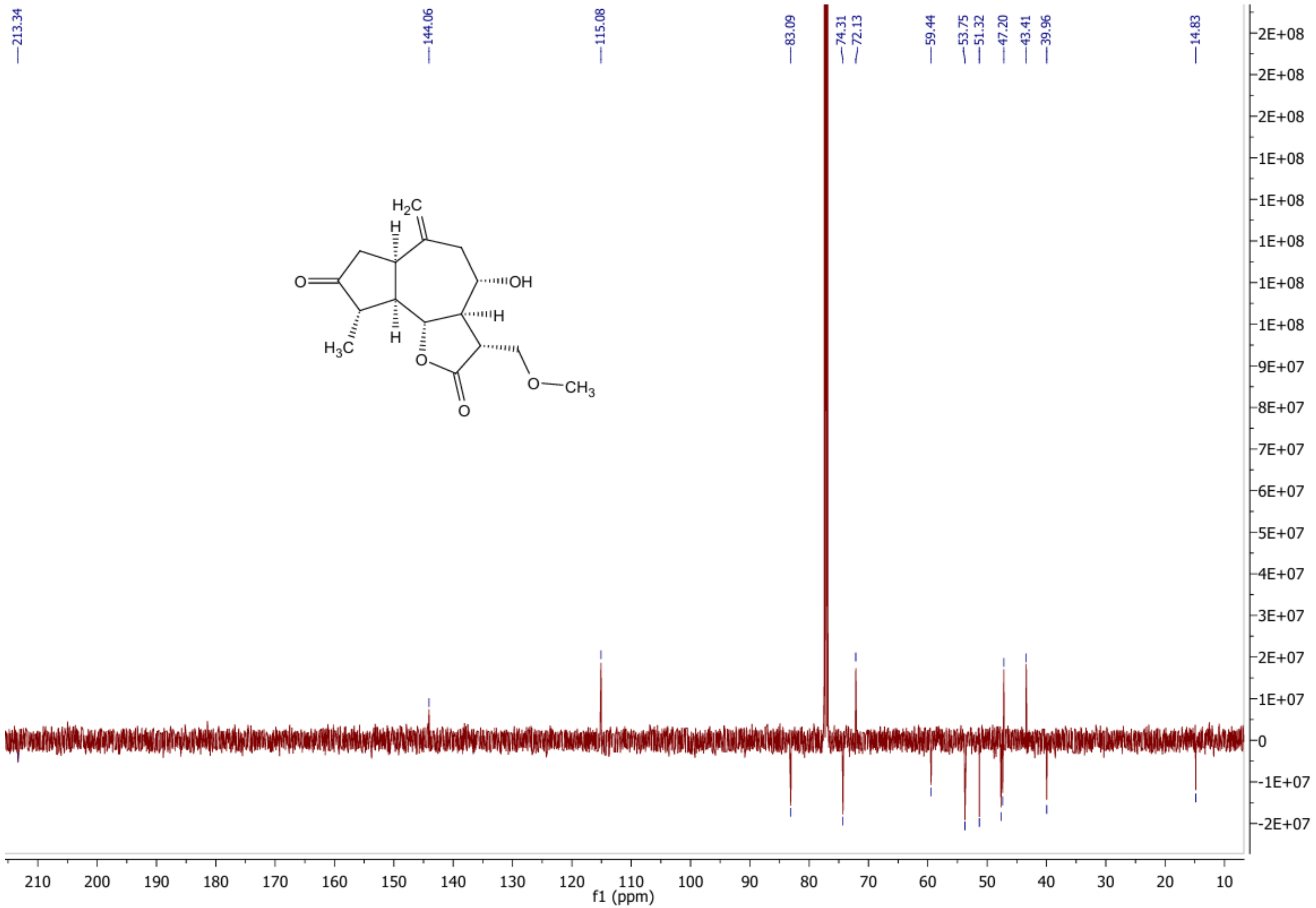

^{1}H-NMR *(3R,3aR,4S,6aR,9S,9aR,9bR)-3-((1H-imidazol-1-yl)methyl)-4-hydroxy-9-methyl-6-methyleneoctahydroazuleno[4,5-b]furan-2,8(3H,9bH)-dione* **11a**

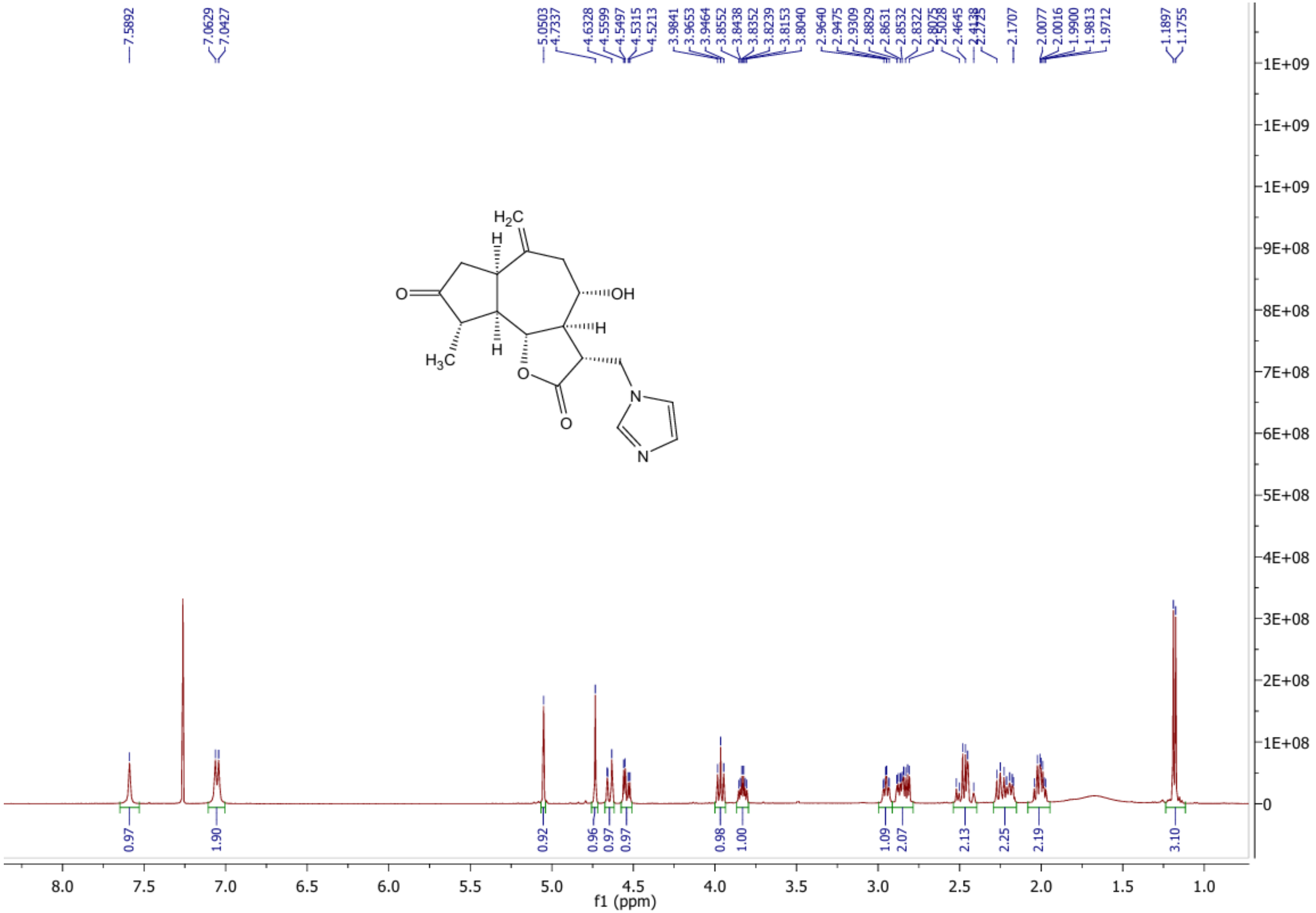

^{13}C-NMR of *(3R,3aR,4S,6aR,9S,9aR,9bR)-3-((1H-imidazol-1-yl)methyl)-4-hydroxy-9-methyl-6-methyleneoctahydroazuleno[4,5-b]furan-2,8(3H,9bH)-dione* **11a**

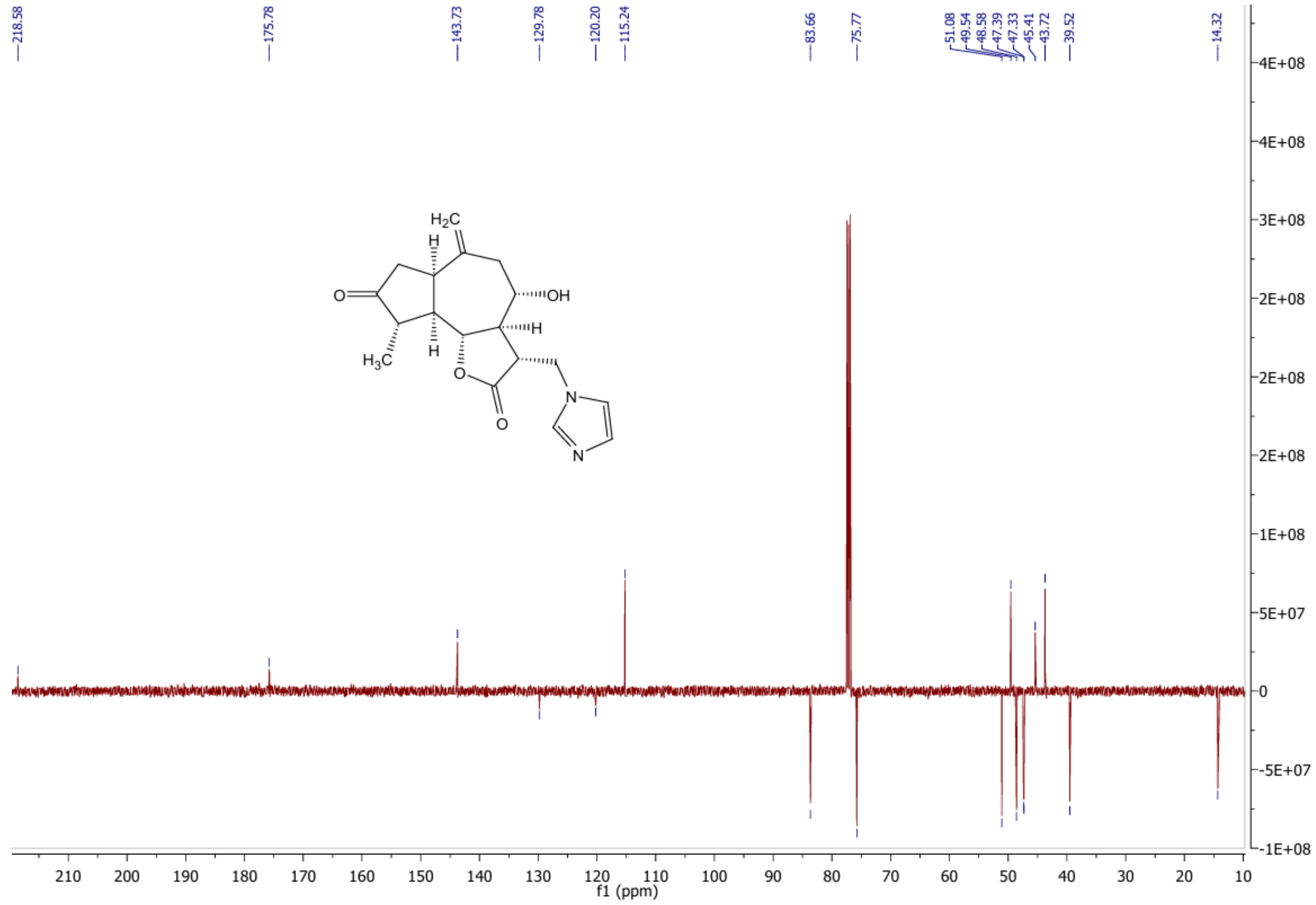

^{1}H-NMR of *(3R,3aR,4S,6aR,9S,9aR,9bR)-3-((1H-pyrazol-1-yl)methyl)-4-hydroxy-9-methyl-6-methyleneoctahydroazuleno[4,5-b]furan-2,8(3H,9bH)-dione* **11b**

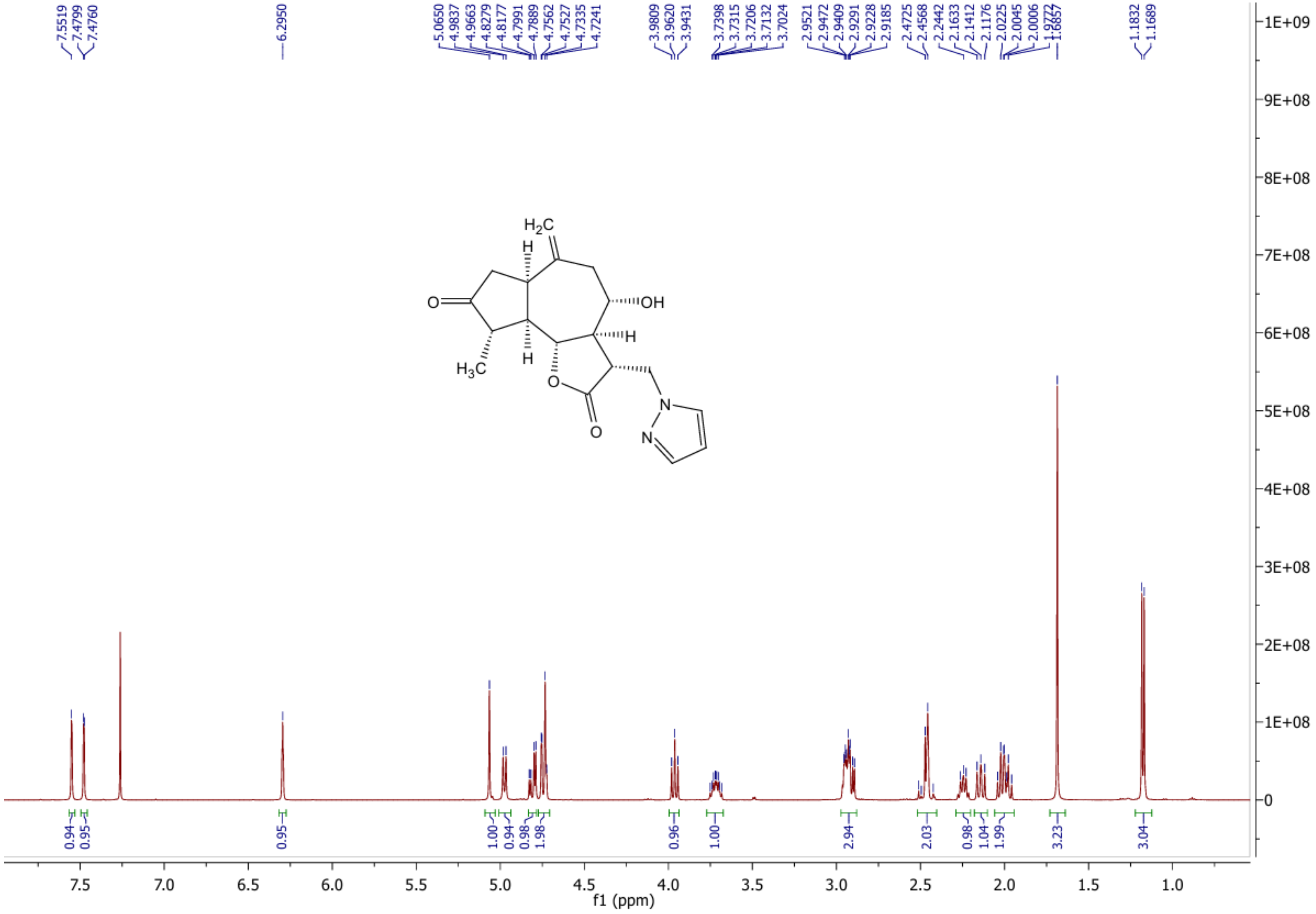

^{13}C-NMR of *(3R,3aR,4S,6aR,9S,9aR,9bR)-3-((1H-pyrazol-1-yl)methyl)-4-hydroxy-9-methyl-6-methyleneoctahydroazuleno[4,5-b]furan-2,8(3H,9bH)-dione* **11b**

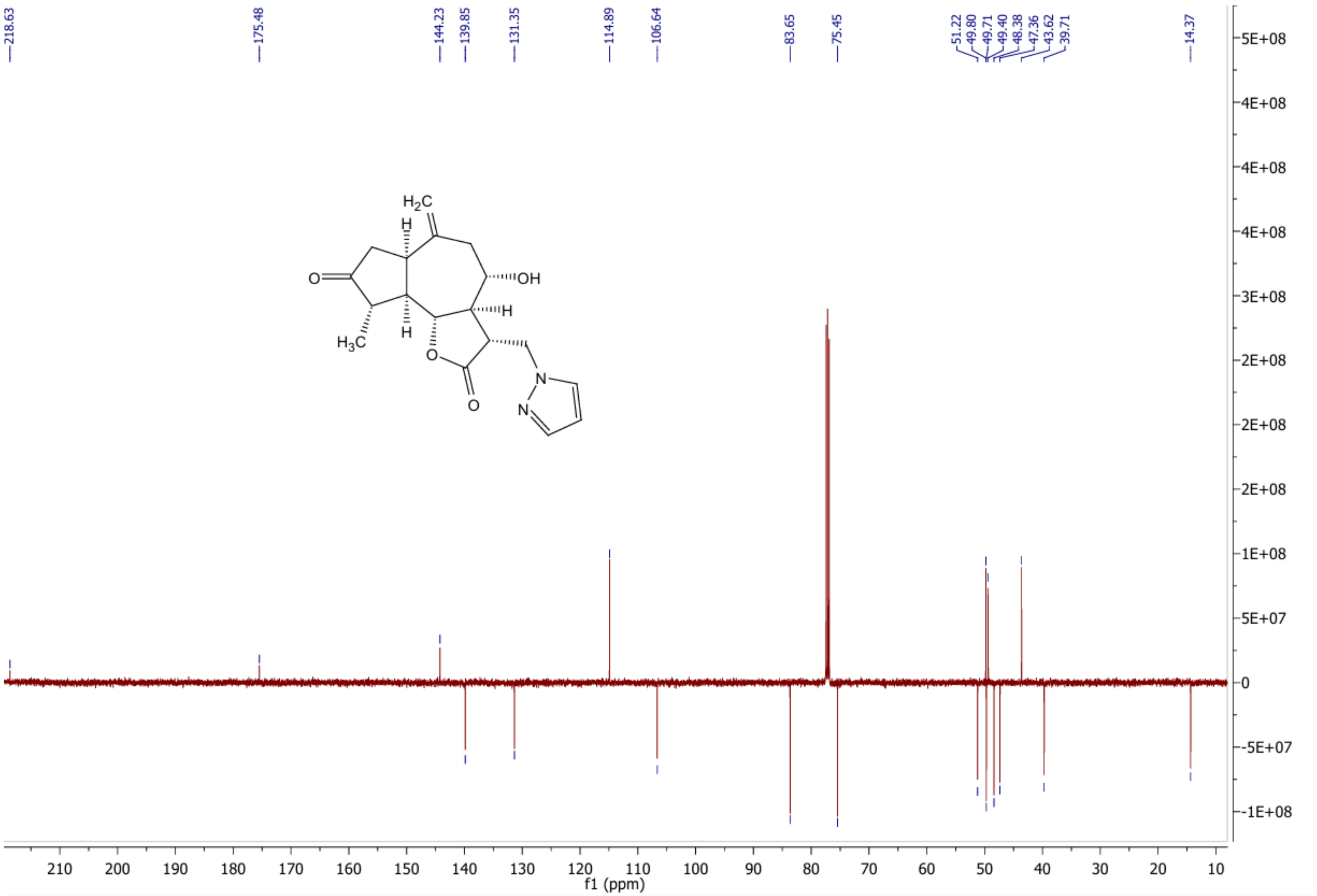

^{1}H-NMR of *(3R,3aR,4S,6aR,9S,9aR,9bR)-3-((1H-1,2,4-triazol-1-yl)methyl)-4-hydroxy-9-methyl-6-methyleneoctahydroazuleno[4,5-b]furan-2,8(3H,9bH)-dione* **11c**

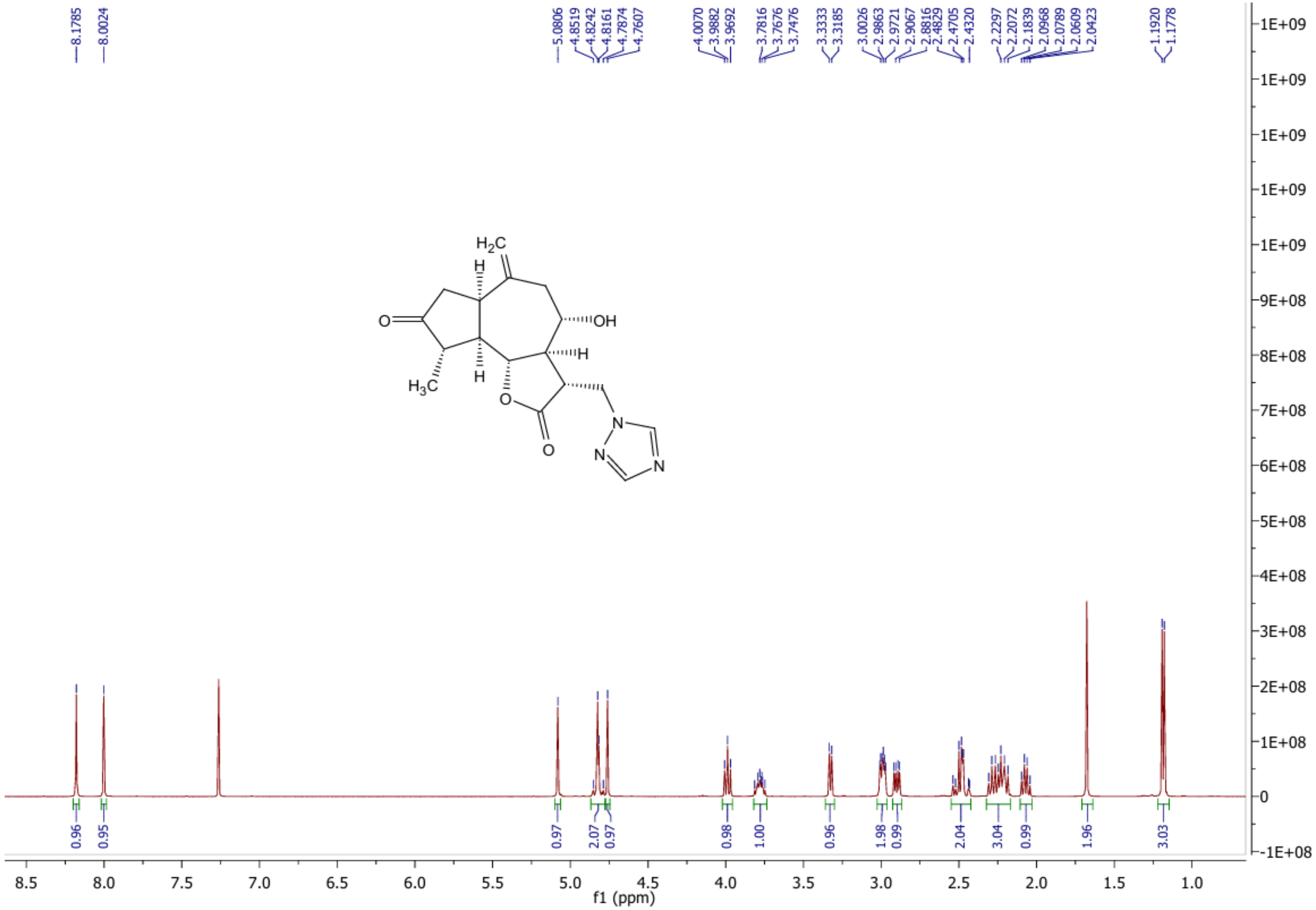

^{13}C-NMR of *(3R,3aR,4S,6aR,9S,9aR,9bR)-3-((1H-1,2,4-triazol-1-yl)methyl)-4-hydroxy-9-methyl-6-methyleneoctahydroazuleno[4,5-b]furan-2,8(3H,9bH)-dione* **11c**

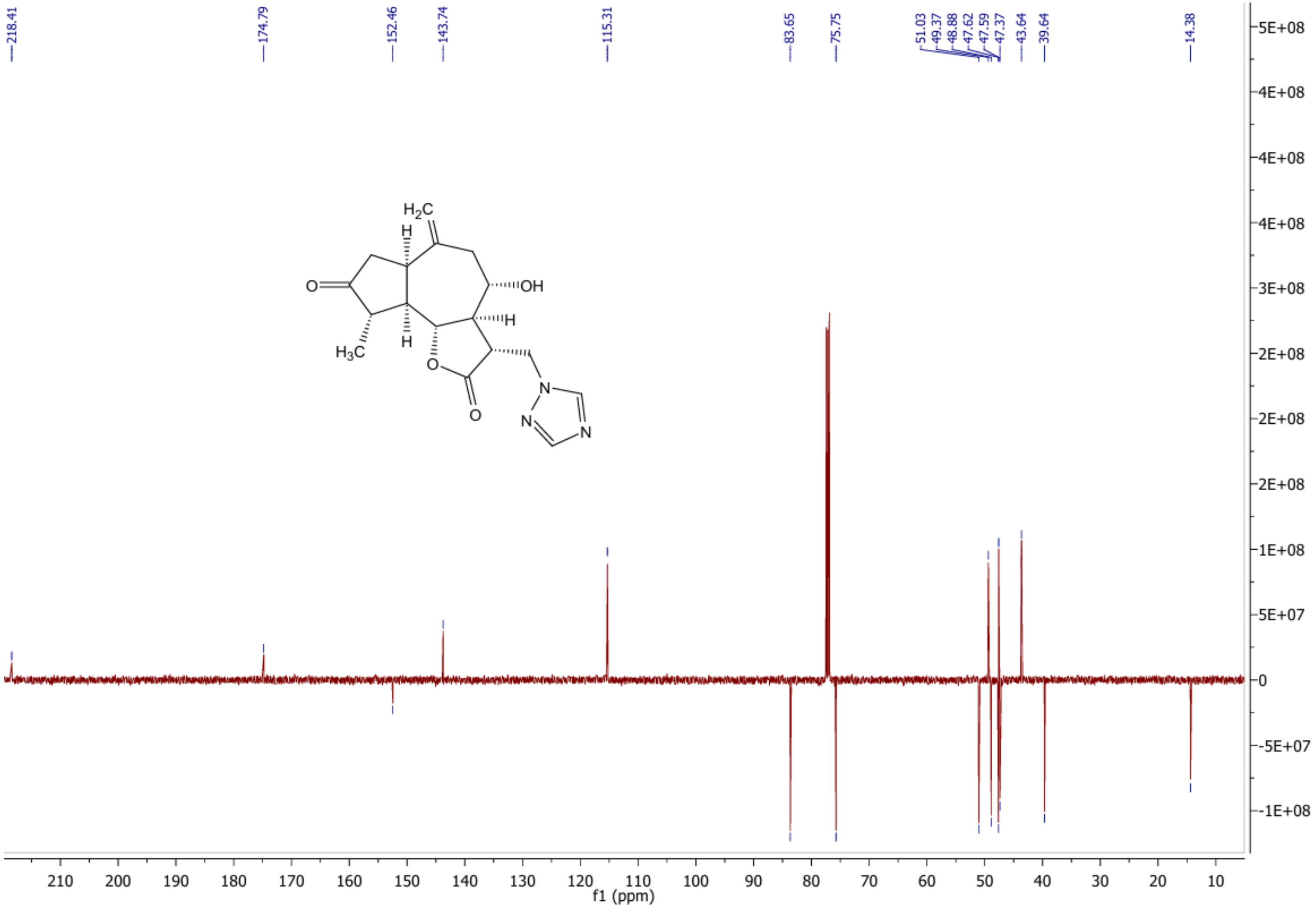

^{1}H-NMR of *(3R,3aR,4S,6aR,9S,9aR,9bR)-3-((1H-1,2,3-triazol-1-yl)methyl)-4-hydroxy-9-methyl-6-methyleneoctahydroazuleno[4,5-b]furan-2,8(3H,9bH)-dione* **11d**

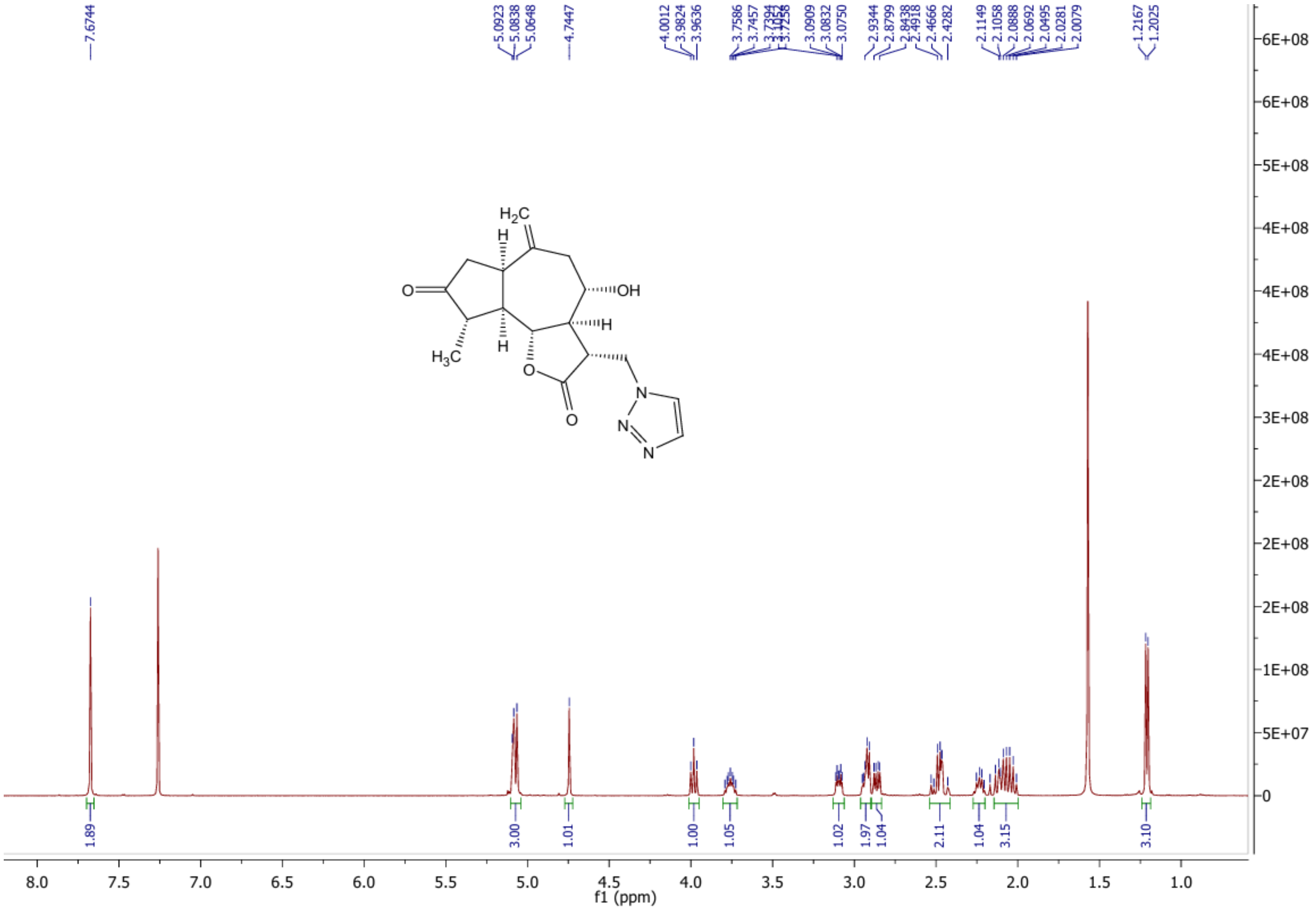

^{13}C-NMR of *(3R,3aR,4S,6aR,9S,9aR,9bR)-3-((1H-1,2,3-triazol-1-yl)methyl)-4-hydroxy-9-methyl-6-methyleneoctahydroazuleno[4,5-b]furan-2,8(3H,9bH)-dione* **11d**

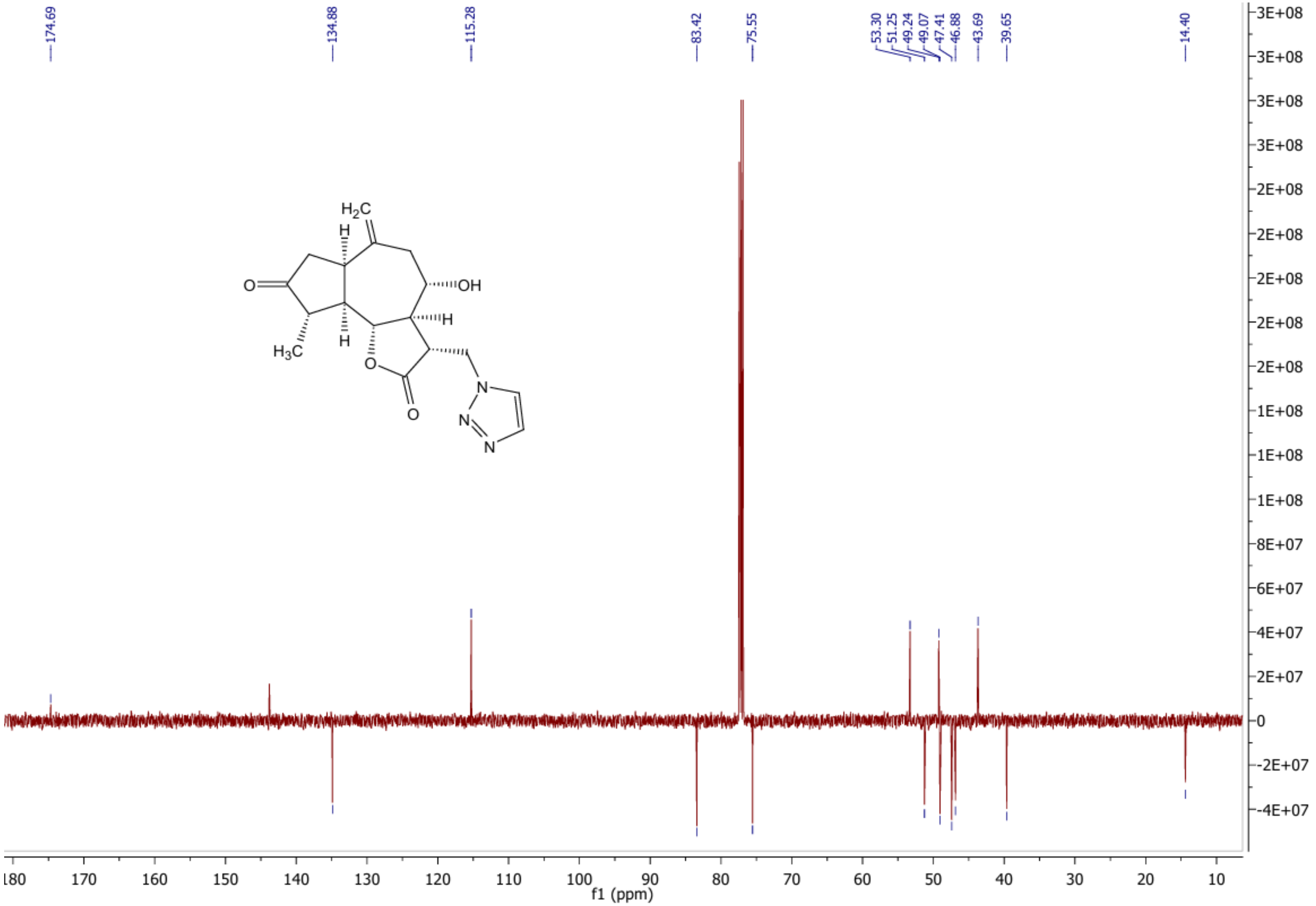

^{1}H-NMR of *(3R,3aR,4S,6aR,9S,9aR,9bR)-3-((1H-indol-1-yl)methyl)-4-hydroxy-9-methyl-6-methyleneoctahydroazuleno[4,5-b]furan-2,8(3H,9bH)-dione* **11e**

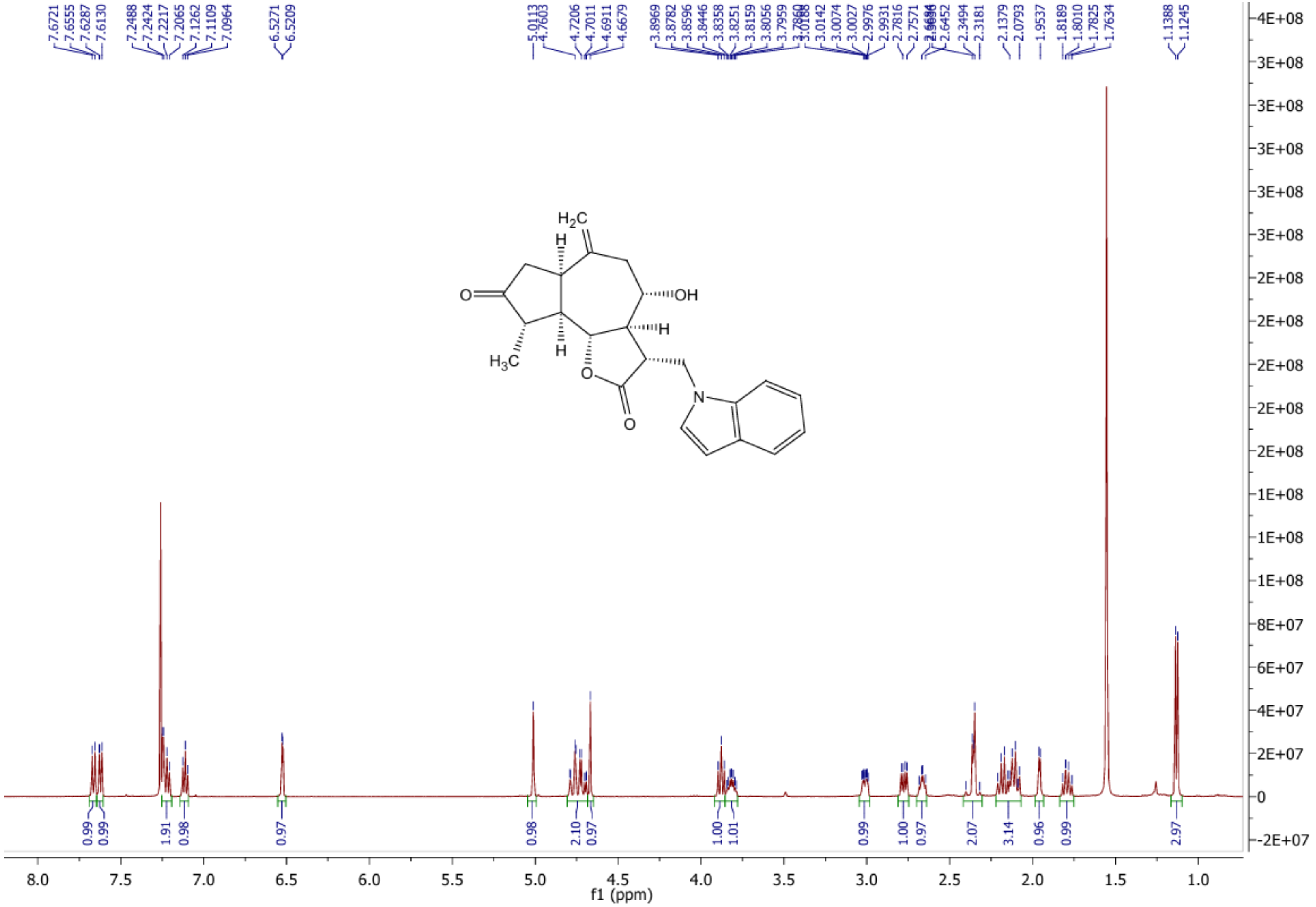

^{13}C-NMR of *(3R,3aR,4S,6aR,9S,9aR,9bR)-3-((1H-indol-1-yl)methyl)-4-hydroxy-9-methyl-6-methyleneoctahydroazuleno[4,5-b]furan-2,8(3H,9bH)-dione* **11e**

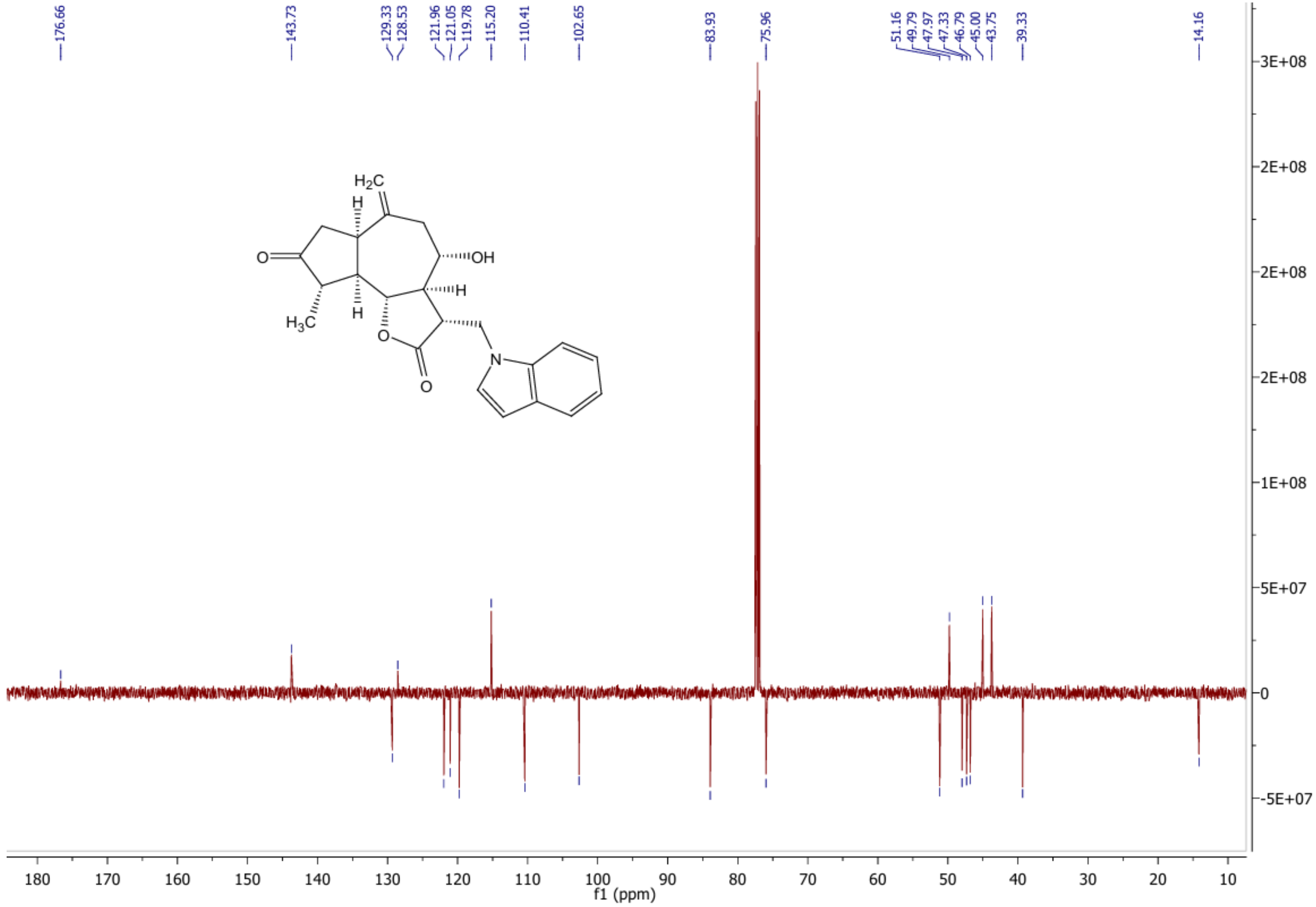

^{1}H-NMR of *(3R,3aR,4S,6aR,9S,9aR,9bR)-3-((1H-benzo[d]imidazol-1-yl)methyl)-4-hydroxy-9-methyl-6-methyleneoctahydroazuleno[4,5-b]furan-2,8(3H,9bH)-dione* **11f**

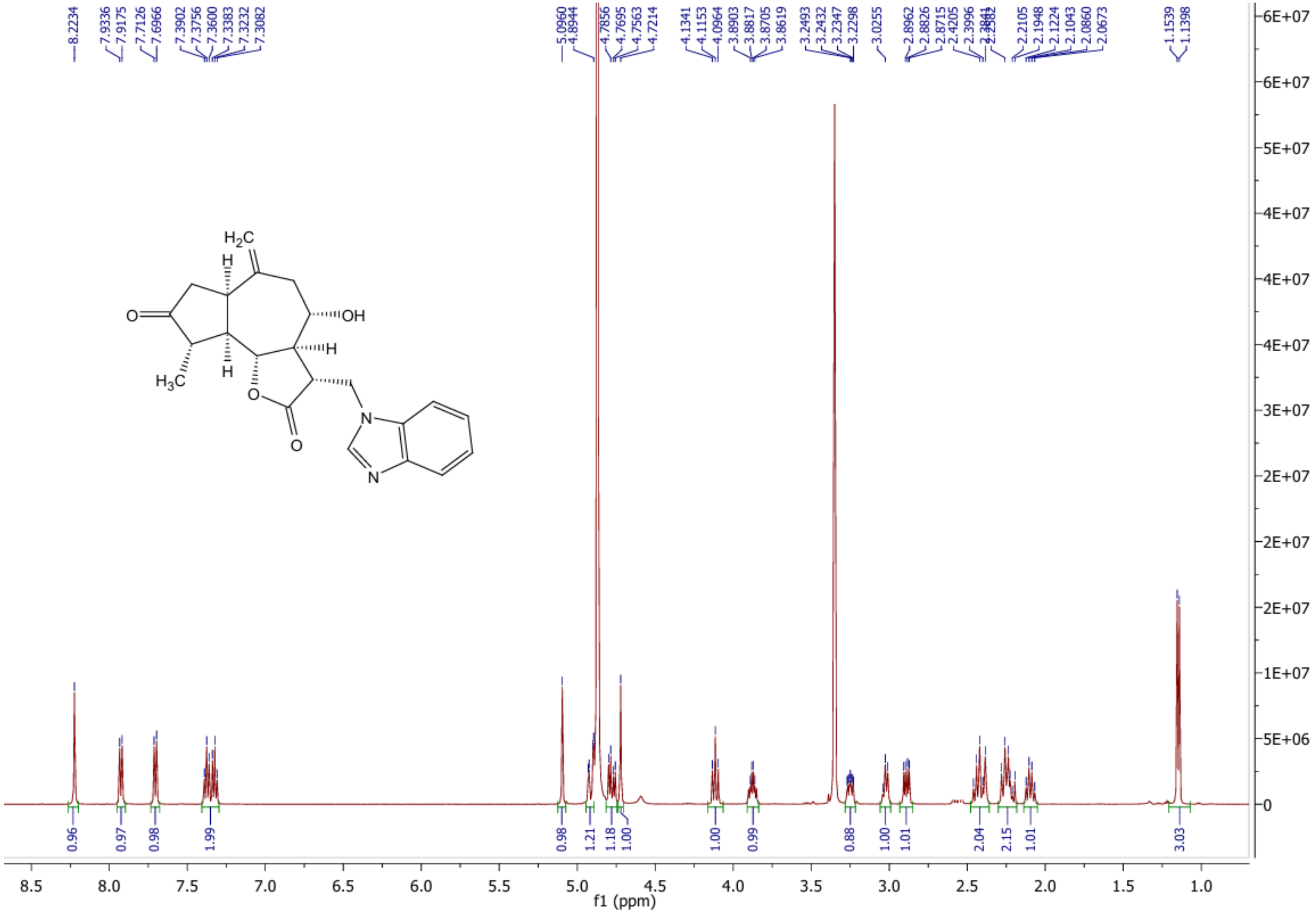

^{13}C-NMR of *(3R,3aR,4S,6aR,9S,9aR,9bR)-3-((1H-benzo[d]imidazol-1-yl)methyl)-4-hydroxy-9-methyl-6-methyleneoctahydroazuleno[4,5-b]furan-2,8(3H,9bH)-dione* **11f**

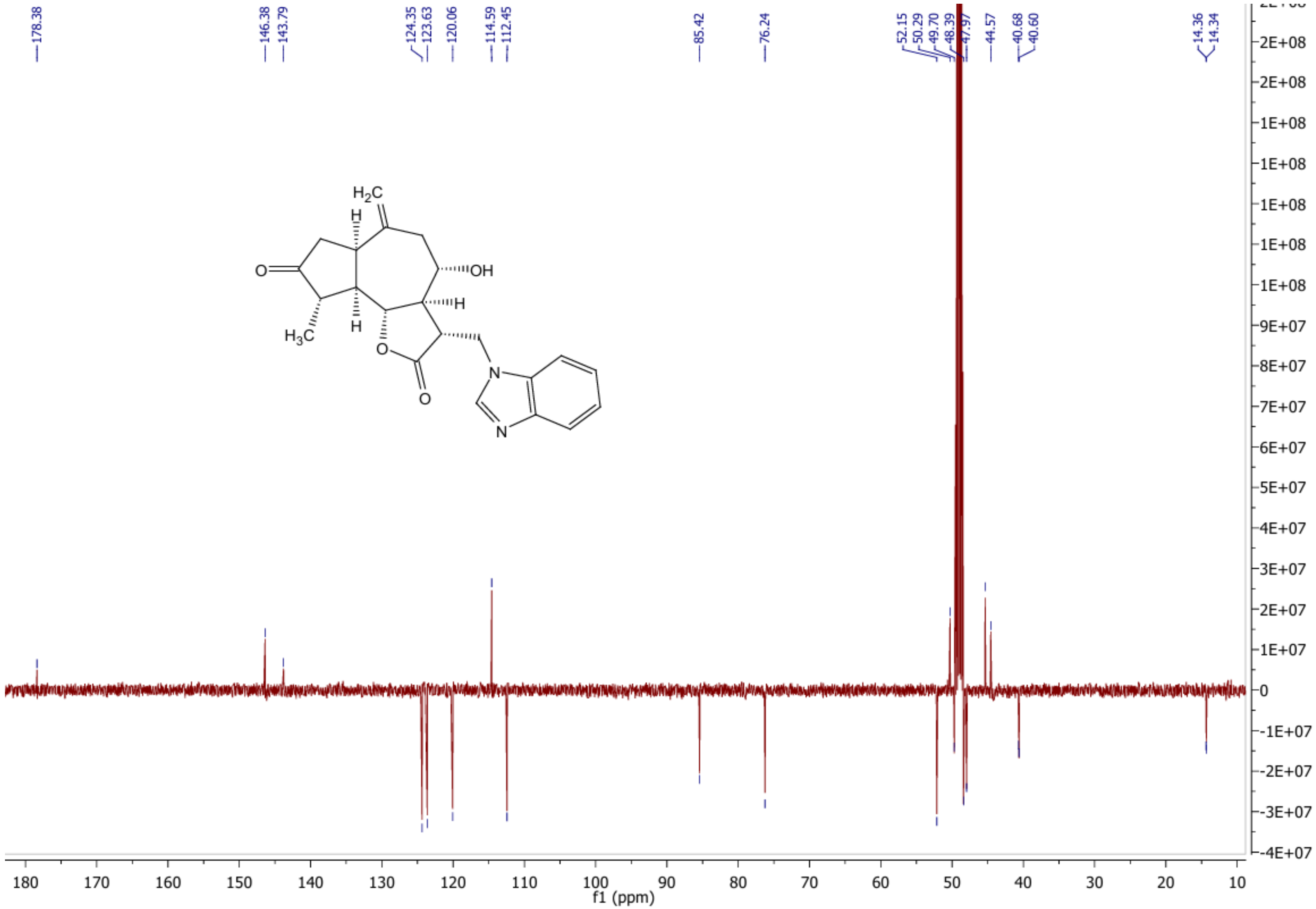

^{1}H-NMR of (*3R,3aR,4S,6aR,9S,9aR,9bR)-3-((1H-indazol-1-yl)methyl)-4-hydroxy-9-methyl-6-methyleneoctahydroazuleno[4,5-b]furan-2,8(3H,9bH)-dione* **11g**

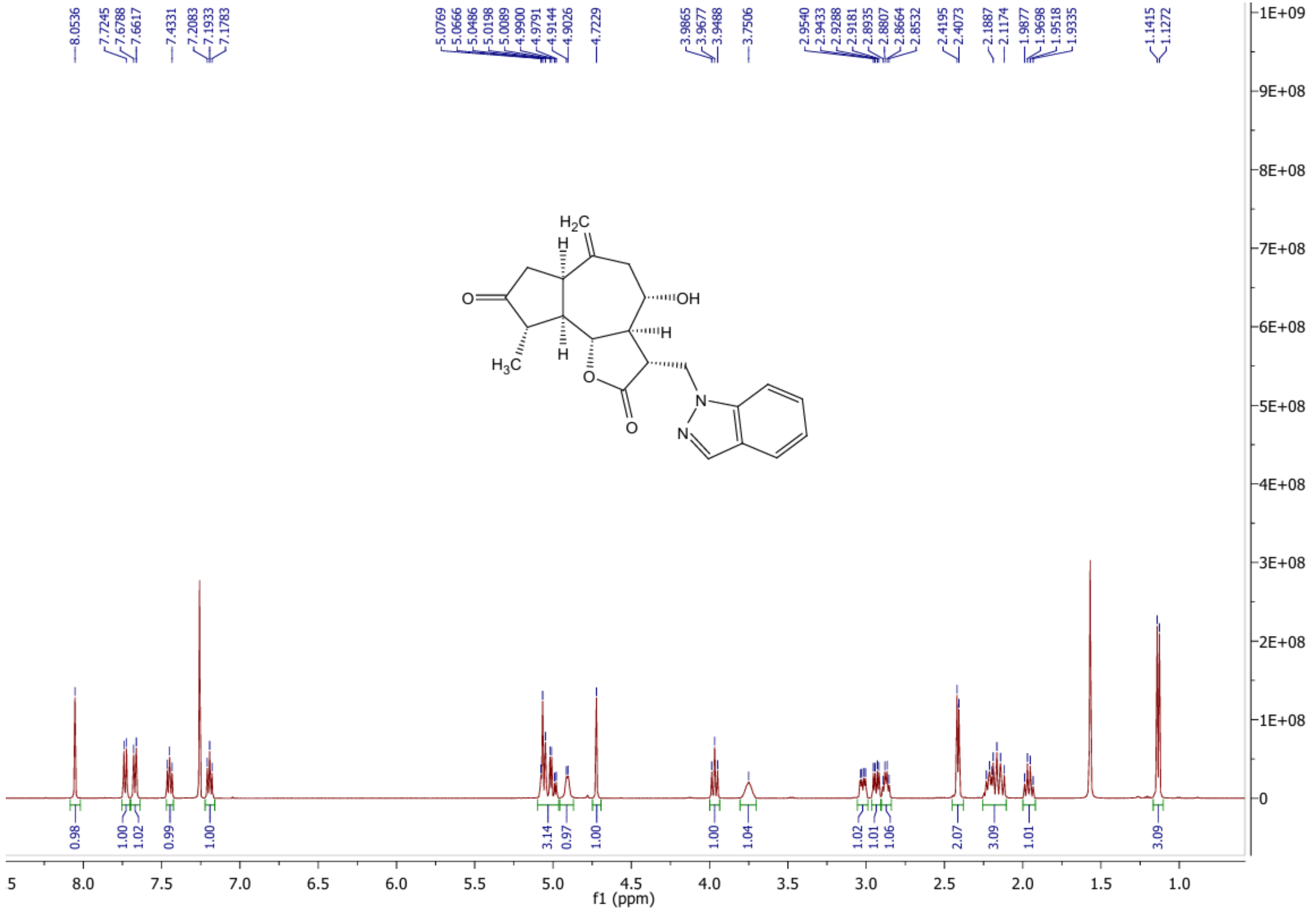

^{13}C-NMR of *(3R,3aR,4S,6aR,9S,9aR,9bR)-3-((1H-indazol-1-yl)methyl)-4-hydroxy-9-methyl-6-methyleneoctahydroazuleno[4,5-b]furan-2,8(3H,9bH)-dione* **11g**

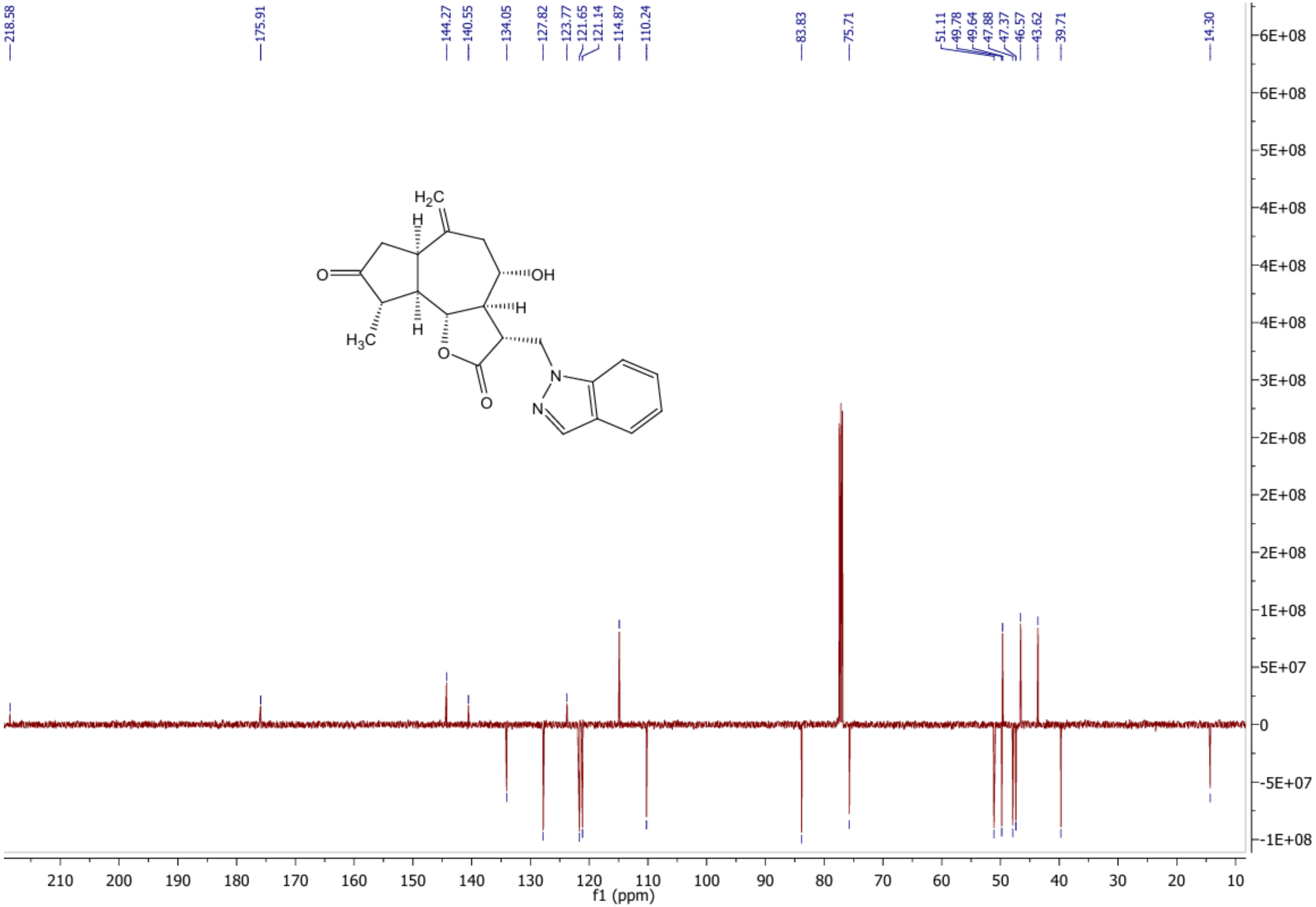

^{1}H-NMR of *(3R,3aR,4S,6aR,9S,9aR,9bR)-3-((1H-benzo[d][1,2,3]triazol-1-yl)methyl)-4-hydroxy-9-methyl-6-methyleneoctahydroazuleno[4,5-b]furan-2,8(3H,9bH)-dione* **11h**

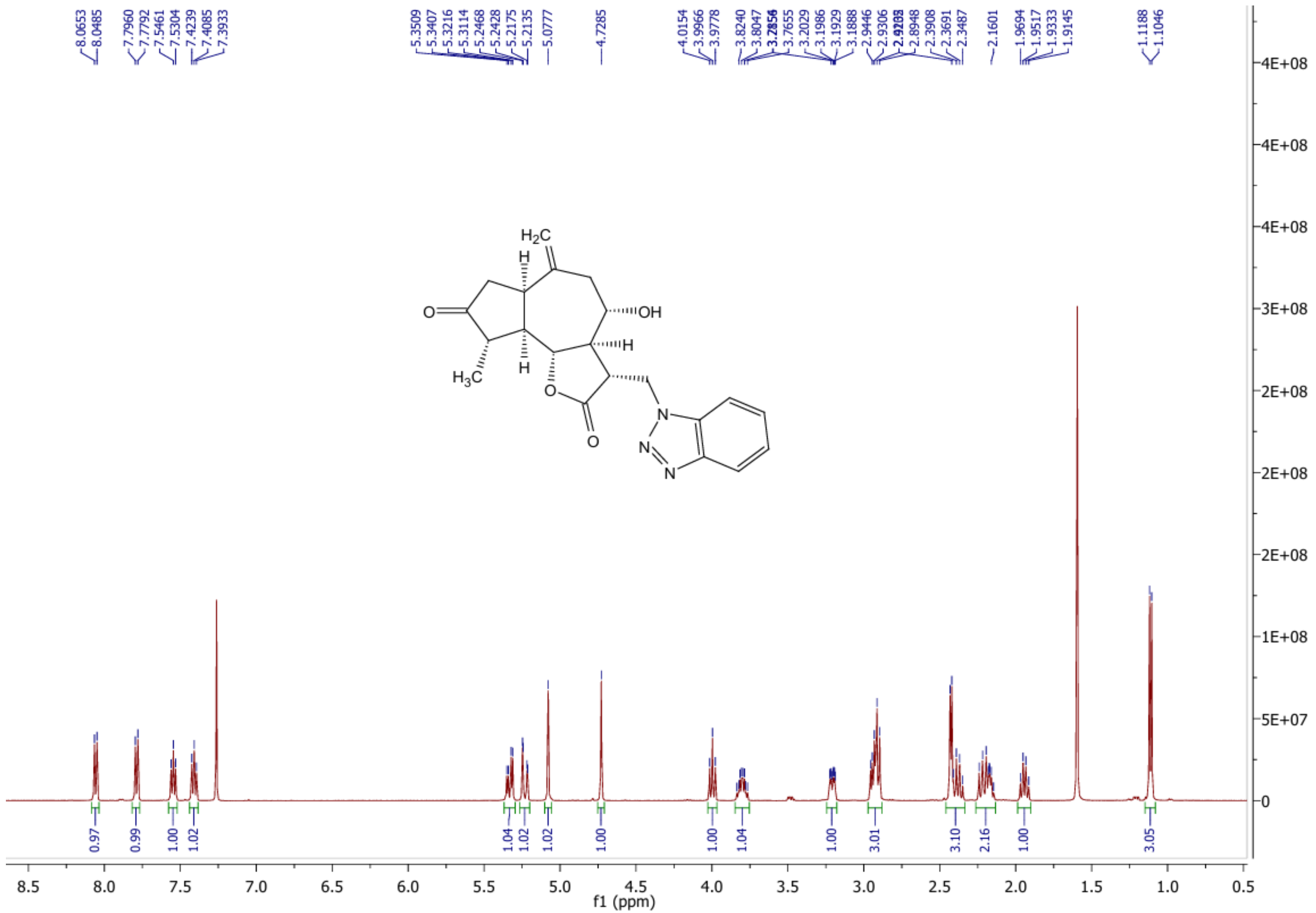

^{13}C-NMR of *(3R,3aR,4S,6aR,9S,9aR,9bR)-3-((1H-benzo[d][1,2,3]triazol-1-yl)methyl)-4-hydroxy-9-methyl-6-methyleneoctahydroazuleno[4,5-b]furan-2,8(3H,9bH)-dione* **11h**

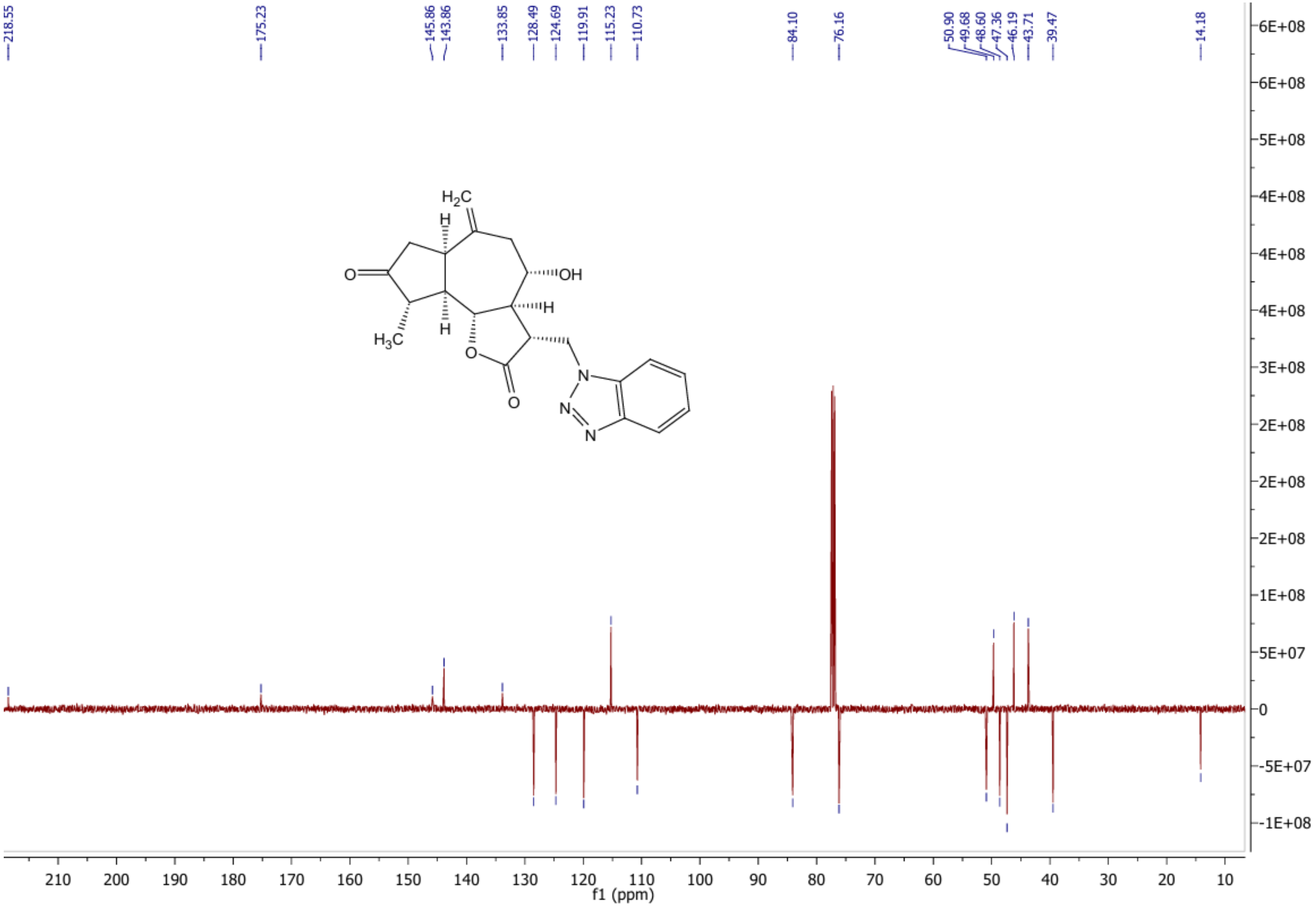

^{1}H- NMR of *(3aR,4S,6aR,8R,9S,9aR,9bR)-4,8-dihydroxy-9-methyl-3,6-dimethylenedecahydroazuleno[4,5-b]furan-2(9bH)-one* **12**

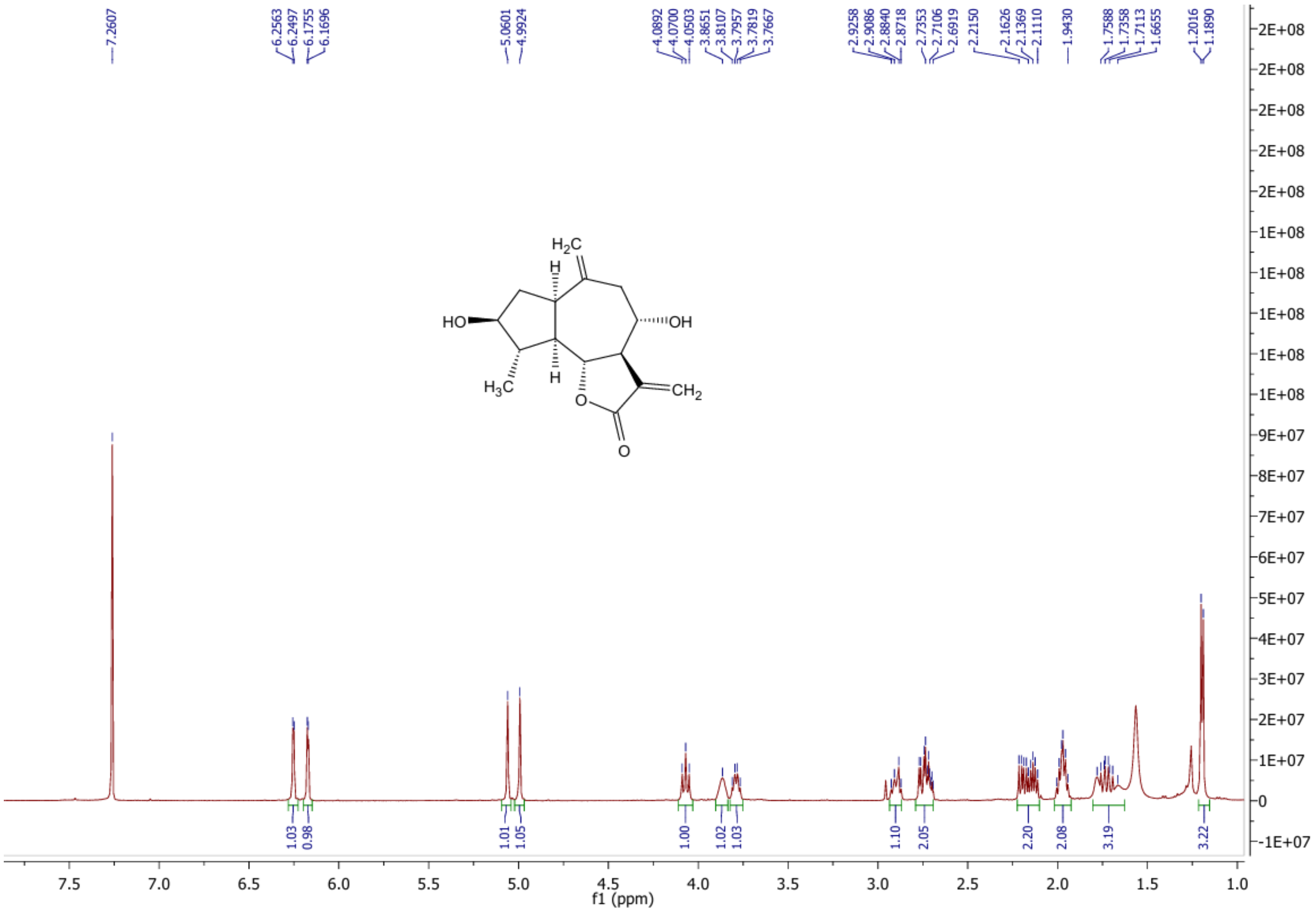

^{13}C-NMR of *(3aR,4S,6aR,8R,9S,9aR,9bR)-4,8-dihydroxy-9-methyl-3,6-dimethylenedecahydroazuleno[4,5-b]furan-2(9bH)-one* **12**

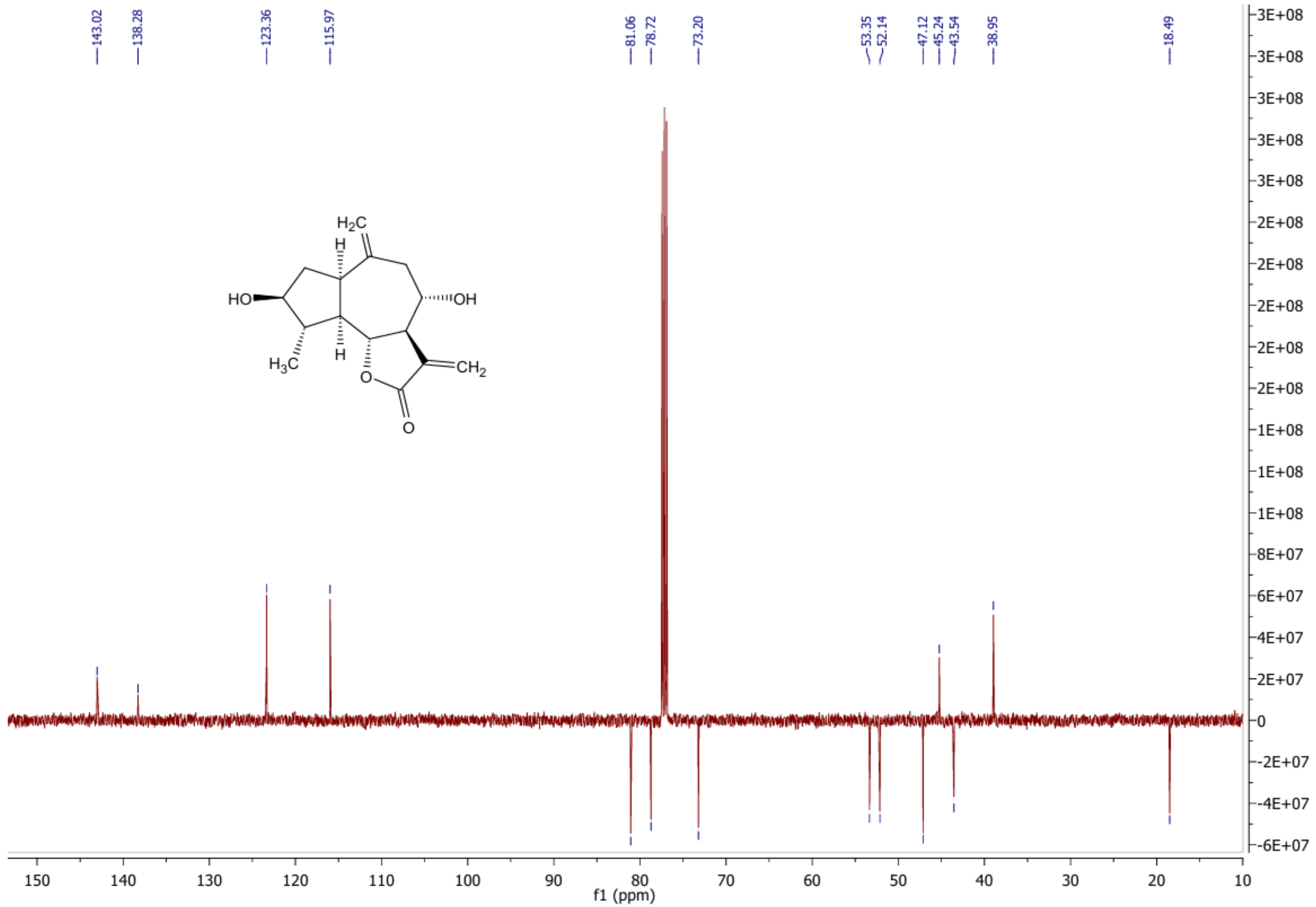

^{1}H-NMR of *(3aR,4S,6aR,8S,9S,9aR,9bR)-9-Methyl-3,6-dimethylene-2-oxododecahydroazuleno[4,5-b]furan-4,8-diyl diacetate* **13**

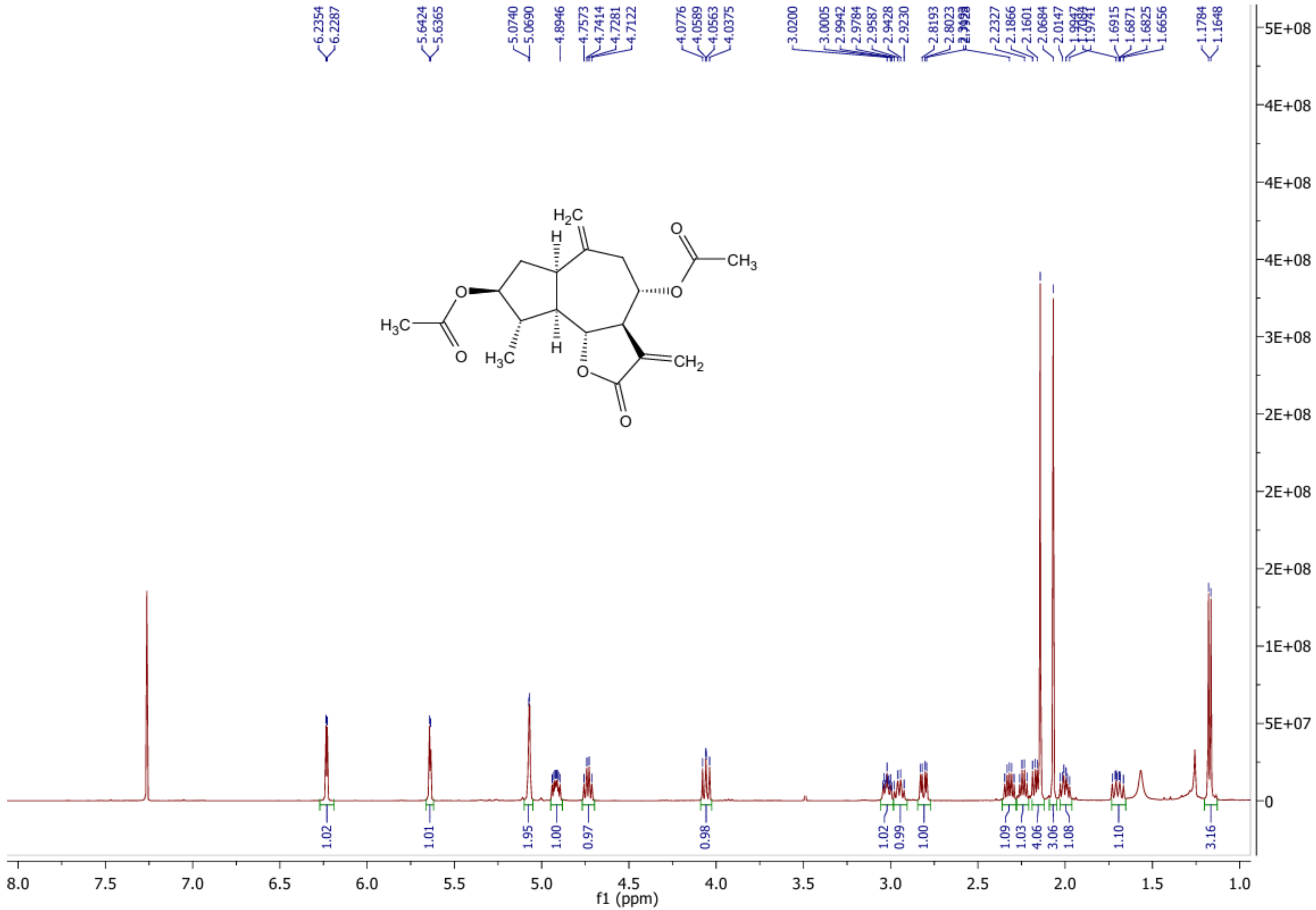

^{13}C-NMR of *(3aR,4S,6aR,8S,9S,9aR,9bR)-9-Methyl-3,6-dimethylene-2-oxododecahydroazuleno[4,5-b]furan-4,8-diyl diacetate* **13**

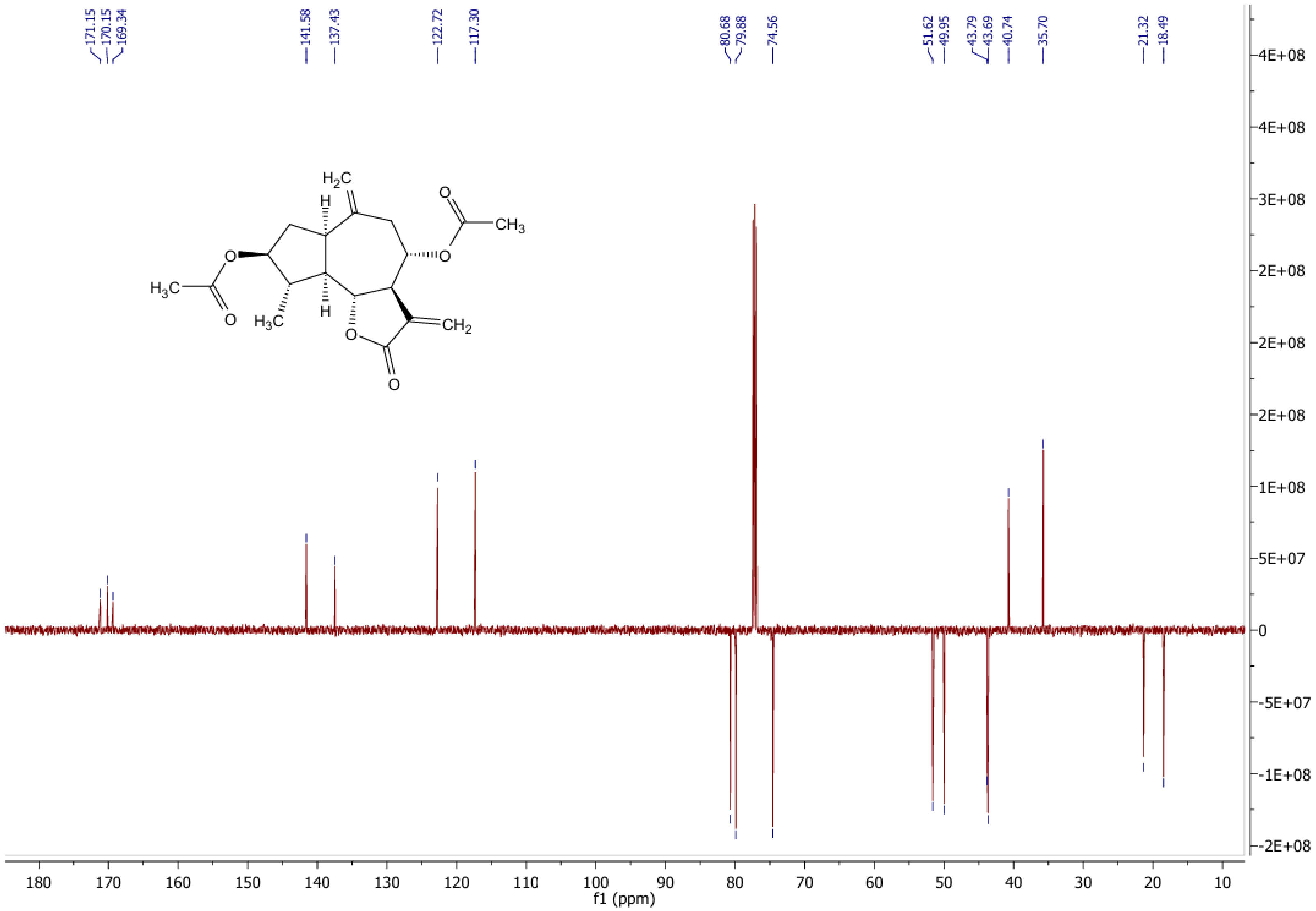

^{1}H-NMR of *N-(((3R,3aR,4S,6aR,8S,9S,9aR,9bR)-4,8-dihydroxy-9-methyl-6-methylene-2-oxododecahydroazuleno[4,5-b]furan-3-yl)methyl)formamide* **14**

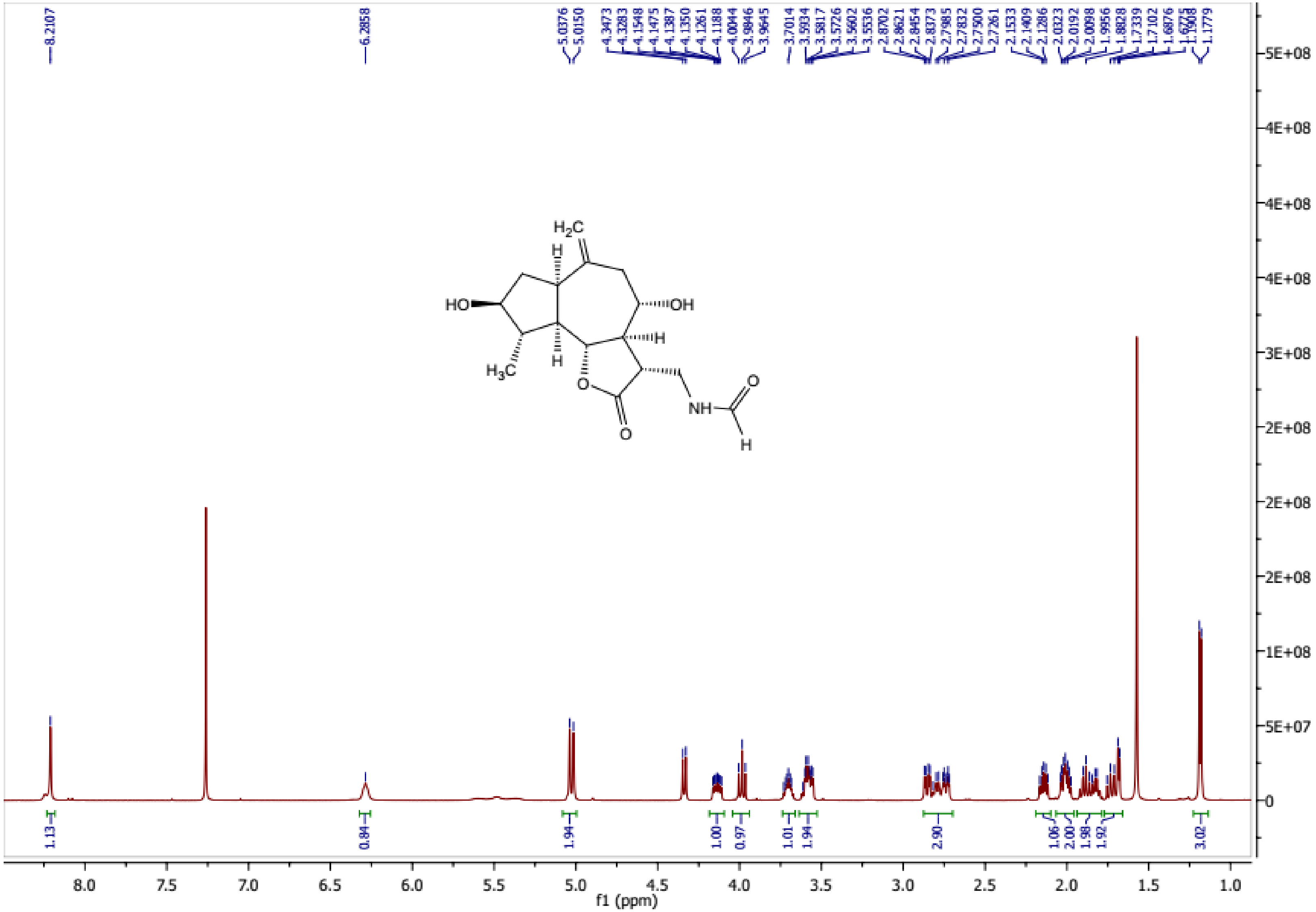

^{13}C-NMR of *N-(((3R,3aR,4S,6aR,8S,9S,9aR,9bR)-4,8-dihydroxy-9-methyl-6-methylene-2-oxododecahydroazuleno[4,5-b]furan-3-yl)methyl)formamide* **14**

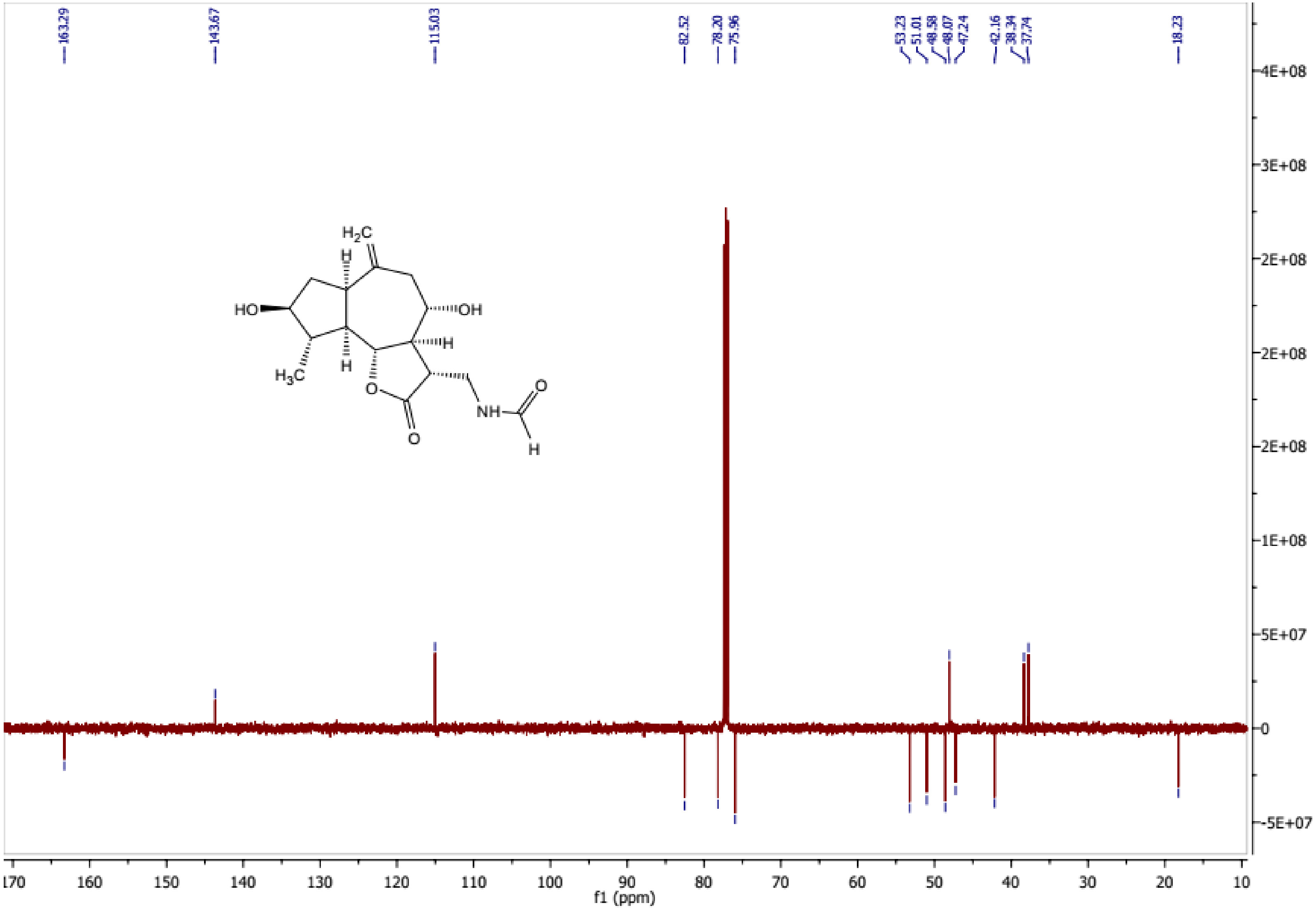

^{1}H-NMR of *(3R,3aR,4S,6aR,8S,9S,9aR,9bR)-4,8-dihydroxy-3-(methoxymethyl)-9-methyl-6-methylenedecahydroazuleno[4,5-b]furan-2(9bH)-one* **15**

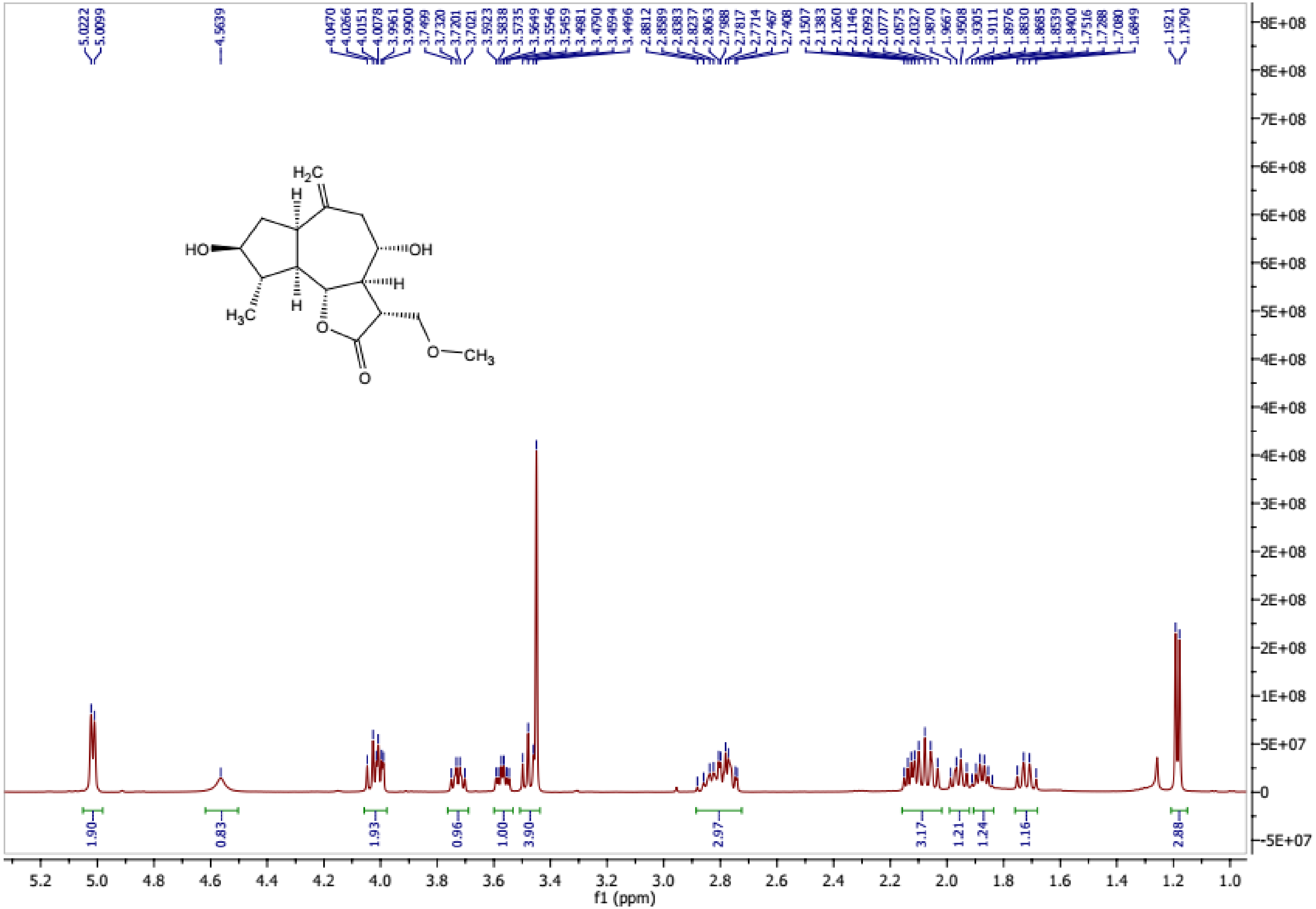

^{13}C-NMR of *(3R,3aR,4S,6aR,8S,9S,9aR,9bR)-4,8-dihydroxy-3-(methoxymethyl)-9-methyl-6-methylenedecahydroazuleno[4,5-b]furan-2(9bH)-one* **15**

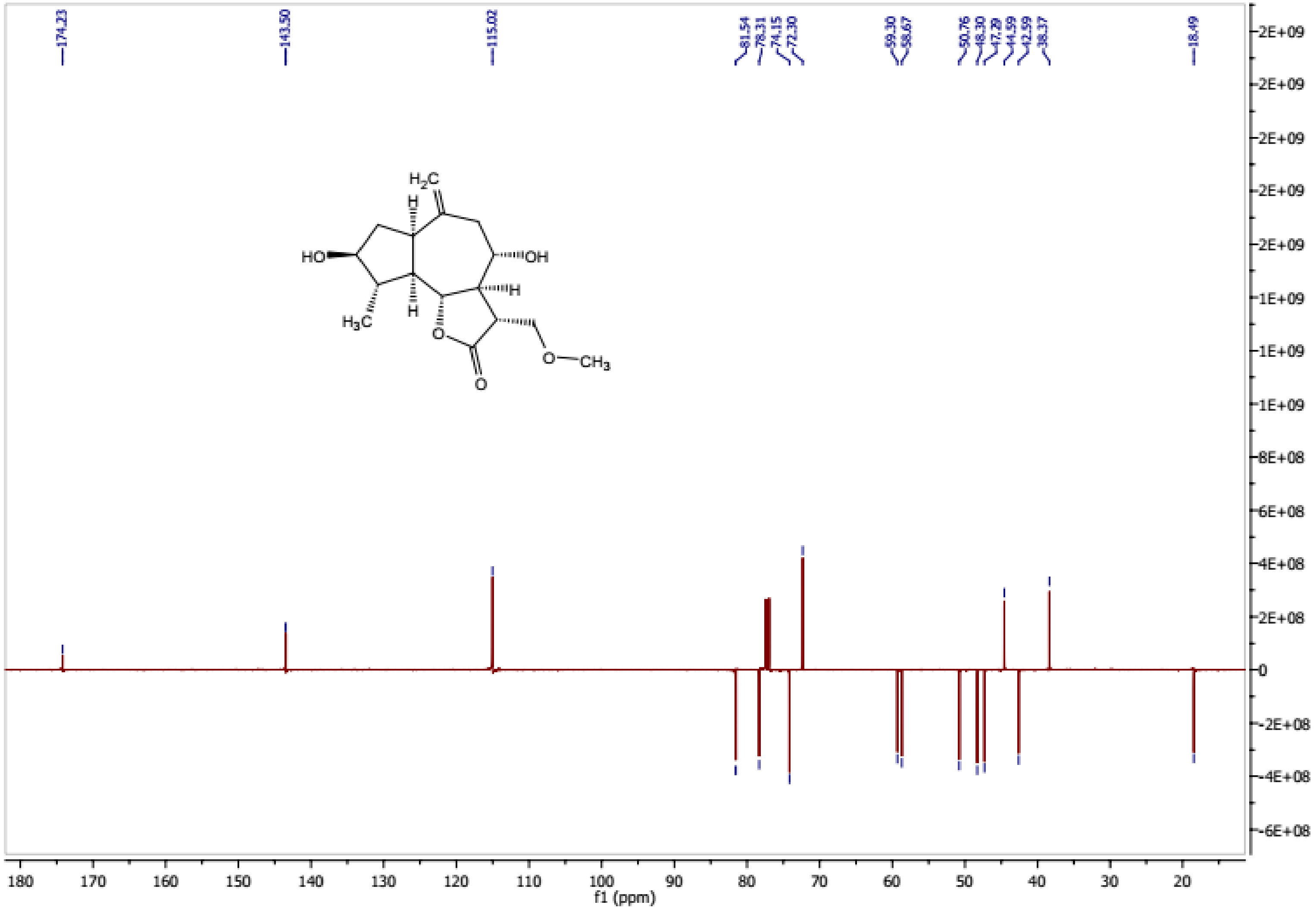

^{1}H-NMR of *(3R,3aR,4S,6aR,8S,9S,9aR,9bR)-3-((1H-benzo[d]imidazol-1-yl)methyl)-4,8-dihydroxy-9-methyl-6-methylenedecahydroazuleno[4,5-b]furan-2(9bH)-one* **16a**

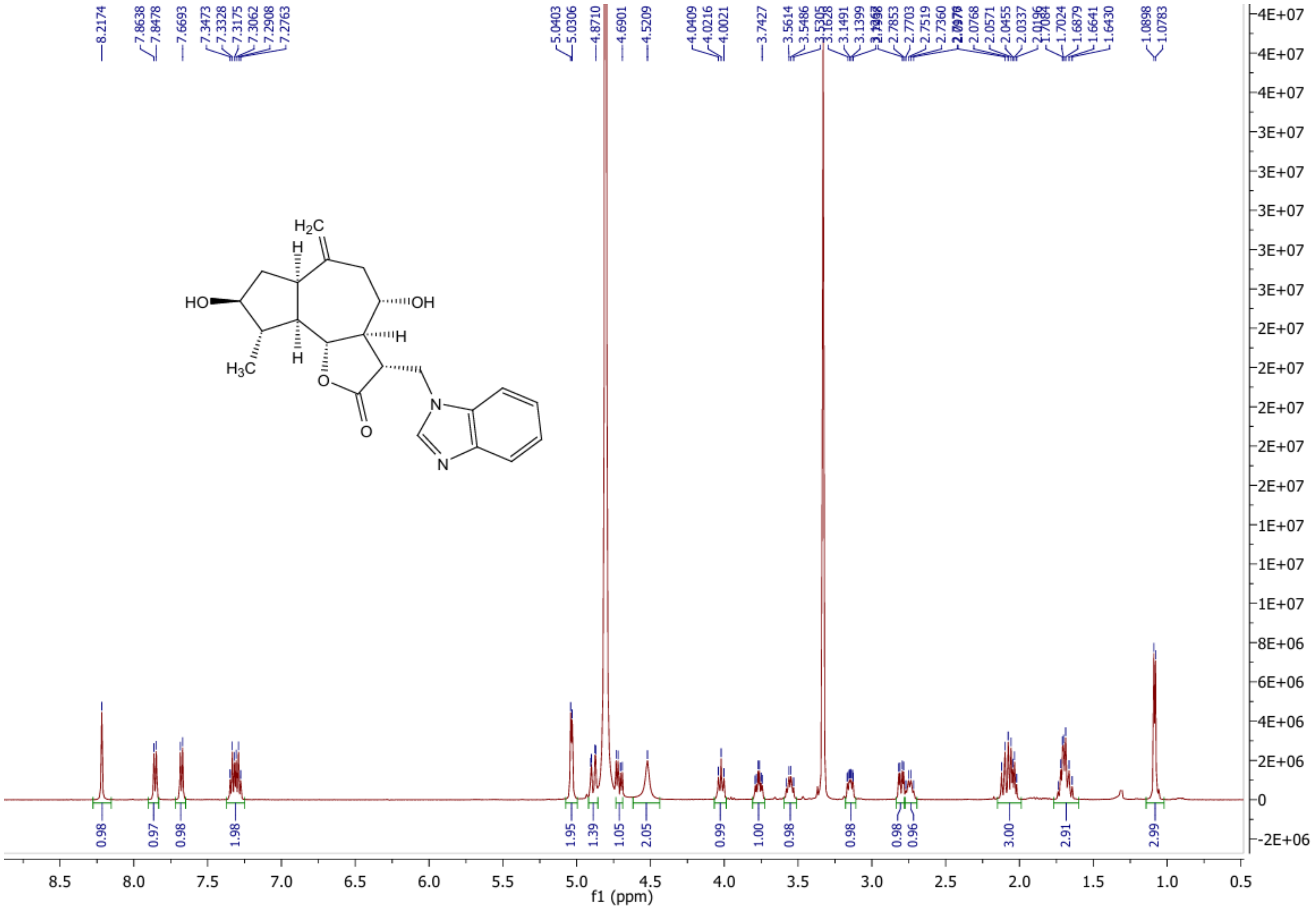

^{13}C-NMR of *(3R,3aR,4S,6aR,8S,9S,9aR,9bR)-3-((1H-benzo[d]imidazol-1-yl)methyl)-4,8-dihydroxy-9-methyl-6-methylenedecahydroazuleno[4,5-b]furan-2(9bH)-one* **16a**

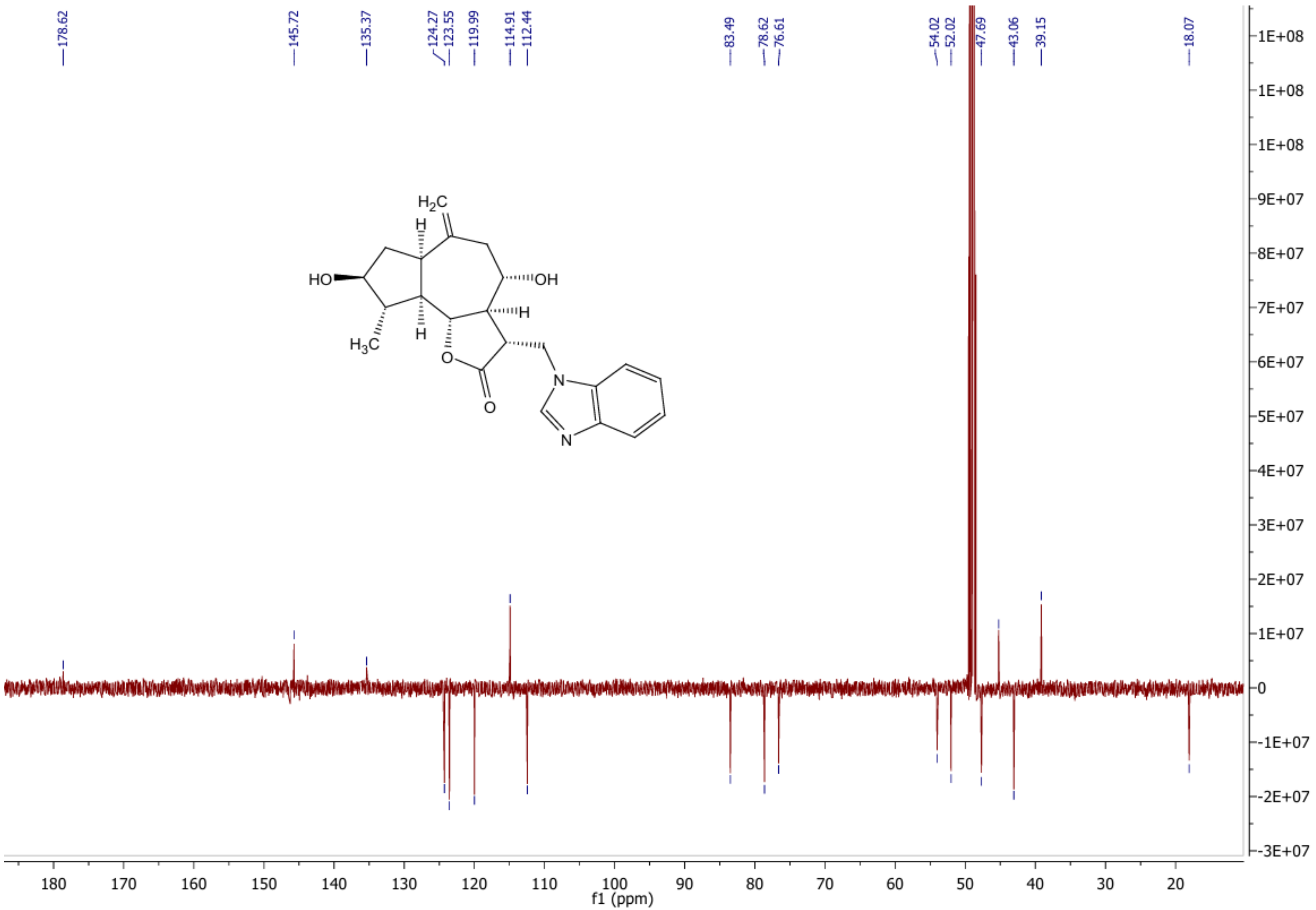

COSY spectrum of **16a**

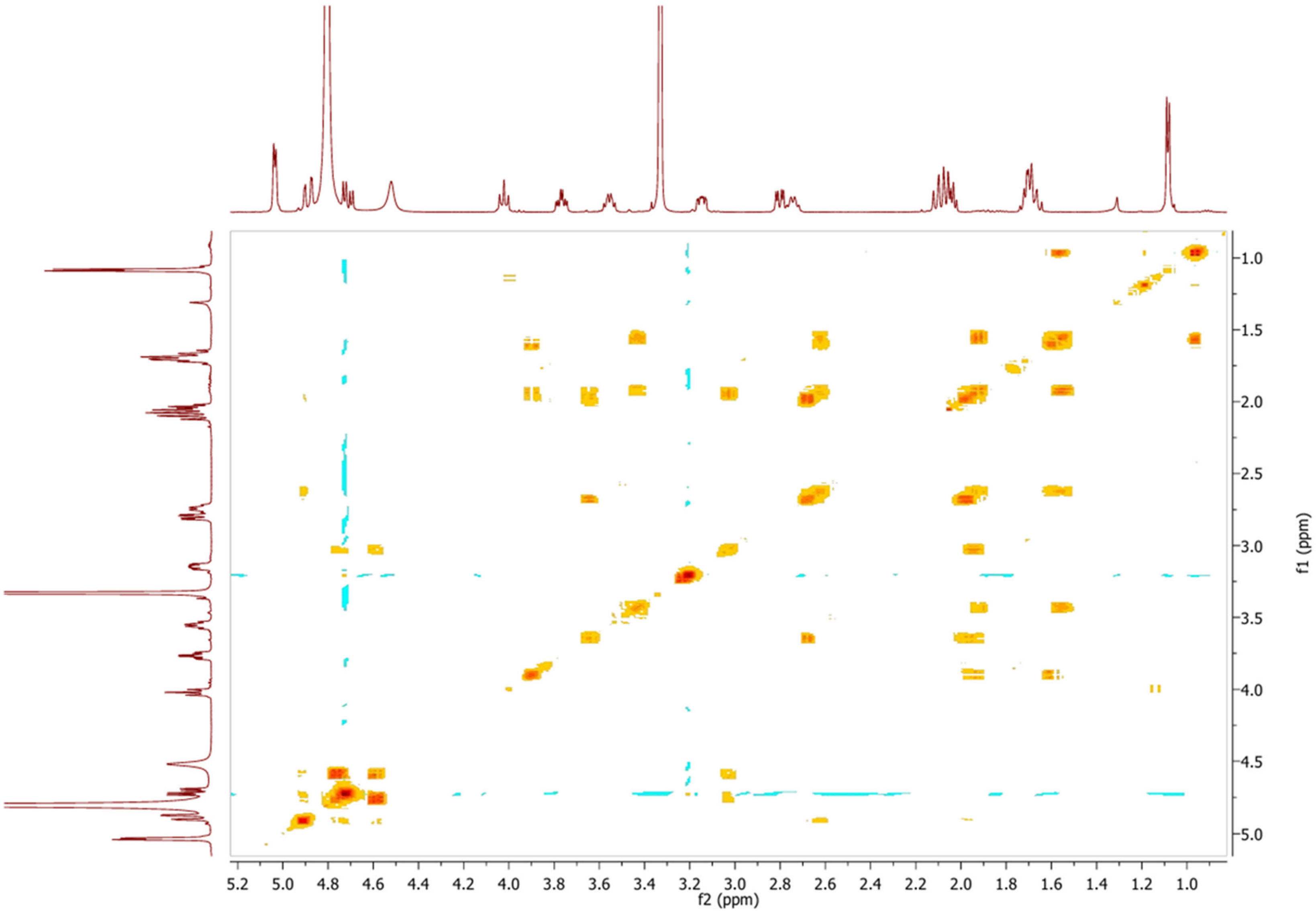

NOESY spectrum of **16a**

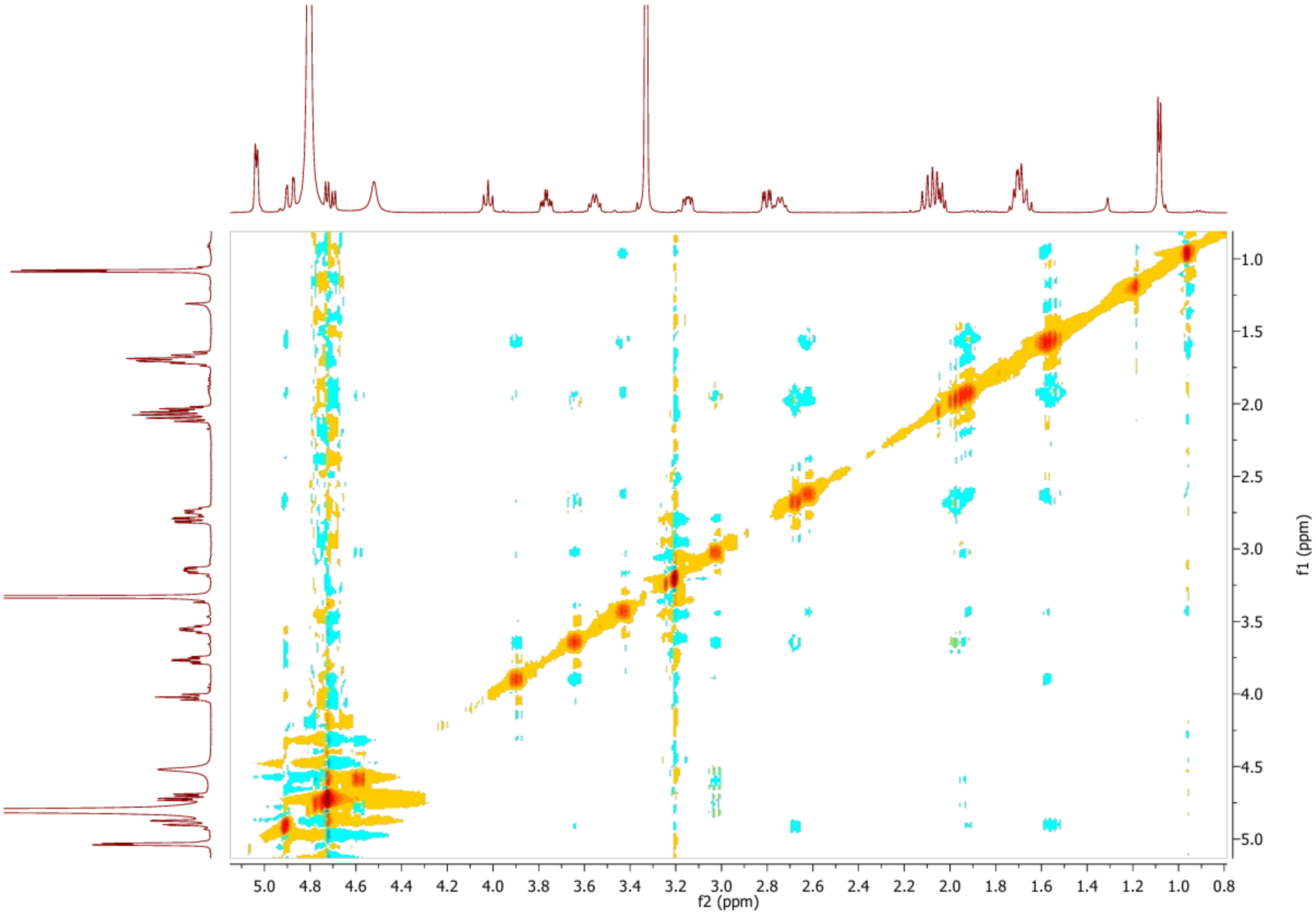

HSQC spectrum of **16a**

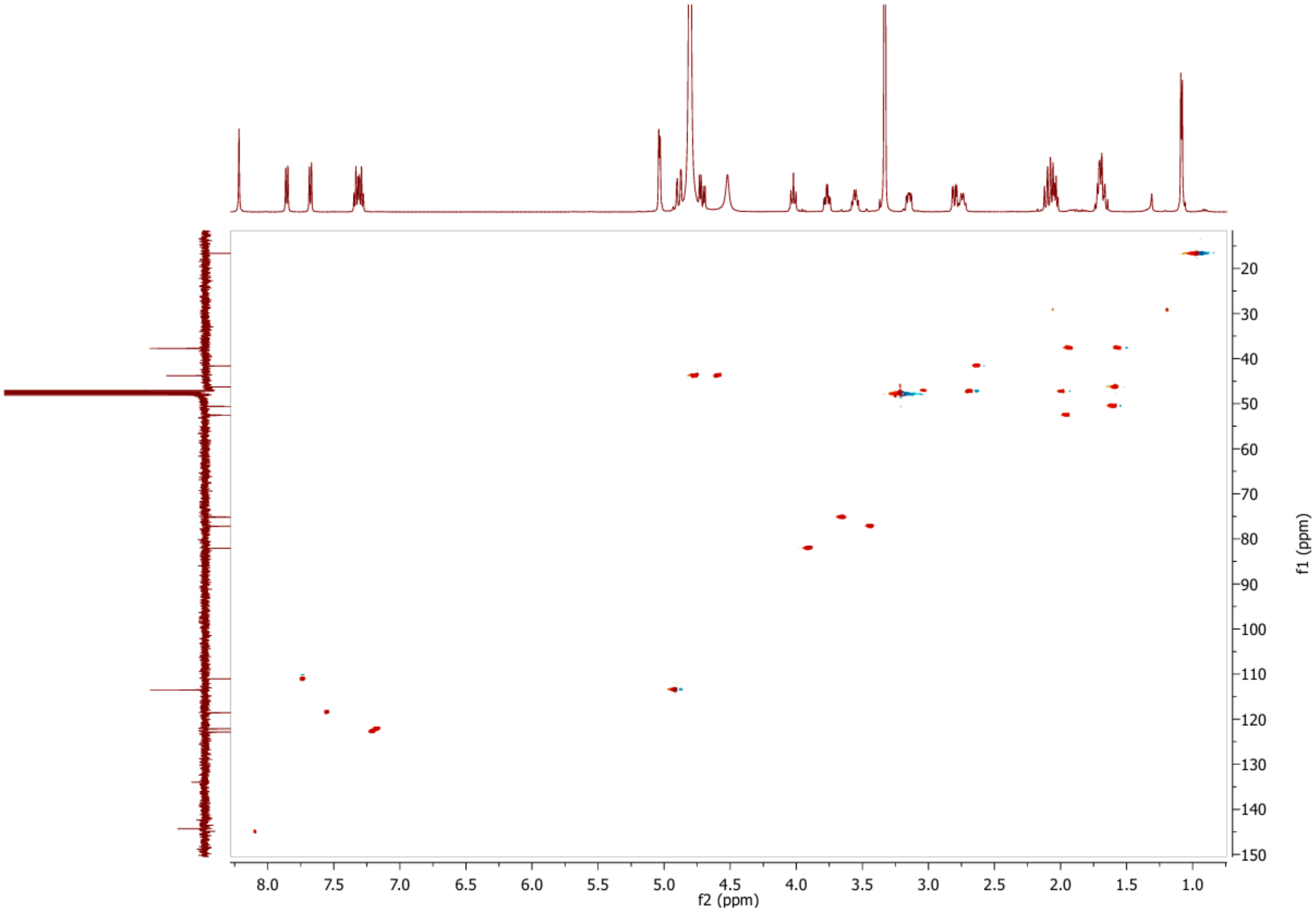

HMBC of compound **16a**

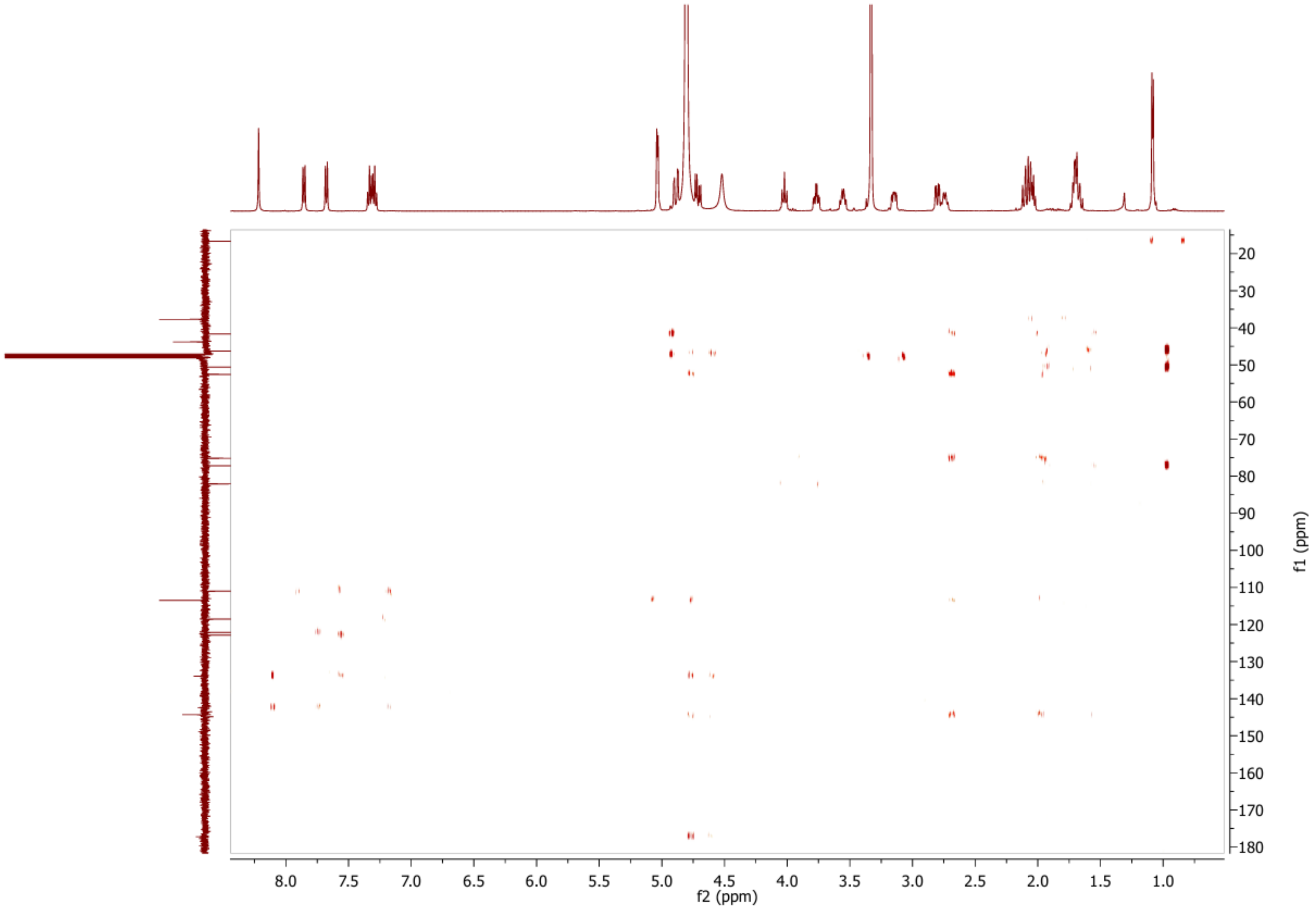

^{1}H-NMR of *(3R,3aR,4S,6aR,8S,9S,9aR,9bR)-3-((1H-indazol-1-yl)methyl)-4,8-dihydroxy-9-methyl-6-methylenedecahydroazuleno[4,5-b]furan-2(9bH)-one* **16b**

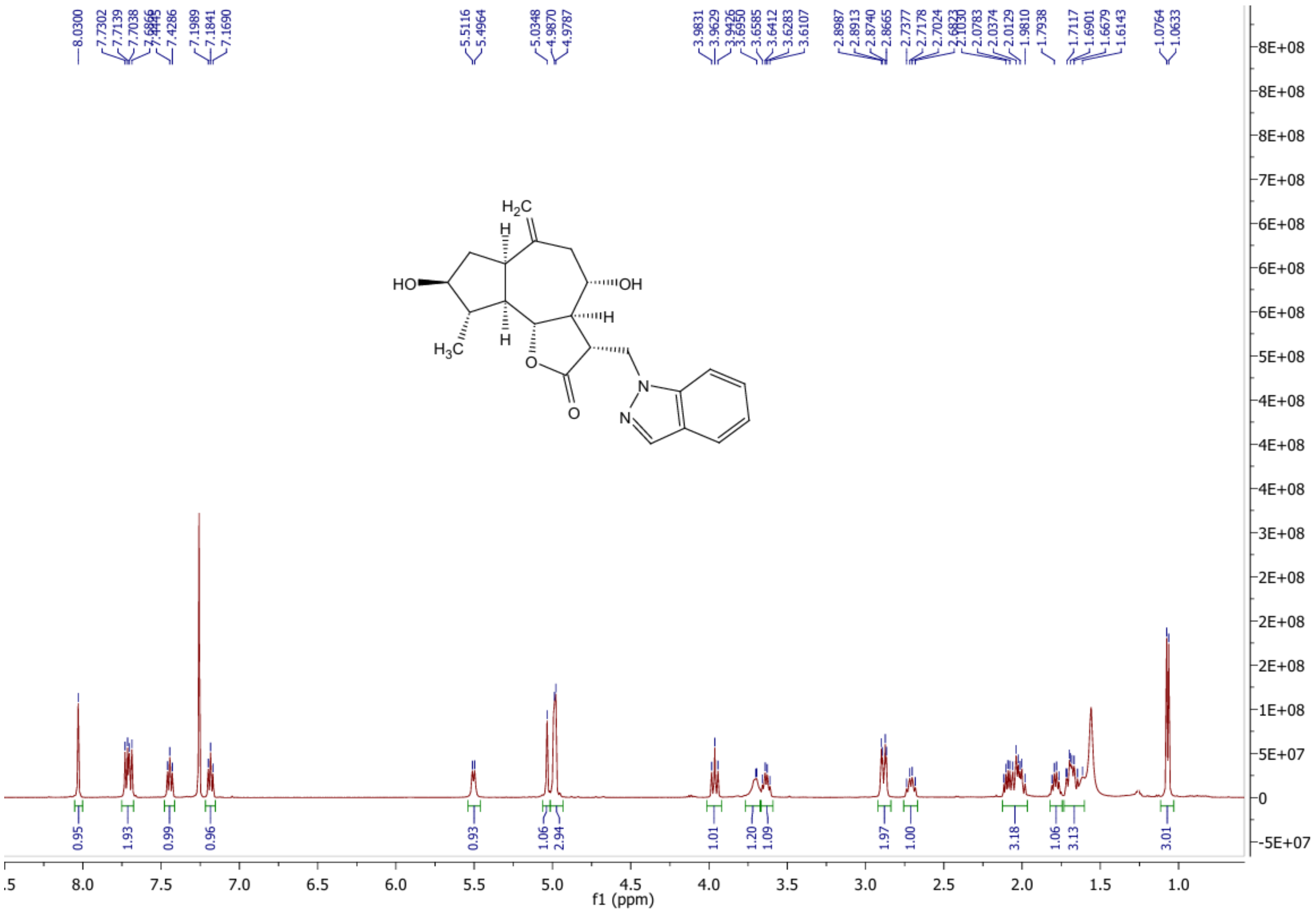

^{13}C-NMR of *(3R,3aR,4S,6aR,8S,9S,9aR,9bR)-3-((1H-indazol-1-yl)methyl)-4,8-dihydroxy-9-methyl-6-methylenedecahydroazuleno[4,5-b]furan-2(9bH)-one* **16b**

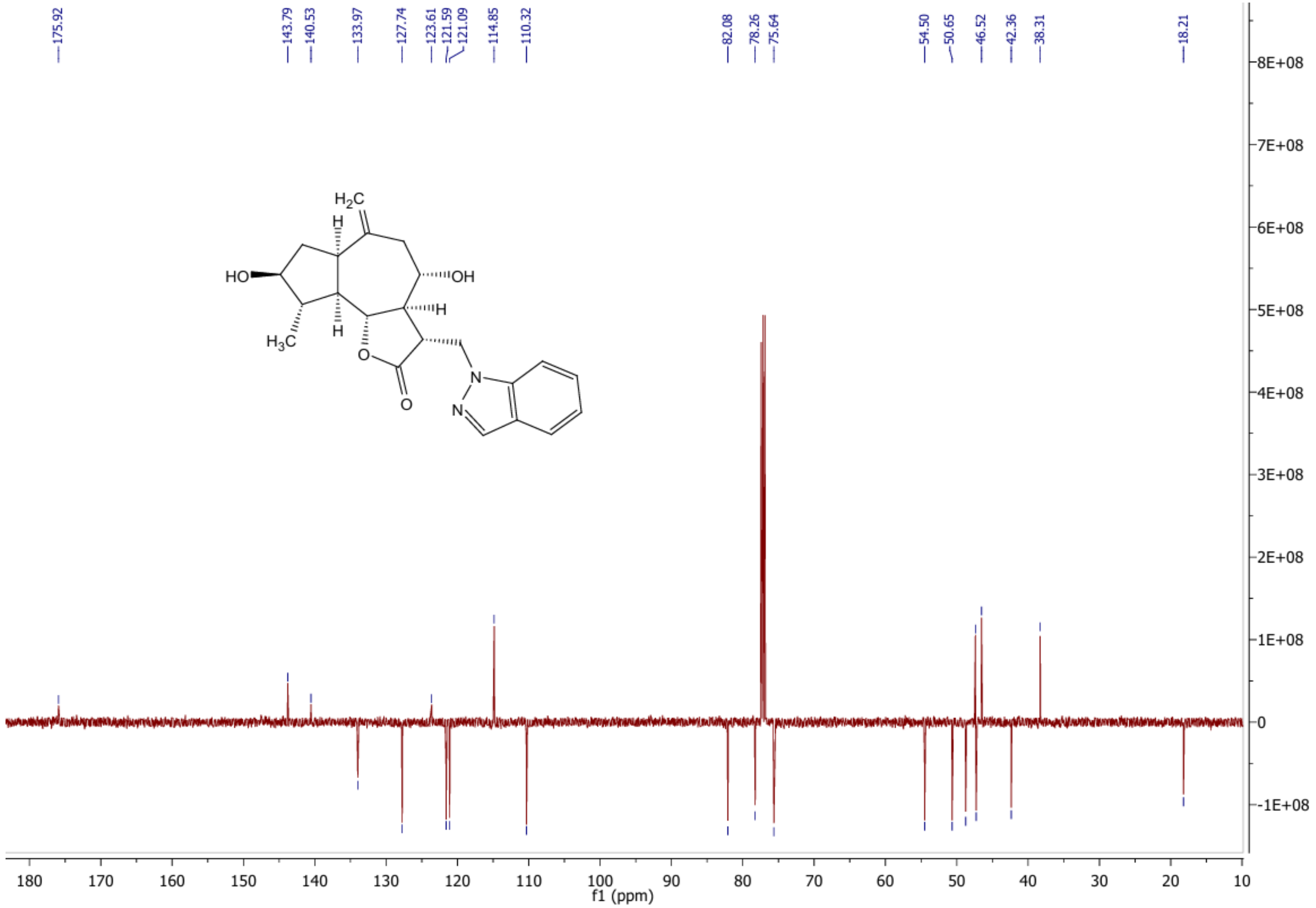

^{1}H-NMR of *(3R,3aR,4S,6aR,8S,9S,9aR,9bR)-3-((1H-benzo[d][1,2,3]triazol-1-yl)methyl)-4,8-dihydroxy-9-methyl-6-methylenedecahydroazuleno[4,5-b]furan-2(9bH)-one* **16c**

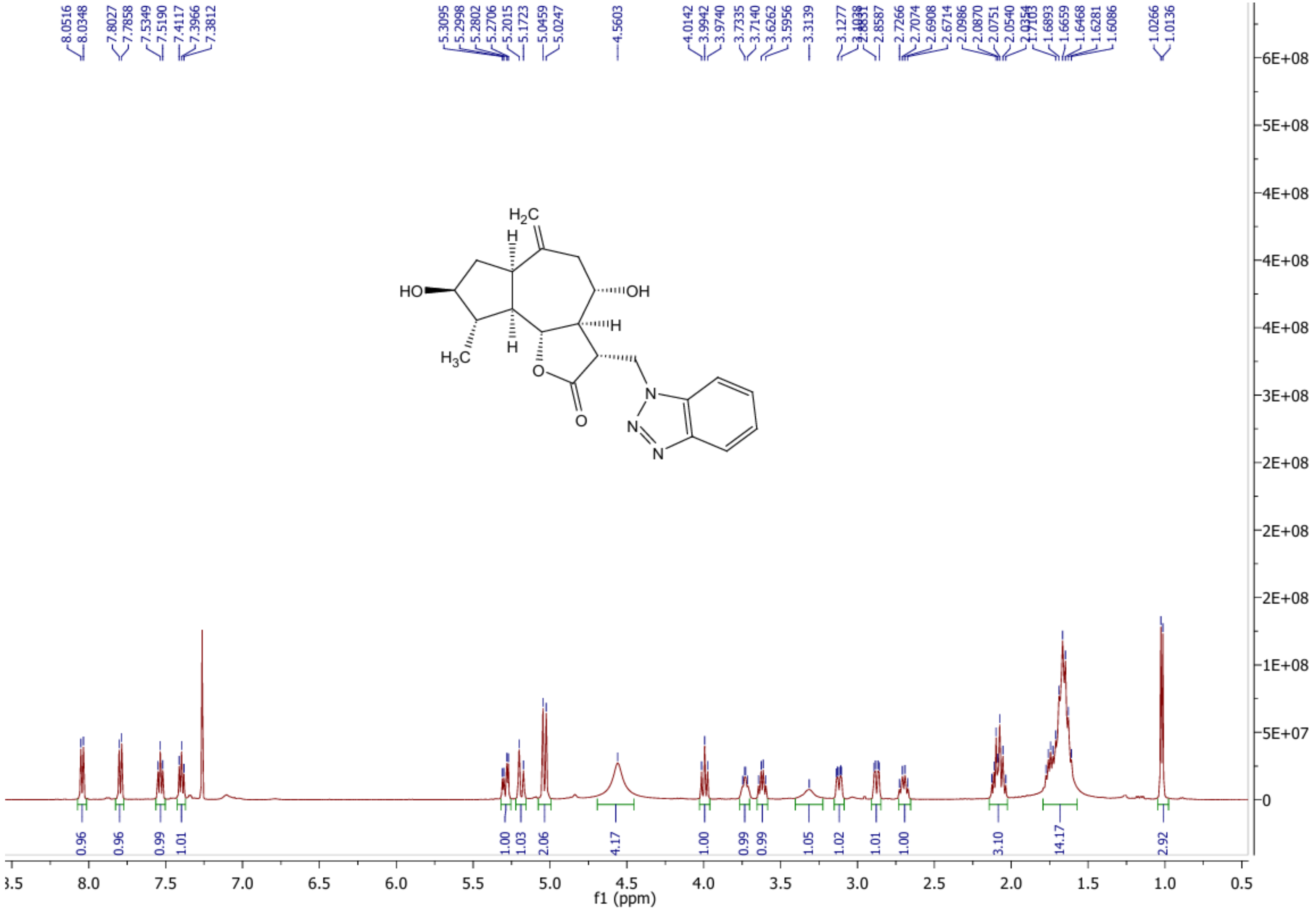

^{13}C-NMR of *(3R,3aR,4S,6aR,8S,9S,9aR,9bR)-3-((1H-benzo[d][1,2,3]triazol-1-yl)methyl)-4,8-dihydroxy-9-methyl-6-methylenedecahydroazuleno[4,5-b]furan-2(9bH)-one* **16c**

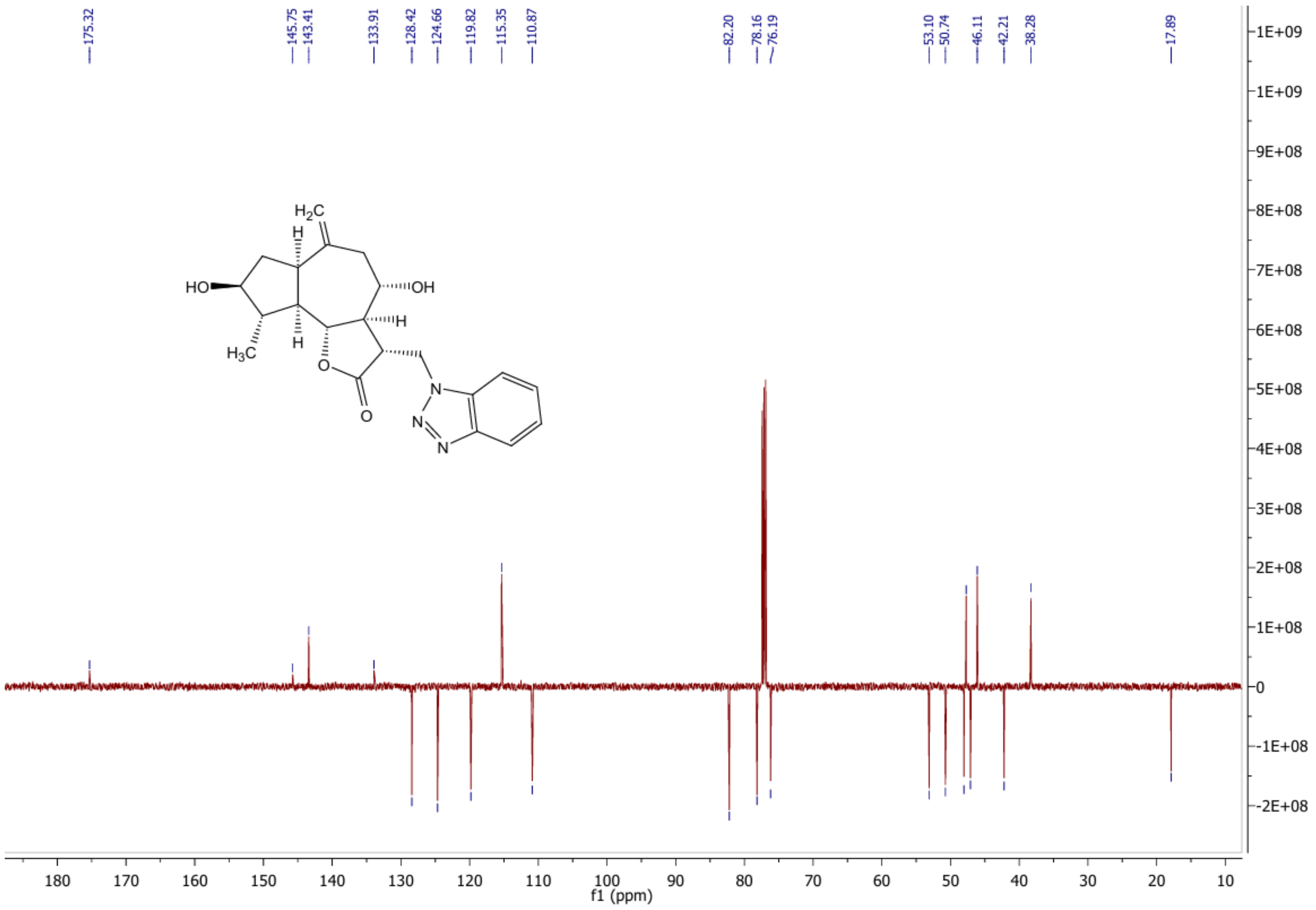

5. Docking study

Table S2. CDocker energy values for compounds **2**, **12** and **13**

**Compounds**	**4DEE**		**4ZJI**		**4ALW**		**1SAO**	
	Energy	Interaction	Energy	Interaction	Energy	Interaction	Energy	Interaction
**2**	-39.8	2	-41.0	4	-34.8	1	-42.4	4
**12**	-39.4	3	-37.2	2	-34.8	2	-38.9	6
**13**	-47.0	3	-45.5	4	-43.3	2	-50.8	5

Table S3. *In-silico* ADMET study

Compounds	*In silico* ADMET properties					
	BBB level	Absorption level	Solubility level	CYP2D6 inhibition	PPB level	Hepatotoxicity
2	3 (high)	0 (good)	3 (good)	F	F	F
12	3 (high)	0 (good)	3 (good)	F	T	T
13	3 (high)	0 (good)	3 (good)	F	T	F

Figure S1. Plot of Polar Surface Area (PSA) vs. LogP for a standard and test set showing the 95% and 99% confidence limit ellipses corresponding to the Blood Brain Barrier (BBB) and Intestinal Absorption models of compounds **2**, **12** and **13**

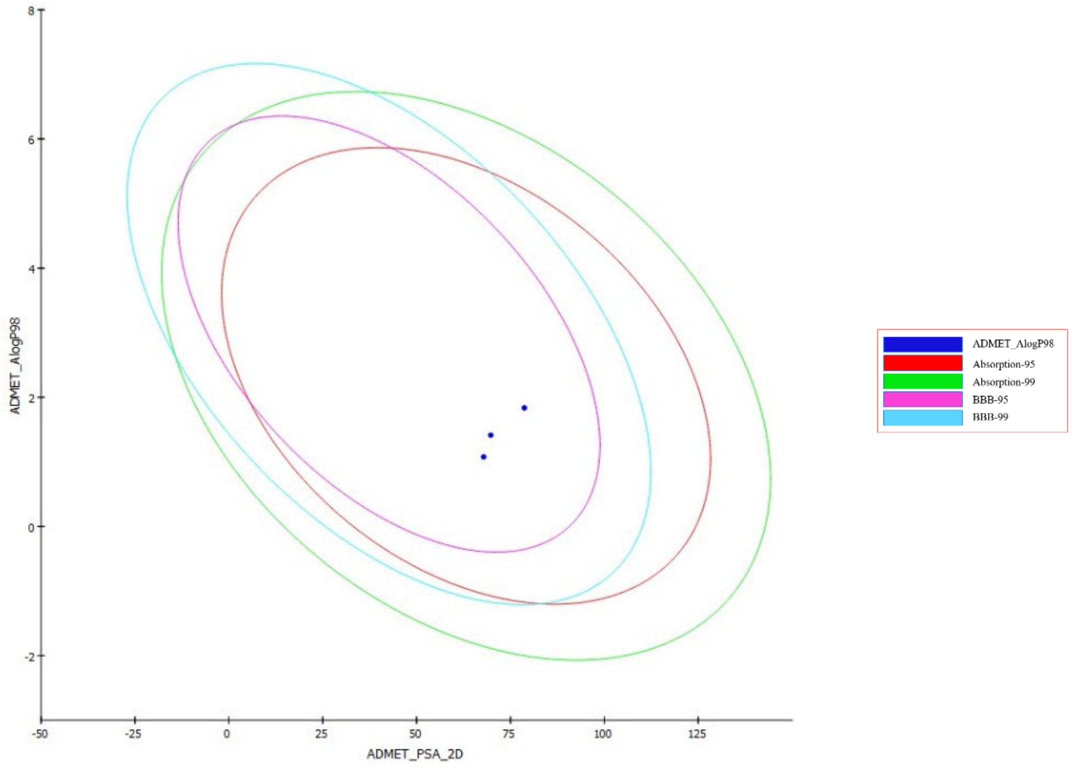

Supplement: Supplementary file 1 [file biomolecules-15-00578-s001.zip › biomolecules-3472161-supplementary.pdf]
